# A deep network DeepOpacityNet for detection of cataracts from color fundus photographs

**DOI:** 10.1038/s43856-023-00410-w

**Published:** 2023-12-16

**Authors:** Amr Elsawy, Tiarnan D. L. Keenan, Qingyu Chen, Alisa T. Thavikulwat, Sanjeeb Bhandari, Ten Cheer Quek, Jocelyn Hui Lin Goh, Yih-Chung Tham, Ching-Yu Cheng, Emily Y. Chew, Zhiyong Lu

**Affiliations:** 1 National Center for Biotechnology Information, National Library of Medicine, National Institutes of Health, Bethesda, MD 20894 USA; 2 Division of Epidemiology and Clinical Applications, National Eye Institute, National Institutes of Health, Bethesda, MD 20892 USA; 3 Singapore Eye Research Institute, Singapore National Eye Centre, Singapore, Singapore; 4 Ophthalmology & Visual Sciences Academic Clinical Program (Eye ACP), Duke-NUS Medical School, Singapore, Singapore; 5 Centre for Innovation and Precision Eye Health & Department of Ophthalmology, Yong Loo Lin School of Medicine, National University of Singapore, Singapore, Singapore

**Keywords:** Lens diseases, Pathology

## Abstract

**Background:**

Cataract diagnosis typically requires in-person evaluation by an ophthalmologist. However, color fundus photography (CFP) is widely performed outside ophthalmology clinics, which could be exploited to increase the accessibility of cataract screening by automated detection.

**Methods:**

DeepOpacityNet was developed to detect cataracts from CFP and highlight the most relevant CFP features associated with cataracts. We used 17,514 CFPs from 2573 AREDS2 participants curated from the Age-Related Eye Diseases Study 2 (AREDS2) dataset, of which 8681 CFPs were labeled with cataracts. The ground truth labels were transferred from slit-lamp examination of nuclear cataracts and reading center grading of anterior segment photographs for cortical and posterior subcapsular cataracts. DeepOpacityNet was internally validated on an independent test set (20%), compared to three ophthalmologists on a subset of the test set (100 CFPs), externally validated on three datasets obtained from the Singapore Epidemiology of Eye Diseases study (SEED), and visualized to highlight important features.

**Results:**

Internally, DeepOpacityNet achieved a superior accuracy of 0.66 (95% confidence interval (CI): 0.64–0.68) and an area under the curve (AUC) of 0.72 (95% CI: 0.70–0.74), compared to that of other state-of-the-art methods. DeepOpacityNet achieved an accuracy of 0.75, compared to an accuracy of 0.67 for the ophthalmologist with the highest performance. Externally, DeepOpacityNet achieved AUC scores of 0.86, 0.88, and 0.89 on SEED datasets, demonstrating the generalizability of our proposed method. Visualizations show that the visibility of blood vessels could be characteristic of cataract absence while blurred regions could be characteristic of cataract presence.

**Conclusions:**

DeepOpacityNet could detect cataracts from CFPs in AREDS2 with performance superior to that of ophthalmologists and generate interpretable results. The code and models are available at https://github.com/ncbi/DeepOpacityNet (10.5281/zenodo.10127002).

## Plain language summary

Cataracts are cloudy areas in the eye that impact sight. Diagnosis typically requires in-person evaluation by an ophthalmologist. In this study, a computer program was developed that can identify cataracts from specialist photographs of the eye. The computer program successfully identified cataracts and was better able to identify these than ophthalmologists. This computer program could be introduced to improve the diagnosis of cataracts in eye clinics.

## Abstract (web summary)

Elsawy, Keenan, Chen et al. detect cataracts from color fundus photography using an explainable deep learning network called DeepOpacityNet. DeepOpacityNet detects cataracts more accurately than ophthalmologists and demonstrates that the absence of blood vessels is an indicator that cataracts are present.

## Introduction

Cataract is the leading cause of blindness worldwide and accounts for half of global blindness^1–3^. It forms as an opacity in the crystalline lens that typically develops slowly and causes visual impairment. In its advanced stages, it causes severe visual impairment and requires surgical extraction with intraocular lens implantation. Age-related cataract has three main types, according to the location of the opacity: nuclear sclerosis (NS), cortical lens opacity (CLO), and posterior subcapsular cataract (PSC), with NS cataract being the most common type^4–8^. All three types become increasingly prevalent with older age but with partially distinct risk factors, visual symptoms, and rates of progression.

The diagnosis of cataracts usually requires direct assessment of the crystalline lens at the slit-lamp by a trained ophthalmologist. This can be a challenge in particular countries and settings. Potential difficulties may include poor availability of ophthalmologists, large distances required to travel for in-person evaluation, and high consultation costs. Indeed, in low-income countries, the number of ophthalmologists per million population has been estimated to be as low as 3.7, compared to a mean of 76.2 in high-income countries^9^. Unsurprisingly, inverse correlations have been observed between ophthalmologist density and the prevalence of blindness^9^. Moreover, cataract prevalence is predicted to increase because of the aging population in many countries^10–12^, which will exacerbate the problem of many patients remaining undiagnosed in low-income countries^13,14^. Even in middle/high-income countries, there may be many missed opportunities for the diagnosis of cataracts. Color fundus photography (CFP) is often performed in the absence of in-person evaluation by an ophthalmologist. For example, during diabetic retinopathy (DR) screening in most countries, no ophthalmologist is present, so symptomatic cataracts may only be detected by poor visibility of the CFP. In the future, this phenomenon may increase; for example, CFP might be performed more frequently in the primary care setting or at optometry appointments. As the application of CFP expands to include primary care and general optometry settings, increased opportunities for cataract detection on CFP will arise.

CFP has multiple advantages as an imaging modality: it is used very widely across the world (including in many low-income countries), is inexpensive, requires only simple technology, and can be obtained with minimal training. Increasingly, CFP can be performed with handheld and even smartphone-based fundus cameras^15,16^. However, cataracts cannot be reliably diagnosed by ophthalmologists from CFP. For these reasons, developing an automated cataract screening tool that could detect cataracts from CFP could greatly increase accessibility to cataract screening^17^. This could be an important adjunct to DR screening programs. Through telemedicine approaches, it could also represent a convenient and cost-effective method for cataract screening in rural places or developing countries, since images could be obtained and screened in the community^18^.

Deep learning as a subfield of artificial intelligence has become the state-of-the-art method for computer vision. The power of deep-learning methods comes from their ability to extract a hierarchal set of non-linear features that are descriptive to solve complex tasks^19^. For tasks such as cataract detection, deep learning is of great interest, since it has shown high levels of performance in detection and classification tasks in medicine, as well as in highlighting the image features that contribute most to decision-making^17,18,20–22^.

The primary aim of the study was to use the Age-Related Eye Disease Study 2 (AREDS2)^23,24^ dataset to develop and evaluate DeepOpacityNet, a deep-learning model for cataract detection from CFPs, and compare its performance to that of three ophthalmologists. The secondary aim was to visualize the salient features associated with cataract presence/absence in CFPs, as detected by DeepOpacityNet.

The results of this study show that DeepOpacityNet has outperformed other state-of-the-art methods on the internal test set and outperformed the performance of three ophthalmologists on a subset of the test set. Also, DeepOpacityNet could generalize on three external datasets with high performance. Visualizations of DeepOpacityNet show that visibility of blood vessels was associated with cataract absence while blurred regions were associated with cataract presence. Thus, this work could be used to improve the diagnosis of cataracts in eye clinics.

## Methods

### Datasets

The dataset of CFPs used for this study was curated from the AREDS2^23^. In summary, the AREDS2 was a multi-center, phase 3, randomized clinical trial designed to study the effects of nutritional supplements on the course of age-related macular degeneration (AMD) in people at moderate to high risk of progression to late AMD and age-related cataract^23,25,26^. A total of 4203 participants, 50 to 85 years old, were recruited between 2006 and 2008 at 82 retinal specialty clinics in the United States. The eligibility criteria were participants with either bilateral large drusen or late AMD in one eye and large drusen in the fellow eye. The participants were followed for five years. At baseline and annual study visits, eye examinations were performed by certified study personnel using standardized protocols. Digital stereoscopic CFP images were obtained at all study visits by certified technicians using standard imaging protocols. In this study, the field 2 CFPs (i.e., with a 30° imaging field centered at the fovea) were used. The study was approved by the institutional review boards of the study sites (i.e., 82 clinical sites across the United States). The study adhered to the tenets of the Declaration of Helsinki and complied with the Health Insurance Portability and Accountability Act. Written informed consent was obtained from all participants at all the study centers (see Supplementary Note 1).

The ground truth labels used for model training and testing were obtained by transferring and thresholding the grades previously assigned by human experts to each eye at each visit. For NS cataracts, grading was performed by the AREDS2 investigators who were performing the clinical assessment and slit-lamp examination of the participant (i.e., no arbitration was required). Grading was done by comparison with standard photographs, with severity levels: <1, 1, 1.5, 2, 2.5, 3, >3. For CLO and PSC cataracts, grading was performed by human expert graders at the AREDS Reading Center (i.e., University of Wisconsin) from study photographs. Only one grader evaluated each pair of eyes, such that no arbitration was required. The reading center did perform intra-grader and inter-grader agreement studies^27^. The reading center grading protocol and definitions have been described by Domalpally et al.^27^. Shortly, CLO and PSC cataracts were graded from stereoscopic fundus reflex photographs, focused on the lens, based on percentage area involvement of a central circle with diameter 5 mm. Involved lens areas were those that were definitely darkened, regardless of the density of the opacity.

For each of the three cataract types, clinical thresholds were applied to make each variable binary. Based on AREDS2 Report 31^2^, the ranges used for the clinically significant absence/presence of cataracts were 0‒1.5/2+ for NS, 0‒11.2%/11.3%+ for CLO, and 0‒2.2%/2.3%+ for PSC. Following this, the three variables were converted into one binary variable representing cataract presence if at least one type of cataract was present; otherwise, it was considered absent.

**Fig. 1:**
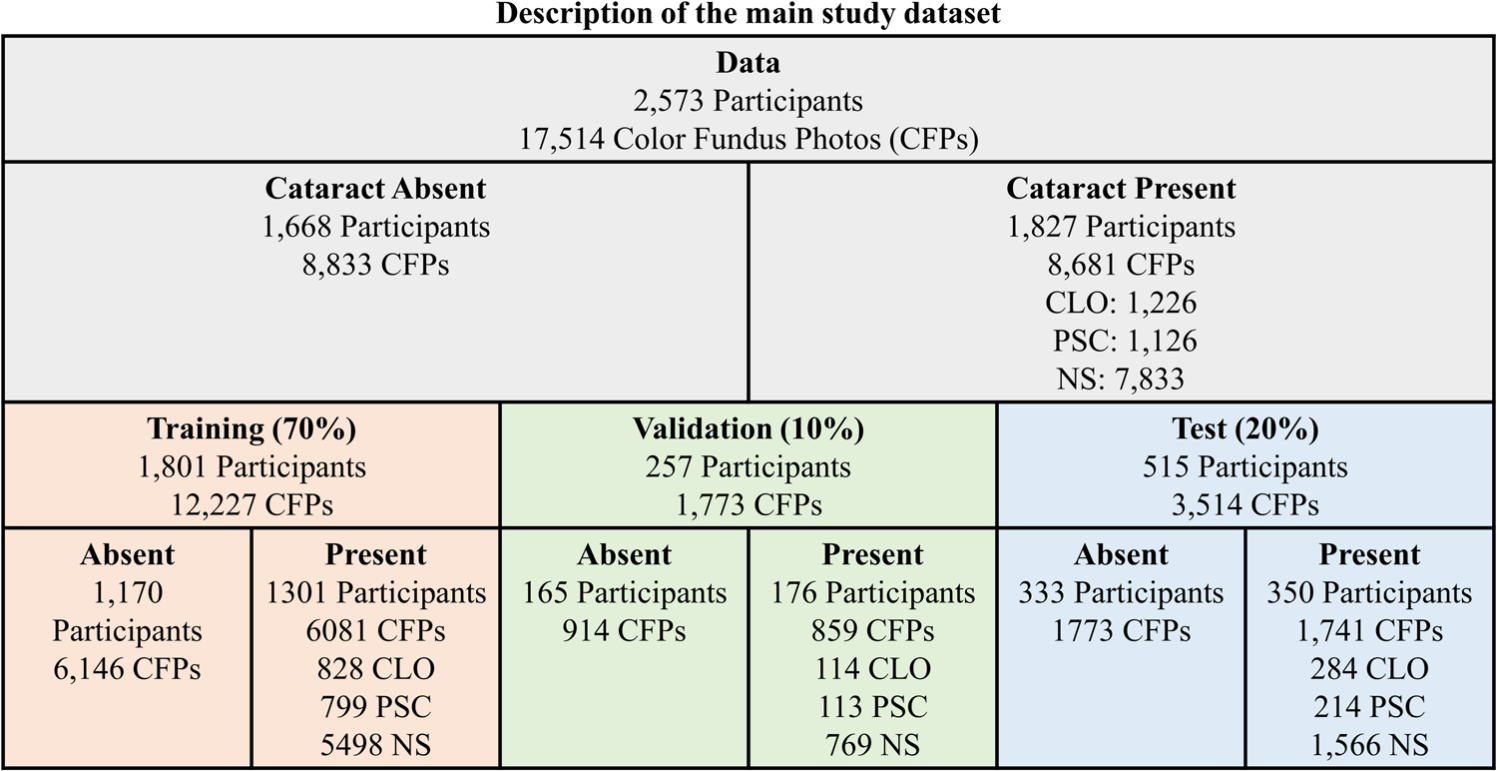
Description of the main study dataset. Characteristics of the study dataset where absent means that the color fundus photographs (CFPs) are labeled with no cataracts and present means that the CFPs are labeled with cataracts. It should be noted that numbers do not add up since some participants have more than one cataract type.

The dataset consisted of 17,514 field 2 CFPs (i.e., centered on the fovea) from 2573 participants. The participants in our dataset included 96% white and 55% females with a mean (SD) age of 69.84 (7.72). All CFPs were from phakic eyes since those from pseudophakic and aphakic eyes were excluded. The description of the dataset is shown in Fig. 1. This comprised 8681 CFPs (from 1668 participants) with cataracts and 8833 CFPs (from 1827 participants) without cataracts (i.e., some participants had CFPs both before and after developing cataracts). The dataset was randomly split, at the participant level, into three independent sets: the training set (70%), the validation set (10%), and the test set (20%). The dataset contained all types of cataracts: nuclear (*n* = 7833), cortical (*n* = 1226), and posterior subcapsular (*n* = 1126).

### External datasets

Three external CFP datasets were obtained from the Singapore Epidemiology of Eye Diseases study (SEED) and used for external validation in this study^28^. All datasets were based on population-based studies and were collected between 2004 and 2011 at the SEED baseline visit and have been de-identified. The first dataset was based on the Singapore Malay Eye Study (SiMES) and contained 5,752 CFPs^29^. The second dataset was based on the Singapore Chinese Eye Study (SCES) and contained 5,745 CFPsx^30^. The third dataset was based on the Singapore India Eye Study (SINDI) and contained 5,591 CFPs^31^. All three studies followed the principles of the Declaration of Helsinki and were approved by the Singapore Eye Research Institute (SERI) Institutional Review Board (IRB). Informed written consent was obtained from all participants and approved by the SERI IRB^29–31^.

In all three studies, participants underwent digital lens imaging, comprising 45-degree slit-lamp images and retroillumination images. NS severity was graded from the slit-lamp images using a range of 1–5 following the Wisconsin Cataract Grading System. Both CLO and PSC severity was graded from the retroillumination images using a range of 0-100% following the Wisconsin Cataract Grading System^28^. For NS, the severity grades were converted to the AREDS2 NS severity scale. For the CLO and PSC severity grades, no conversion was needed. Similar to the AREDS2 dataset itself, the ground truth labels were obtained by transferring and thresholding the severity grades to the CFPs, using the same binary thresholds that defined cataract presence or absence. After thresholding, the SiMES, SCES, and SINDI datasets had 1,271, 913, and 821 CFPs labeled with cataract presence, respectively. More details about the characteristics of the three datasets are in Supplementary Table 1.

The data analysis involving the SEED dataset was conducted by the SEED research team led by Cheng C-Y, the lead principal investigator of the SEED study. In brief, the NEI/NLB study team provided the codes of the deep-learning algorithms in GitHub, which Dr. Cheng’s team then executed within their secure systems at the Singapore Eye Research Institute (SERI), ensuring that the SEED dataset remained under their custodianship. Subsequently, the AI-generated output scores and results were provided to the NEI/ NLB study team. This method was in strict adherence to the data governance policies established by SERI. The permission for the use of SEED data in this manner was specially granted by SEED PI Dr. Cheng for this research collaboration and does not extend beyond the scope of this study.

### Image preprocessing

The CFPs in the AREDS2^23^ dataset have different shapes and sizes. Therefore, to make sure that the retinal structures had approximately the same size in all photographs, we processed the CFPs using two methods: (1) removing any black background regions, and (2) extracting the largest inscribed square within the fundus region. Then, the processed CFP was resized to 384 × 384 pixels. To enhance the retinal structures and decrease the effects of different illumination levels between CFPs, Contrast Limited Adaptive Histogram Equalization (CLAHE)^32^ was applied to each channel of the input image (see Supplementary Fig. 1). We performed a comparison to see the effectiveness of each preprocessing method as well as the CLAHE method. Additionally, the images were randomly augmented using horizontal flipping, vertical flipping, and transposing during the training; each with a probability of 0.5 to allow for any combinations of them (see Supplementary Fig. 2).

### DeepOpacityNet

**Fig. 2:**
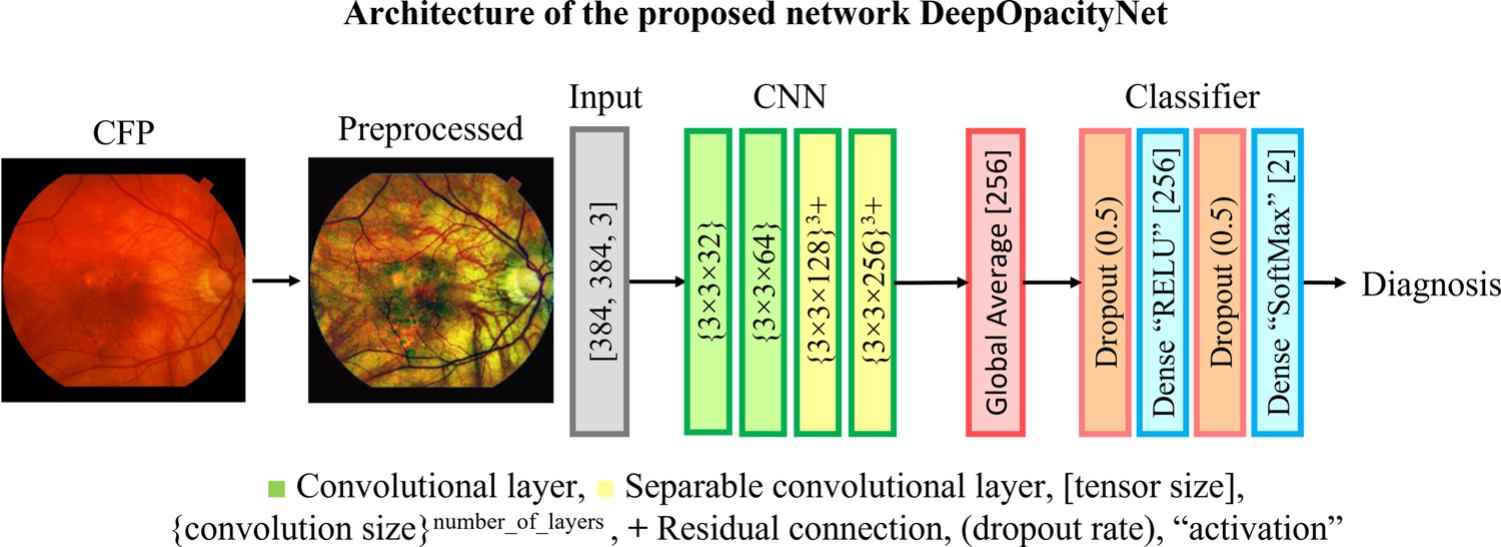
Architecture of the proposed network DeepOpacityNet. The network consists of an input layer (gray block) that takes the preprocessed color fundus photograph (CFP), a convolutional neural network (CNN) that consists of a series of convolutional layers (green and yellow blocks), a global average pooling layer (red block), and a classifier that consists of two dropout layers (orange blocks) and two dense layers (blue blocks).

Our proposed network, DeepOpacityNet, was designed using separable convolutions to limit the number of its parameters and residual connections to enhance the gradient flow. It was designed from scratch for the classification task and was not pre-trained. The details of the architecture of the proposed network are shown in Fig. 2. The proposed network is efficient in its size and number of parameters, so is less likely to overfit (see Supplementary Table 2). To capture fine details without losing too much receptive field, DeepOpacityNet was designed to have a smaller number of levels with more layers in each level. It contains two residual blocks, with three separable convolutional layers in each block. Average pooling is used in the residual connection to reduce the spatial dimensions of the input convolution. Each convolutional layer is followed by batch normalization and an exponential linear unit (ELU)^33^ activation instead of rectified linear units (ReLU), to enhance performance. Using more separable convolutional layers helped to increase the receptive field while keeping a larger spatial size at the final convolutional layer. This helped to maintain fine local features that are important for discrimination and provide visualizations that can be interpreted. For all networks, the SoftMax activation function was used to generate the class likelihood probabilities; the binary predictions were made using the class with the highest probability.

A comprehensive study was performed to show the effectiveness of the network by constructing other networks with a different number of residual blocks and a different number of separable convolutional layers in each block. The networks of the study are denoted by XB*n*L*m*, where X denotes Xception-like, B*n* is the number of residual blocks (i.e., 2, 3, 4, or 5), and L*m* is the number of layers in each block (i.e., 2 or 3). More details are in Supplementary Tables 2 and 3. Based on our experiments, we selected DeepOpacityNet to be XB2L3 with ELU activation function.

A stochastic gradient descent (SGD)^34^ optimizer and a categorical cross-entropy loss function were used to train the network. The hyperparameter settings were batch size of 32, maximum epoch size of 100, learning rate of 0.001, and decay of 0.9. Early stopping was applied if the training loss did not improve for 5 epochs. All experiments were performed using Python 3.8, TensorFlow 2.3, Keras 2.4 deep-learning libraries running on a server with 48 Intel Xeon CPUs with 754 Gb RAM and an NVIDIA GeForce GTX 1080 Ti 32 Gb GPU.

### Performance evaluation and comparison

The performance of all networks was measured using macro-average metrics, together with 95% confidence intervals (CI), including accuracy, precision, recall, F1, kappa (*κ*)^35^, area under the receiver operating characteristic curve (AUC), and average precision (AP). To compute the CI, we used the Monte Carlo method (i.e., bootstrapping) by using 1000 iterations and randomly sampling a number of values > 2 then computing the metric. We computed Cis for the internal test set, which contained 3514 examples. The scores of each metric were sorted and the lower confidence level was set as the value greater than 2.5% (i.e., 0.025 × length of values) and the upper confidence level was set as the value greater than 97.5% (i.e., 0.975 × length of values). We used a fixed random seed to control the reproducibility of the metrics (i.e., 42).

The performance of DeepOpacityNet on cataract detection from CFPs was compared with that of the baseline networks: (1) VGG16^36^, (2) ResNet50^37^, (3) ResNet152V2^37^, (4) InceptionResNetV2^38^, (5) InceptionV3^39^, (6) Dense201^40^, and (7) Xception^41^ where transfer learning is used. For a meaningful comparison, we used the same classifier with all networks as the classifier shown in Fig. 2.

In addition, the performance was compared to that of the best-performing development networks: (1) XB5L3, (2) XB4L3, and (3) XB3L3.

### Comparison with ophthalmologists

In addition, a masked test was conducted by three ophthalmologists (T.K., A.T., S.B.) for comparison with DeepOpacityNet. A subset of 100 CFPs was selected randomly from the test set. The 100 CFPs were randomly chosen to contain 50 non-cataractous CFPs and 50 cataractous CFPs (i.e., balanced). The ophthalmologists were not aware of the distribution of the subset and were instructed to give one of two grades for each CFP: cataract present or absent. The performance of DeepOpacityNet on the same subset of 100 CFPs was compared to that of the ophthalmologists, according to the same performance metrics, as well as Fleiss kappa to measure the agreement between the ophthalmologists. It should be considered that this grading task is not routinely performed by ophthalmologists due to its difficulty. However, this aspect of the study is an interesting additional facet of the study.

### Visualization and error analysis of DeepOpacityNet

Guided Gradient-weighted Class Activation Mapping (guided Grad-CAMs)^42,43^ were used to visualize the features learned by DeepOpacityNet. This was performed for each CFP in the random test subset for the target class, as determined by the ground truth where the positive class (i.e., cataract present) and the negative class (i.e., cataract absent) were considered separately.

### Reporting summary

Further information on research design is available in the Nature Portfolio Reporting Summary linked to this article.

## Results

### The results of the preprocessing comparison

The results of the preprocessing comparison are shown in Supplementary Fig. 3. The results showed that using the full fundus region and CLAHE method led to the best results. Therefore, all the following results are reported using these optimum settings.

### The results of the development networks comparison

The results of the development networks performance comparison are summarized in Supplementary Figs. 4 and 5. They suggest that using networks with 3 layers (i.e., XB*n*L3) and ELU activation function achieved the best results. More detailed comparisons are summarized in Supplementary Tables 4 and 5 and Supplementary Fig. 6 for the XB*n*L3 networks. The results suggested that XB2L3 (i.e., DeepOpacityNet) achieved reasonable results while having the least number of blocks, which is important for having interpretable visualizations.

### Macro-average performance of DeepOpacityNet

**Fig. 3:**
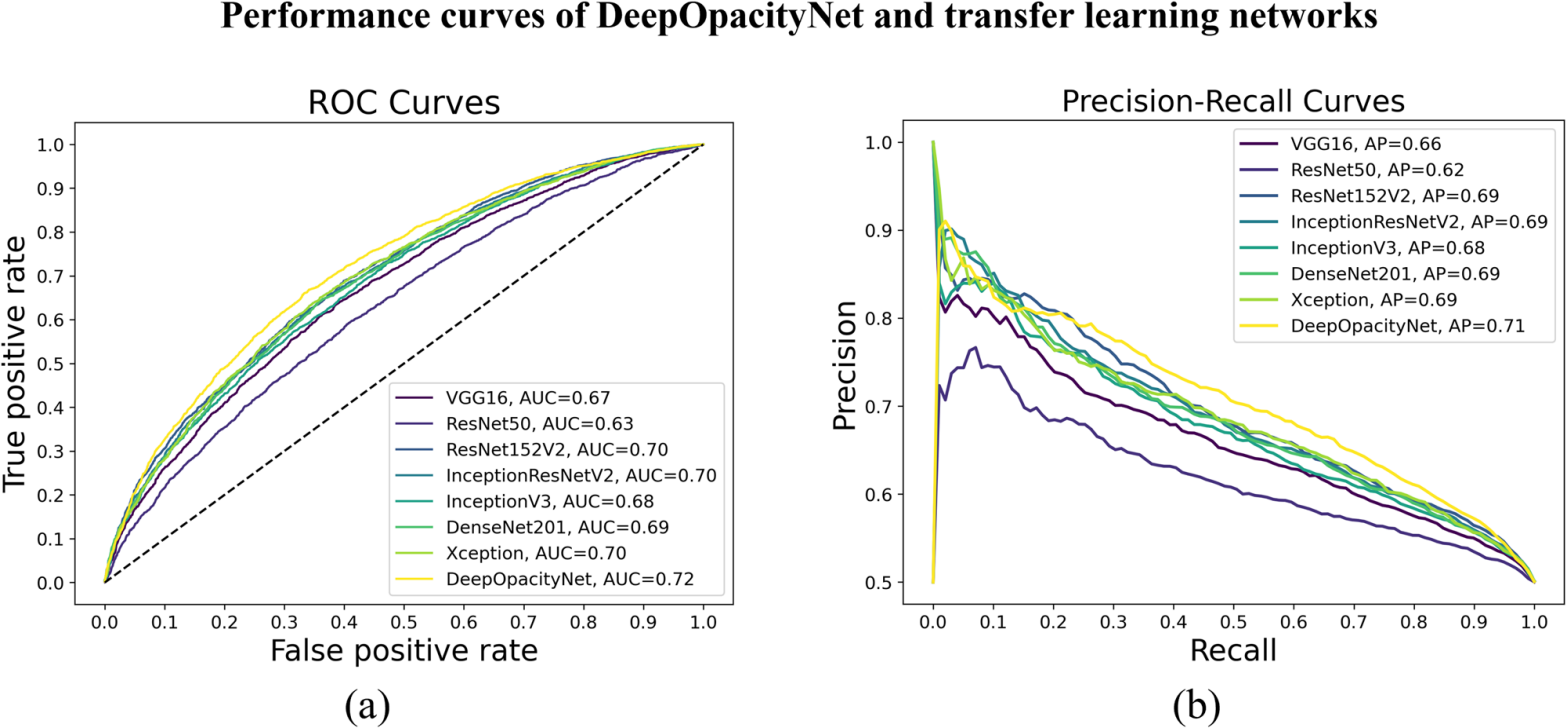
Performance curves of DeepOpacityNet and transfer-learning networks. **a** The macro-average receiver operating characteristic (ROC) and **b** precision-recall curves for DeepOpacityNet and the baseline networks on the test dataset.

The performance metrics of DeepOpacityNet and the baseline networks in correctly detecting cataracts from CFPs are summarized in Table 1. On the test set, DeepOpacityNet achieved the best macro-average metrics on accuracy, precision, recall, F1 score, *κ*, AUC, and AP with scores of 0.66 (95% CI: 0.64–0.68), 0.66 (95% CI: 0.65–0.69), 0.66 (95% CI: 0.64–0.69), 0.66 (95% CI: 0.64–0.69), 0.32 (95% CI: 0.29–0.35), 0.72 (95% CI: 0.70–0.74), and 0.71 (95% CI: 0.69–0.72), respectively. Figure 3 shows the macro-average ROC and precision-recall curves for all transfer-learning networks and DeepOpacityNet, where DeepOpacityNet achieved the best AUC of 0.72 and AP of 0.71. The performance comparison of DeepOpacityNet against the best-performing development networks is shown in Supplementary Table 4 and Supplementary Fig. 6.

**Table 1:** The macro-average performance metrics along with 95% confidence intervals for DeepOpacityNet and other methods on the testing dataset.

Network	Accuracy	Precision	Recall	F1 score	*κ*	AUC	AP
VGG16	0.63 (0.61, 0.64)	0.63 (0.61, 0.64)	0.63 (0.61, 0.64)	0.63 (0.61, 0.64)	0.25 (0.22, 0.29)	0.67 (0.65, 0.69)	0.66 (0.64, 0.68)
ResNet50	0.59 (0.58, 0.61)	0.59 (0.58, 0.61)	0.59 (0.58, 0.61)	0.59 (0.58, 0.61)	0.18 (0.15, 0.22)	0.63 (0.61, 0.65)	0.62 (0.60, 0.64)
ResNet152V2	0.63 (0.62, 0.65)	0.64 (0.62, 0.65)	0.63 (0.62, 0.65)	0.63 (0.61, 0.65)	0.26 (0.23, 0.30)	0.69 (0.68, 0.71)	0.69 (0.67, 0.71)
InceptionResNetV2	0.65 (0.64, 0.67)	0.65 (0.64, 0.67)	0.65 (0.64, 0.67)	0.65 (0.64, 0.67)	0.31 (0.27, 0.34)	0.71 (0.70, 0.73)	0.71 (0.69, 0.72)
InceptionV3	0.63 (0.61, 0.64)	0.63 (0.61, 0.64)	0.63 (0.61, 0.64)	0.63 (0.61, 0.64)	0.25 (0.22, 0.28)	0.68 (0.67, 0.7)	0.68 (0.66, 0.70)
Dense201	0.64 (0.63, 0.66)	0.64 (0.63, 0.66)	0.64 (0.63, 0.66)	0.64 (0.63, 0.66)	0.28 (0.25, 0.32)	0.69 (0.67, 0.71)	0.69 (0.67, 0.70)
Xception	0.64 (0.63, 0.66)	0.64 (0.63, 0.66)	0.64 (0.63, 0.66)	0.64 (0.63, 0.66)	0.29 (0.26, 0.32)	0.70 (0.68, 0.72)	0.69 (0.67, 0.71)
DeepOpacityNet	**0.66 (0.64**, **0.68)**	**0.66 (0.65**, **0.68)**	**0.66 (0.64**, **0.68)**	**0.66 (0.64**, **0.68)**	**0.32 (0.29**, **0.35)**	**0.72 (0.70**, **0.74)**	**0.71 (0.69**, **0.72)**

*AUC* area under curve, *AP* average precision, and bold font denote the highest scores. For all performance metrics, *N* = 1000 bootstrapping iteration with a fixed seed.

The results of macro-average categorical and binary AUC scores are summarized in Table 2. DeepOpacityNet achieved the best overall AUC score of 0.72 on binary labels of cataracts. Categorically, DeepOpacityNet achieved the best AUC for NS (i.e., the majority cataract type). However, it should be noted that all networks were trained using binary labels of cataract (i.e., present or absent). The results of macro-average categorical and binary AUC scores for the best-performing development networks are summarized in Supplementary Table 5.

**Table 2:** Macro-average categorical and binary area under curve (AUC) values of the DeepOpacityNet and the baseline networks on the test dataset.

Network	Binary	Categorical		
	CAT	CLO	PSC	NS
VGG16	0.67	0.68	0.58	0.67
ResNet50	0.63	0.63	0.55	0.64
ResNet152V2	0.70	0.66	**0.63**	0.70
InceptionResNetV2	0.71	**0.69**	**0.63**	0.71
InceptionV3	0.68	0.68	0.59	0.68
Dense201	0.69	0.68	0.60	0.69
EfficientNetB6	0.64	0.66	0.55	0.64
Xception	0.70	0.68	0.61	0.70
DeepOpacityNet	**0.72**	0.68	0.61	**0.72**

*CLO* cortical cataract, *PSC* posterior post-capsular cataract, *NS* nuclear cataract, bold font denotes the highest scores.

### Performance of DeepOpacityNet in comparison with ophthalmologists

The performance metrics of DeepOpacityNet and the three ophthalmologists on the 100 CFPs are summarized in Table 3. DeepOpacityNet achieved the best macro-average scores of 0.75, 0.75, 0.75, 0.75, 0.50, 0.84, and 0.85 on the accuracy, precision, recall, F1 score, *κ*, AUC, and AP, respectively, compared to 0.67, 0.67, 0.67, 0.67, and 0.34 on the accuracy, precision, recall, F1 score, and *κ* of the ophthalmologist with the highest performance. From the kappa test results, DeepOpacityNet had the highest agreement with the ground truth labels, compared to that of the ophthalmologists. The ROC and precision-recall curves for DeepOpacityNet, as well as the ophthalmologists’ performance, are shown in Supplementary Fig. 7, where they show the superior performance of DeepOpacityNet compared to the ophthalmologists.

**Table 3:** The performance metrics of DeepOpacityNet against the ophthalmologists for the masked test.

	Accuracy	Precision	Recall	F1 score	*κ*	AUC	AP
Ophthalmologist 1	0.67	0.67	0.67	0.67	0.34	NA	NA
Ophthalmologist 2	0.63	0.63	0.63	0.63	0.26	NA	NA
Ophthalmologist 3	0.62	0.63	0.62	0.61	0.24	NA	NA
DeepOpacityNet	**0.75**	**0.75**	**0.75**	**0.75**	**0.50**	**0.84**	**0.85**

Bold font denotes the highest scores.

The scores of Cohen’s kappa test for the ophthalmologists are summarized in Table 3 and show low agreement with the ground truth labels. The agreement between ophthalmologists and the pair-wise Cohen’s kappa test is shown in Supplementary Fig. 8. The *κ* scores were 0.49, 0.65, and 0.47 between the first and second, the first and third, and the second and third ophthalmologists, respectively. In addition, the Fleiss kappa test between the three ophthalmologists showed agreement of 0.01.

### Performance of DeepOpacityNet on the external datasets

The results of the external validation on the SiMES, SCES, and SINDI datasets are summarized in Table 4. DeepOpacityNet achieved higher performance metrics on the external datasets, compared to the development test set. DeepOpacityNet outperformed other networks on the accuracy, precision, AUC, and AP where it achieved 0.83, 0.75, 0.86, and 0.80 on the SiMES dataset, 0.88, 0.82, 0.89, 0.81 on the SCES dataset, and 0.88, 0.79, 0.88, 0.78 on SINDI dataset. DeepOpacityNet achieved the best F1 score and *κ* on SiMES and SCES datasets. A comparison of DeepOpacityNet with the development networks on the external datasets is summarized in Supplementary Table 6.

**Table 4:** The macro-average performance metrics of DeepOpacityNet and other networks on the external validation datasets.

Dataset	Size	Network	Accuracy	Precision	Recall	F1 score	*κ*	AUC	AP
SiMES Dataset	5752 CFPs	VGG16	0.78	0.71	0.75	0.72	0.44	0.84	0.76
		ResNet50	0.74	0.70	**0.78**	0.70	0.43	0.86	0.79
		ResNet152V2	0.77	0.70	0.75	0.71	0.43	0.83	0.77
		InceptionResNetV2	0.74	0.67	0.73	0.68	0.38	0.80	0.74
		InceptionV3	0.77	0.70	0.74	0.71	0.42	0.82	0.76
		Dense201	0.80	0.70	0.70	0.70	0.40	0.80	0.75
		Xception	0.80	0.71	0.68	0.69	0.39	0.81	0.76
		DeepOpacityNet	**0.83**	**0.75**	0.71	**0.73**	**0.46**	**0.86**	**0.80**
SCES Dataset	5745 CFPs	VGG16	0.74	0.65	0.77	0.66	0.35	0.85	0.77
		ResNet50	0.75	0.67	**0.80**	0.68	0.39	0.88	0.80
		ResNet152V2	0.83	0.69	0.71	0.70	0.40	0.81	0.73
		InceptionResNetV2	0.78	0.66	0.74	0.68	0.37	0.80	0.72
		InceptionV3	0.82	0.68	0.73	0.70	0.40	0.82	0.74
		Dense201	0.67	0.62	0.71	0.59	0.25	0.80	0.72
		Xception	0.80	0.67	0.73	0.69	0.38	0.81	0.74
		DeepOpacityNet	**0.88**	**0.82**	0.69	**0.73**	**0.46**	**0.89**	**0.81**
SINDI Dataset	5591 CFPs	VGG16	0.82	0.69	0.77	**0.71**	0.43	0.85	0.76
		ResNet50	0.80	0.69	**0.81**	0.71	**0.44**	0.88	0.78
		ResNet152V2	0.87	0.73	0.66	0.69	0.38	0.83	0.73
		InceptionResNetV2	0.86	0.71	0.68	0.70	0.39	0.82	0.73
		InceptionV3	0.87	0.73	0.69	0.71	0.42	0.83	0.74
		Dense201	0.80	0.66	0.73	0.68	0.36	0.82	0.73
		Xception	0.86	0.72	0.69	0.70	0.41	0.81	0.73
		DeepOpacityNet	**0.88**	**0.79**	0.67	0.71	0.42	**0.88**	**0.78**

Bold font denotes the highest scores before rounding.

### Visualizations of DeepOpacityNet

**Fig. 4:**
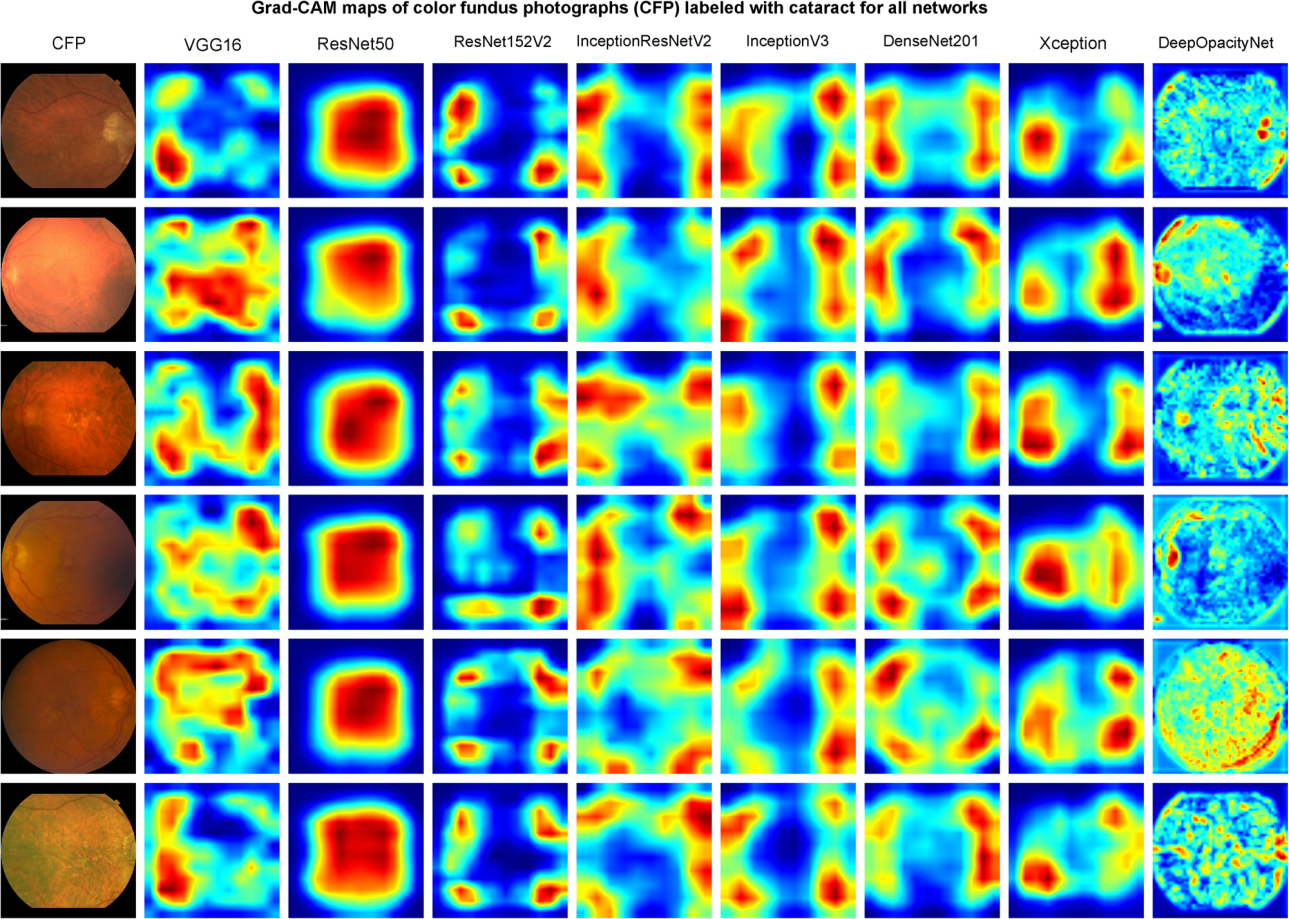
Grad-CAM maps of color fundus photographs (CFPs) labeled with cataracts for all networks The first column shows examples of CFPs, and each column shows the corresponding Grad-CAM maps obtained from VGG16, ResNet50, ResNet152V2, InceptionResNetV2, InceptionV3, DenseNet201, Xception, and DeepOpacityNet respectively (the figure is better visualized enlarged).

**Fig. 5:**
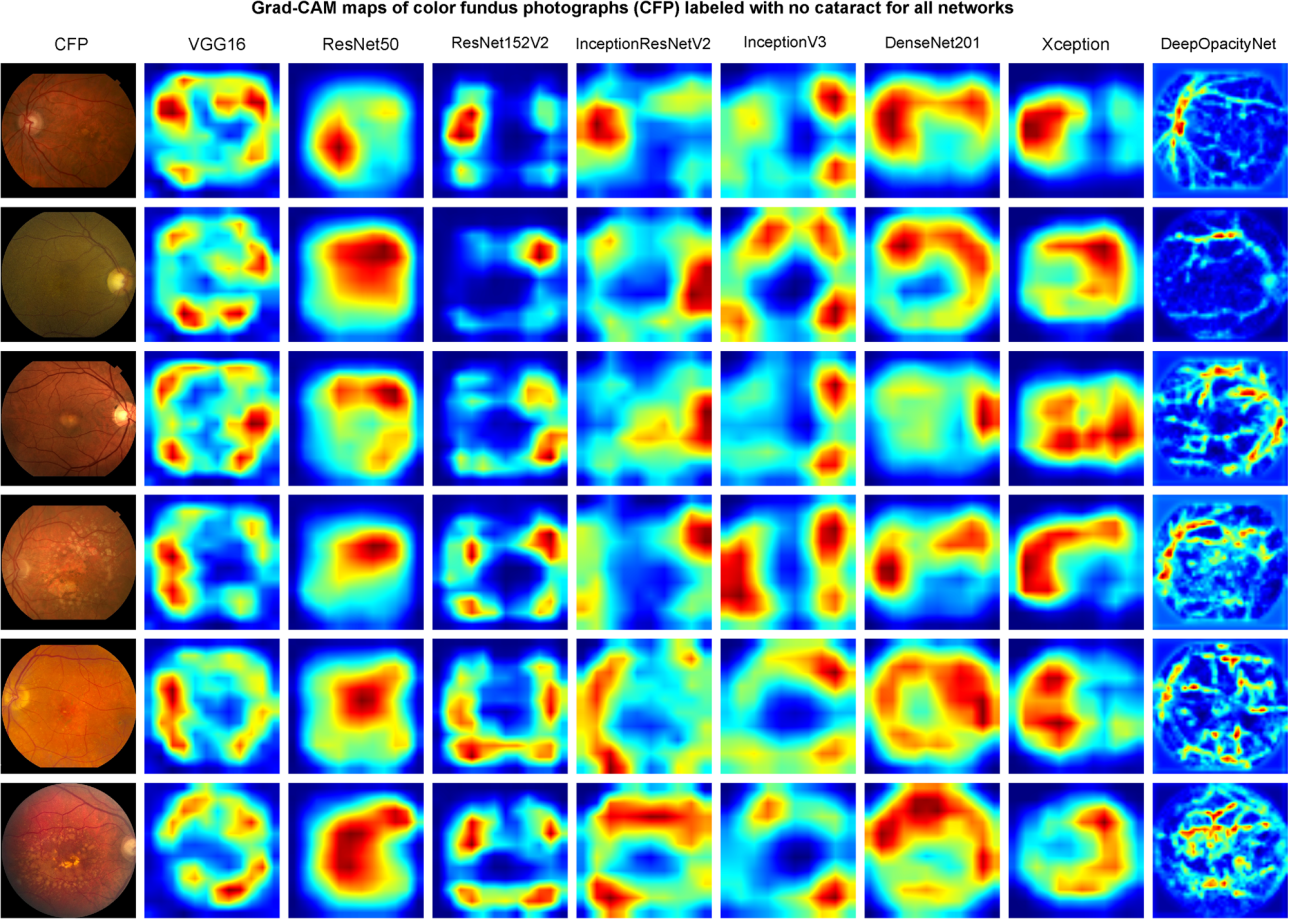
Grad-CAM maps of color fundus photographs (CFPs) labeled with no cataract for all networks. The first column shows examples of CFPs, and each column shows the corresponding Grad-CAM maps obtained from VGG16, ResNet50, ResNet152V2, InceptionResNetV2, InceptionV3, DenseNet201, Xception, and DeepOpacityNet, respectively (the figure is better visualized enlarged).

Examples of the activation maps obtained using guided Grad-CAM for DeepOpacityNet and the baseline networks are shown in Fig. 4 for CFPs labeled with cataract (i.e., true positive) and Fig. 5 for some challenging CFPs labeled with no cataract (i.e., true negative) where the CFPs were dark or blurry. The visualization of the same examples is shown in Supplementary Figs. 9 and 10 for the best-performing development networks.

From the guided Grad-CAM heatmaps in Fig. 4, DeepOpacityNet shows high signal at blurred areas that are close to edges (e.g., the periphery, optic disc rim, choroidal blood vessels, and around large vessels), presumably because blurriness at these feature edges is associated with increased likelihood of cataract. Also, it shows a low signal for areas that contain retinal blood vessels, presumably because the higher the visibility of the retinal blood vessels, the less likely significant cataract presence may be. VGG16 shows a high signal at blurry areas around the optic disc or blood vessels, which agrees with DeepOpacityNet, but VGG16 tends to highlight larger areas (i.e., more spread) compared to DeepOpacityNet. ResNet50 shows a high signal in the central area. However, ResNet, as shown in Supplementary Data 1, seems to have similar heatmaps in almost all CFPs labeled with cataracts, which is not very informative about the features highlighted by ResNet. InceptionResNetV2, InceptionV3, DenseNet201, and Xception show a high signal at peripheral blurry areas including the optic disc or close to it. Sometimes these areas contain blood vessels, which may be counterintuitive. ResNet152V2 shows similar heatmaps to InceptionResNetV2, InceptionV3, DenseNet201, and Xception, but the highlighted areas are less spread areas.

From the guided Grad-CAM heatmaps in Fig. 5, DeepOpacityNet has high signal in areas that contain retinal large and small blood vessels and the optic nerve head, which is intuitive because higher visibility of blood vessels is associated with a lower likelihood of cataracts. VGG16 and DenseNet201 have high signals in areas that contain or close retinal blood vessels, but the areas are more spread compared to DeepOpacityNet. ResNet50 has high signal in large central areas that contain small blood vessels or the optic disc, but not all the blood vessel regions are highlighted. Also, the highlighted areas are not easy to interpret because they are highly spread. ResNet152V2 and InceptionV3 have high signals in peripheral areas that contain large blood vessels or close to the optic disc, but not all the blood vessel regions are highlighted. Also, InceptionV3 highlights areas that are more spread. InceptionResNetV2 and Xception have high signals in areas of the optic disk and the blood vessels around it. However, these areas do not contain all blood vessel areas. Therefore, DeepOpacityNet clearly highlights the blood vessels as an important feature of cataract absence, and the VGG16 network seemed to have more interpretable heatmaps as compared to other transfer-learning networks.

Overall, the visualizations suggested that the pixels along well-visualized retinal blood vessels were usually features of cataract absence, according to DeepOpacityNet. By contrast, the characteristics of other image areas (particularly those overlying large choroidal vessels, featureless macular areas, or general haze) were usually features of cataract presence. AMD features and the optic disc rim were sometimes less likely and sometimes more likely to be features of cataract presence, presumably according to the crispness of the edges.

## Discussion

### Main findings and interpretation

On the full test set, DeepOpacityNet achieved superior accuracy, AUC, and AP to those of the baseline networks (see Table 1 and Fig. 3) in the detection of cataracts from CFPs. Also, based on the *κ* score, DeepOpacityNet achieved the highest level of agreement with the ground truth labels.

On the test subset, DeepOpacityNet achieved superior performance to that of three ophthalmologists (see Table 2). Also, there was very low agreement between the three ophthalmologists; this variability between their gradings likely reflects the very difficult nature of the task for humans, since CFPs are typically not considered amenable to human grading for cataract presence. This also suggests that DeepOpacityNet can be of great utility in detecting cataracts from CFPs with more accuracy and consistency.

On external validation, DeepOpacityNet achieved superior performance on all performance metrics, compared to its performance on the main test set. It outperformed all transfer-learning networks on the three external datasets, where it achieved the best accuracy, precision, AUC, and AP. DeepOpacityNet achieved the best F1 score and *κ* on the SiMES and SCES datasets, while ResNet50 achieved the best recall on all three external datasets. This could be because ResNet50 had higher false positive rates, where it did not perform well in differentiating between non-cataract opacities and cataract opacities. This is reflected in the accuracy and precision scores for this network. This may confirm our hypothesis that AREDS2 is a challenging dataset, so DeepOpacityNet was able to perform with high accuracy on these external datasets, which have non-cataract opacities. Also, it should be noted that the development dataset has different ethnicities and a higher prevalence of retinal diseases, compared to the external datasets (i.e., AREDS2 (predominantly white), SiMES (Malays), SCES (Chinese), and SINDI (Indians), which suggests that DeepOpacityNet could generalize well.

On the visualizations, the heatmaps obtained by guided Grad-CAMs showed that DeepOpacityNet tends to detect small blurry areas at the macula, the optic nerve, and around blood vessels as features for cataract presence (see Fig. 4) and retinal blood vessels as features for cataract absence (see Fig. 5). The analysis done by one of the ophthalmologists (T.K.) suggests that retinal blood vessels were more likely to be features of cataract absence (i.e., because the higher their visibility, the less likely cataract is present), while other macular areas (including areas overlying large choroidal vessels) were more likely to be features of cataract presence. Compared to the study done by Tham et al.^44^, where the opacification in visually significant cataracts was visualized, we attempted to explore other features that may be relevant to cataract presence in a challenging dataset such as the AREDS2 dataset.

### Clinical importance

The diagnosis of age-related cataracts typically requires in-person assessment by a trained ophthalmologist. This represents a challenge in many developing countries, where there are few ophthalmologists, such that the costs and logistics of in-person assessment may pose substantial barriers. In addition, even in many high/middle-income countries, many screening programs are based on CFP taken by technicians or nurses, without an ophthalmologist present. These may represent important missed opportunities to detect cataracts. Examples include DR and glaucoma screening in many countries. Even outside DR and glaucoma screening, large organizations such as the veterans affairs (VA) increasingly provide services in this way. For example, the VA launched its technology-based eye care services (TECS) in some regions in 2014. In this approach, a trained ophthalmology technician stationed in a primary care clinic performs protocol-determined tests including visual acuity assessment and CFP^45,46^. The information is interpreted remotely and patients with possible abnormal findings are scheduled for an in-person examination by an ophthalmologist in the central eye clinic. At present, cataracts cannot be diagnosed reliably by human graders in this setting, but this would be possible if automated approaches were able to detect cataracts from CFP. CFPs are of great interest because the opacity of the crystalline lens due to cataracts appears as different degrees of blurriness in the retinal structures of CFPs. Therefore, the existence of deep-learning methods that can detect cataracts from CFPs with high accuracy and consistency could be of great importance to make cataract screening more accessible.

According to many studies in the literature that used population-based datasets^47–58^, cataracts can be classified from CFPs into four grades, according to the level of opacity and the perceived details of the retinal structures: none, mild, moderate, and severe. In the non-cataract grade, all the retinal structures can be seen clearly, including large and small blood vessels and the optic disc. In the mild grade, the small vessels can hardly be seen but the other structures can. In the moderate grade, only the large vessels and optic disc can be seen. In the severe grade, the large vessels and optic disc can be seen either hardly or not at all. However, these visual distinctions are not clear on a dataset such as the AREDS2 dataset, which makes the detection task harder even for an ophthalmologist (see Supplementary Figs. 11 and  12). Moreover, these studies provided no visualizations of their methods, to show these visual distinctions. Therefore, visualizing the deep network can help in understanding the salient features in CFPs that are relevant to cataracts.

### Comparison with literature

Several studies in the literature have used machine learning and deep-learning methods to detect and grade NS cataracts from CFPs (see Supplementary Table 7)^47–62^. Most of these studies used small population-based datasets from a single center (see Supplementary Table 8) and reported only the performance of their methods, i.e., without comparison with the performance of ophthalmologists on the same test set. In addition, they did not attempt to explain or interpret how their methods worked. There is general agreement in the literature that the severity of cataracts is associated with the degree of blurriness in CFPs. However, in a large multi-center dataset such as the AREDS2 dataset, the participants were generally elderly, so there was a high chance of corneal or ocular surface abnormalities that might simulate cataracts. Also, in a multi-center setting, the characteristics of a particular camera used at a specific center would differ from that of another center. In addition, in the AREDS2, all types of cataracts were included; this is an important strength compared to most previous studies, which considered NS only, since CLO and PSC cataracts may induce different changes to CFPs from those caused by NS.

In this study, we proposed DeepOpacityNet, which was trained and evaluated on a large multi-center dataset obtained and curated from the AREDS2. The network was designed using separable convolutions to limit the network capacity and residual connections to enhance the gradient flow. DeepOpacityNet had more convolutional layers for each residual block and fewer pooling operations, which resulted in a large spatial size at the final convolutional layer that helped the network to capture more fine features (see visualizations in Figs. 4 and  5), hence enhancing performance (see Table 1). Also, ELU was used for the activation function, which enhanced network performance (see results in Table 1). DeepOpacityNet outperformed transfer learning using common networks such as VGG16, ResNet, ResNet152V2, InceptionResNetV2, InceptionV3, DenseNet201, and Xception, and its visualizations were more intuitive as discussed in the visualization results.

However, the observed performance metrics were lower than some that have been reported in the literature for other datasets. The likely reason is the nature of the AREDS2 dataset. In particular, the AREDS2 is a highly desirable dataset for training purposes and a highly challenging dataset for testing purposes. First, since the AREDS2 was a clinic-based study where the study population had a relatively high mean age and all participants had at least intermediate AMD, there was a high prevalence of ocular and systemic illnesses, including cataracts. This differs substantially from the situation with adult population-based datasets, where the mean age is typically lower and the prevalence of ocular and systemic illnesses, including cataracts, is also much lower. This means that, in the AREDS2 dataset, there was a high proportion of positive cataract cases; this is beneficial for training purposes and challenging for testing purposes. Indeed, unlike many other datasets, the AREDS2 dataset was almost balanced between positive and negative cases. This is ideal, since it forces the models to make meaningful predictions, rather than predictions that appear meaningful but are based mostly on bias towards the majority class. Second, the dataset also contained many cases close to the threshold for cataract presence/absence (i.e., just absent or just present). Again, this is ideal for training purposes and challenging for testing purposes. Third, the AREDS2 population likely had relatively common coexisting ocular conditions that might simulate the presence of cataracts in CFPs (e.g., dry eye, which is very common in the elderly population^63,64^). This is desirable for training purposes since it might help the models learn to distinguish blur in CFPs caused by genuine cataracts vs. other pathology, which is extremely difficult for human graders to perform. However, on the testing side, it likely leads to a higher false positive rate (by both the networks and human graders). Finally, the multi-center nature of the AREDS2 dataset is desirable for diversity and higher levels of generalizability, since the CFPs were collected at 82 different clinics across the US, i.e., using many different cameras and operators.

### Strengths, limitations, and future work

The strengths of this work include using a large multi-center dataset curated from the AREDS2, which represents an ideal dataset for training and testing for the reasons discussed above. The strengths include the detection of cataracts in a dataset that included diverse types and severity of cataracts in different combinations. All eyes were included, whether they had only one type of cataract (i.e., NS, CLO, or PSC alone), two types (e.g., both NS and CLO), or all three cataracts simultaneously. NS was the most common type in the dataset, which reflects the real-world distribution of cataract prevalence. Hence, the task demanded during training and testing was more complex than if only NS had been considered, with other cataract types or combinations excluded. In addition, visualizing networks to understand some features that are related to cataract presence or absence is another strength. The strengths include the generalizability of the developed models with high performance on the external datasets, which were from different ethnicities (i.e., Malays, Chinese, and Indians).

There are some limitations in this work. The deep-learning models were not trained to detect each cataract type separately (i.e., just detect the presence or absence of cataracts), owing to lower quantities of data in the CLO and PSC types. Also, the models were not trained to detect cataract severity, because the majority of the data were borderline cases, which is harder to classify. Moreover, using data from the aging population for training the model may reduce the generalizability of the model to other populations.

Potential future work includes attempting (1) to classify cataract types and severity grading using the AREDS2 dataset, (2) to add more datasets for training, to enhance the network generalizability further, (3) to visualize the distinctive features for each cataract type using the AREDS2 dataset, and (4) to visualize the distinctive features for each cataract grade using the AREDS2 dataset.

### Supplementary information

Supplementary Information

Description of Additional Supplementary Files

Supplementary Data 1

Supplementary Data 2

Supplementary Data 3

Supplementary Data 4

Supplementary Data 5

Supplementary Data 6

Reporting Summary

## Data Availability

The AREDS2 dataset containing the data analyzed and generated during the current study has been deposited in dbGAP, accession number phs002015.v1.p1, and are made freely available. The external datasets (SiMES, SCES, and SINDI) are not publicly available and any requests should be made to Dr. Cheng Ching Yu. The visualization of all networks in this study is included in the Supplementary Data 1. The ground truth labels and predictions of all models as well as the subjective grading of the ophthalmologists are included in Supplementary Data 2–6.

## Code availability

The models and scripts are available at https://github.com/ncbi/DeepOpacityNet (10.5281/zenodo.10127002)^65^.
