# Supplementary material for: A deep network DeepOpacityNet for detection of cataracts from color fundus photographs
Source: Commun Med (Lond). 2023 Dec 16;3:184. doi: 10.1038/s43856-023-00410-w (PMC10725427; doi:10.1038/s43856-023-00410-w)
Supplement: Supplementary file 1 — Supplementary Information [file 43856_2023_410_MOESM1_ESM.pdf]

Supplementary Figures

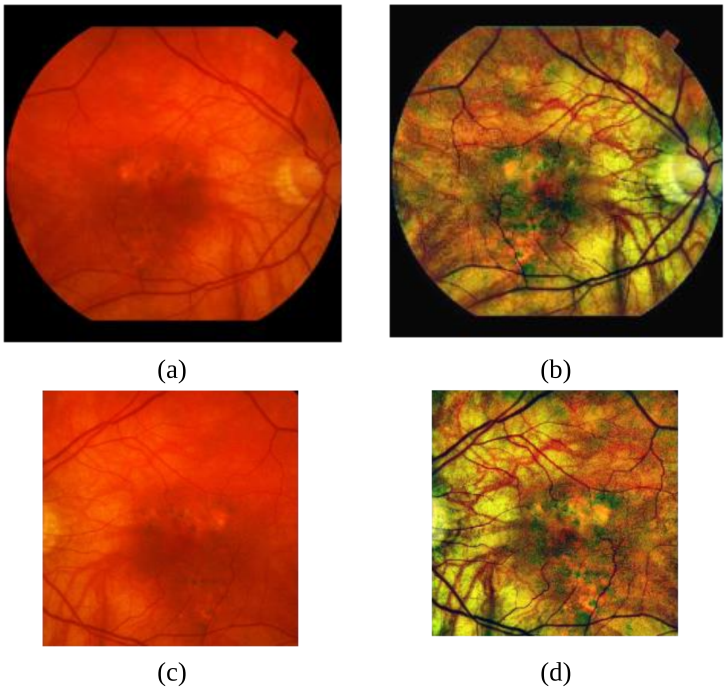

Supplementary Figure 1: Preprocessing of color fundus photo (CFP). Different preprocessing of CFP (a) is the original CFP, and (b) is the CFP after applying contrast limited adaptive histogram equalization (CLAHE) to each channel, (c) is the cropped CFP, and (d) is the cropped CFP after applying CLAHE to each channel.

.

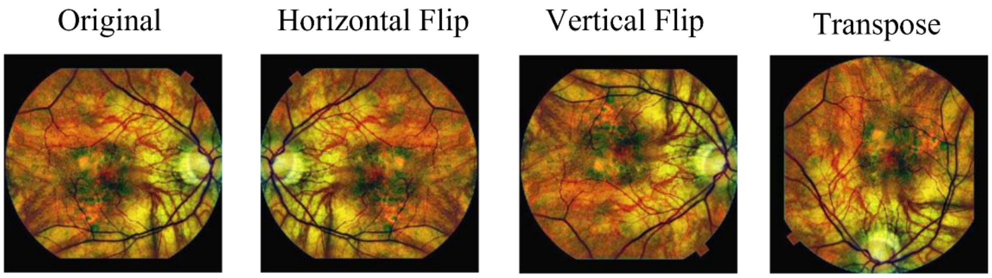

Supplementary Figure 2: Different augmentation methods. The augmentation methods that were applied to the input image, which included random horizontal flipping, random vertical flipping, and random transpose; each with probability of 0.5 to allow for combination of them.

.

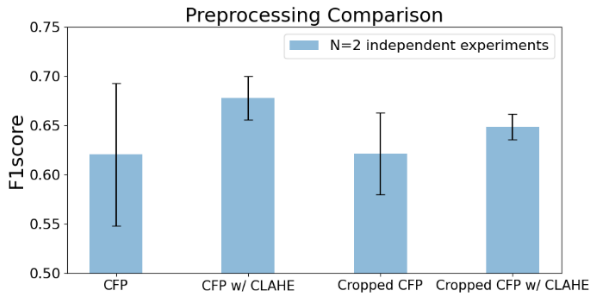

Supplementary Figure 3: Comparison of preprocessing methods. Bar plot of the area under curve (AUC) of some preliminary networks using different processing methods on the test dataset. Clearly, using CLAHE method enhanced the results. Also, using the full CFP is better than cropping.

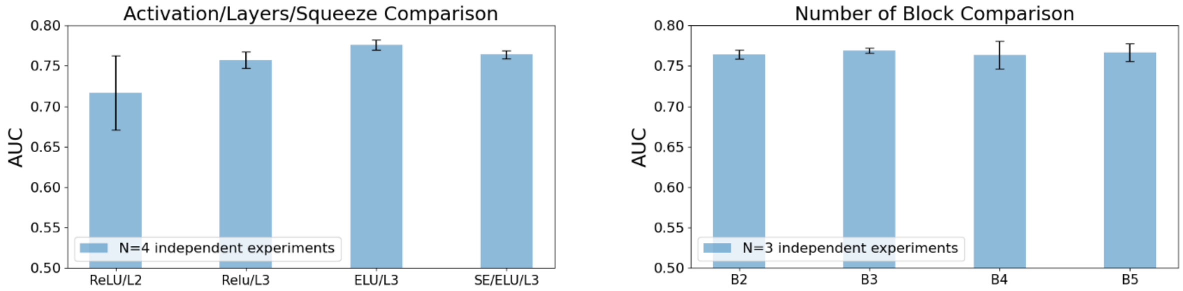

(a) (b)

Supplementary Figure 4: Comparison of different network settings on the validation set. The summary of AUC results for all developed networks on the validation set where the results are averaged over networks with B5, B4, B3, and B2 blocks (i.e., N=4) in (a) and averaged over the last three cases (i.e., N=3) in (b). From (a), using ELU/L3 achieved the best macro-average AUC of 0.78, which shows the efficacy of using ELU activation and 3 layers per block. From (b), There were marginal differences between all cases, and B3 slightly outperformed other cases with AUC of 0.77, but this suggest that the number of convolutional blocks can be reduced for sake of better visualization.

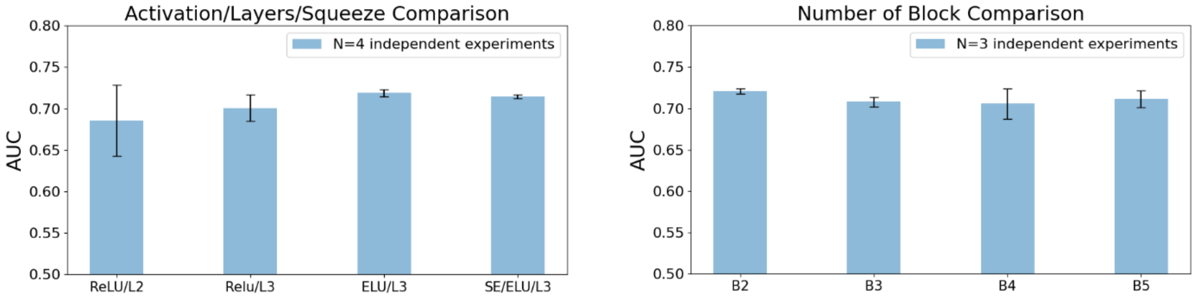

(a) (b)

Supplementary Figure 5: Comparison of different network settings on the test set. The summary of AUC results for all developed networks on the test set where the results are averaged over networks with B5, B4, B3, and B2 blocks in (a) and averaged over the last three cases in (b). From (a), using ELU/L3 achieved the best macro-average AUC of 0.72, which confines with the validation results. From (b), B2 achieved AUC of 0.72, compared to 0.71 for B3, B4 and B5.

Performance curves of the best-performing development networks

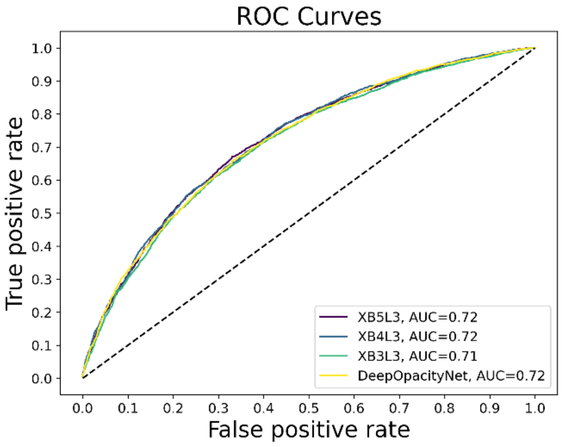

(a)

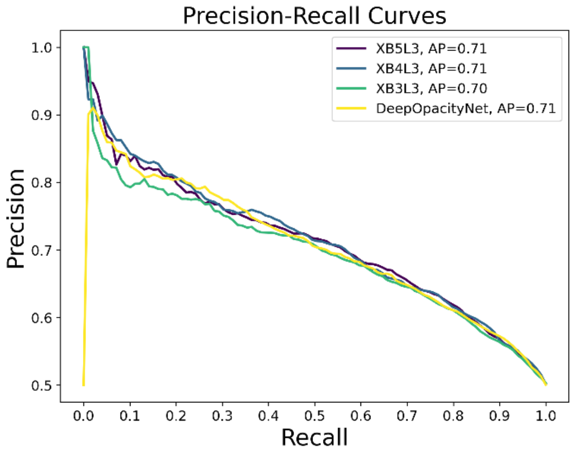

(b)

Supplementary Figure 6: Performance curves of the best-performing development networks. (a) the macro average receiver operating characteristic (ROC) curves for the best-performing development networks (with the area under the curve), and (b) the macro average precision-recall curves (with the average precision). All results are based on the test dataset.

Performance of DeepOpacityNet versus ophthalmologists on the subjective test set

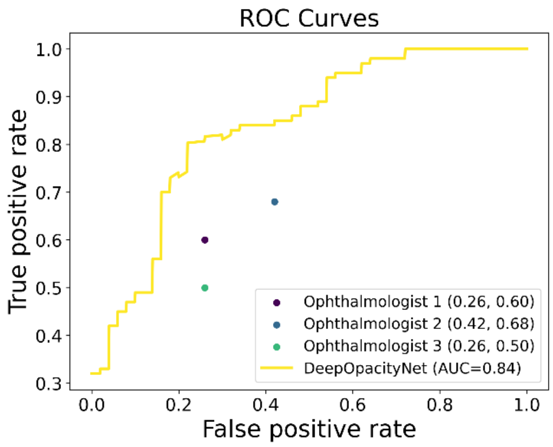

(a)

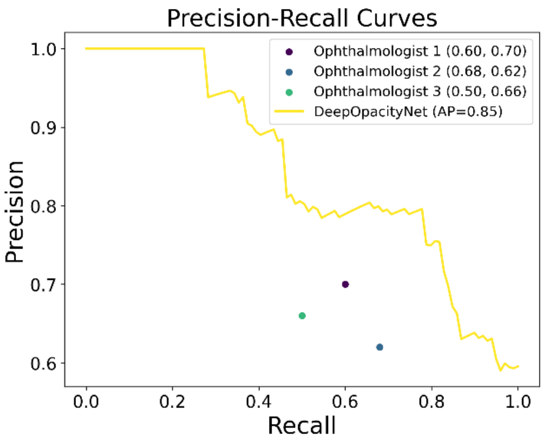

(b)

Supplementary Figure 7: Performance curves of DeepOpacityNet versus ophthalmologists on the subjective test set. (a) the macro average receiver operating characteristic (ROC) curve for DeepOpactiyNet (with the area under the curve) and the ophthalmologists' performance (false positive rate, true positive rate), and (b) the precision-recall curve for DeepOpacityNet (with the average precision) and the ophthalmologists' performance (recall, precision). All results are based on the random subset of the test dataset.

Agreement between subjective gradings of ophthalmologists on the test subset

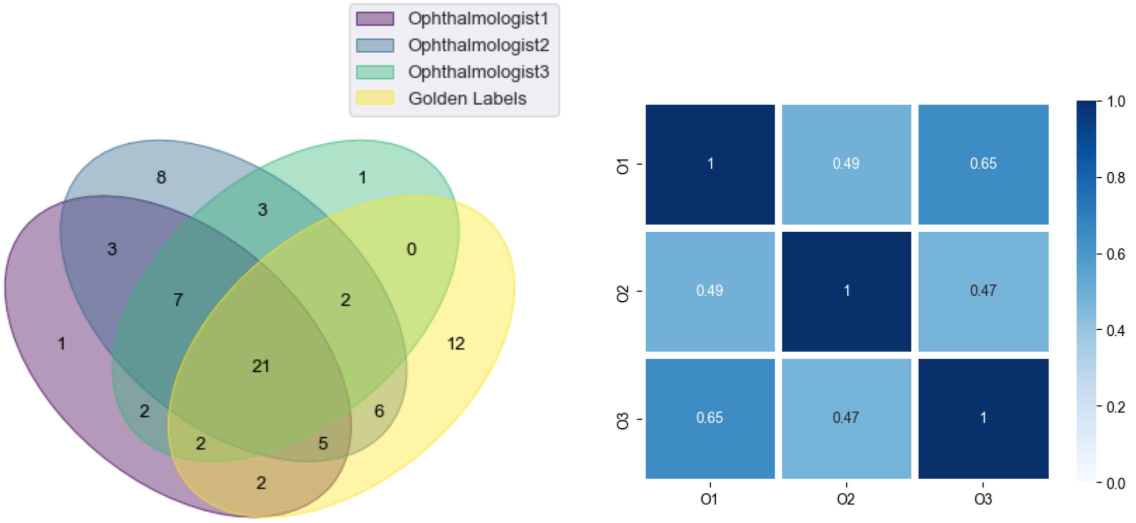

(a) (b)

Supplementary Figure 8: Agreement between subjective gradings of ophthalmologists on the test subset. (a) Venn diagrams that show the agreement on cataract grading between the ophthalmologists and the golden labels where each set contains 50 elements, and (b) the pair-wise overall agreement between the ophthalmologists using Cohen kappa test.

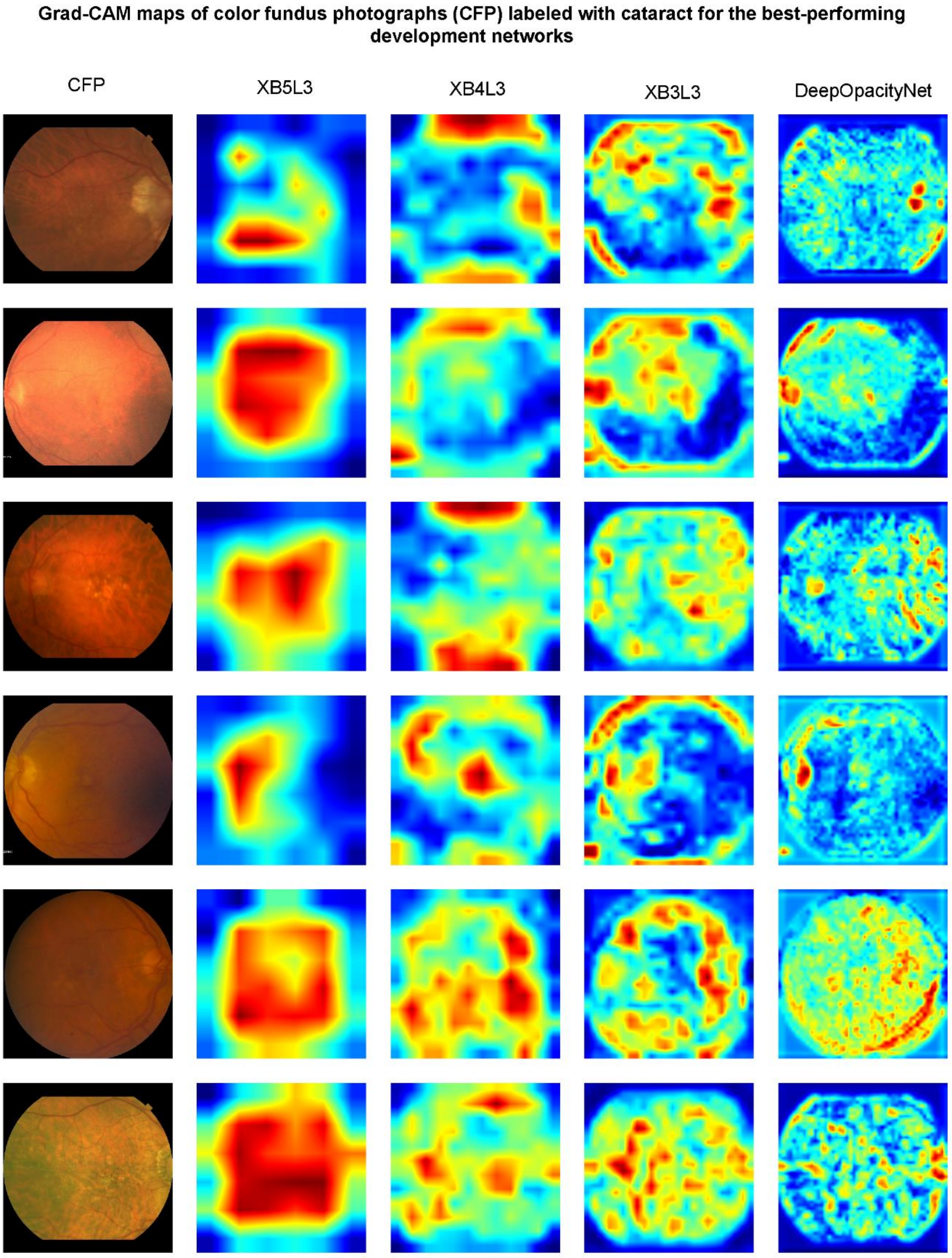

Supplementary Figure 9: Grad-CAM maps of color fundus photographs (CFPs) labelled with cataract for the best-performing development networks. The first column shows examples of CFPs, and each column shows the corresponding Grad-CAM maps obtained from XB5L3, XB4L3, XB3L3, and DeepOpacityNet respectively.

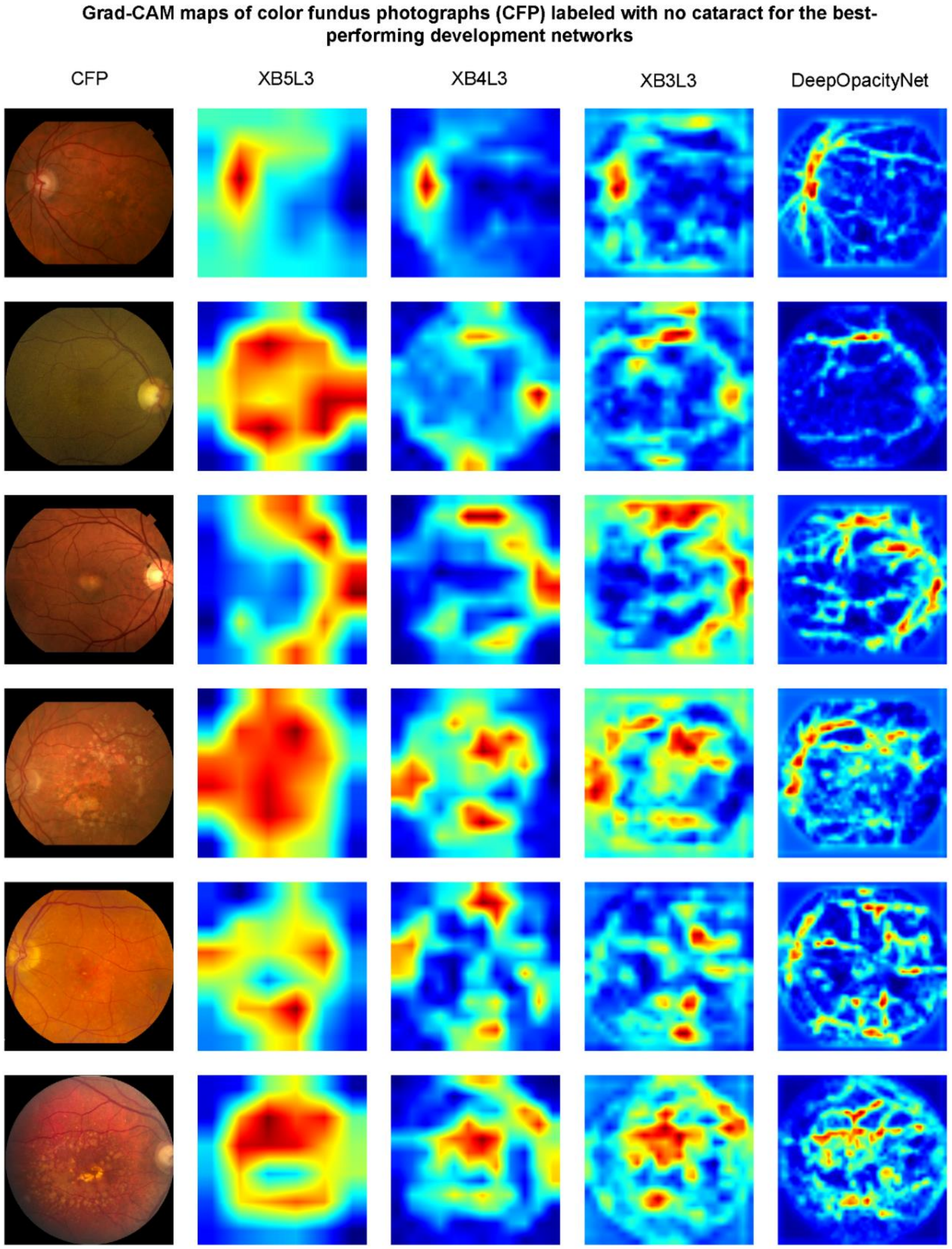

Supplementary Figure 10: Grad-CAM maps of color fundus photographs (CFPs) labelled with no cataract for the best-performing development networks. The first column shows examples of CFPs, and each column shows the corresponding Grad-CAM maps obtained from XB5L3, XB4L3, XB3L3, and DeepOpacityNet respectively.

Examples of different grades of cataract in the study dataset

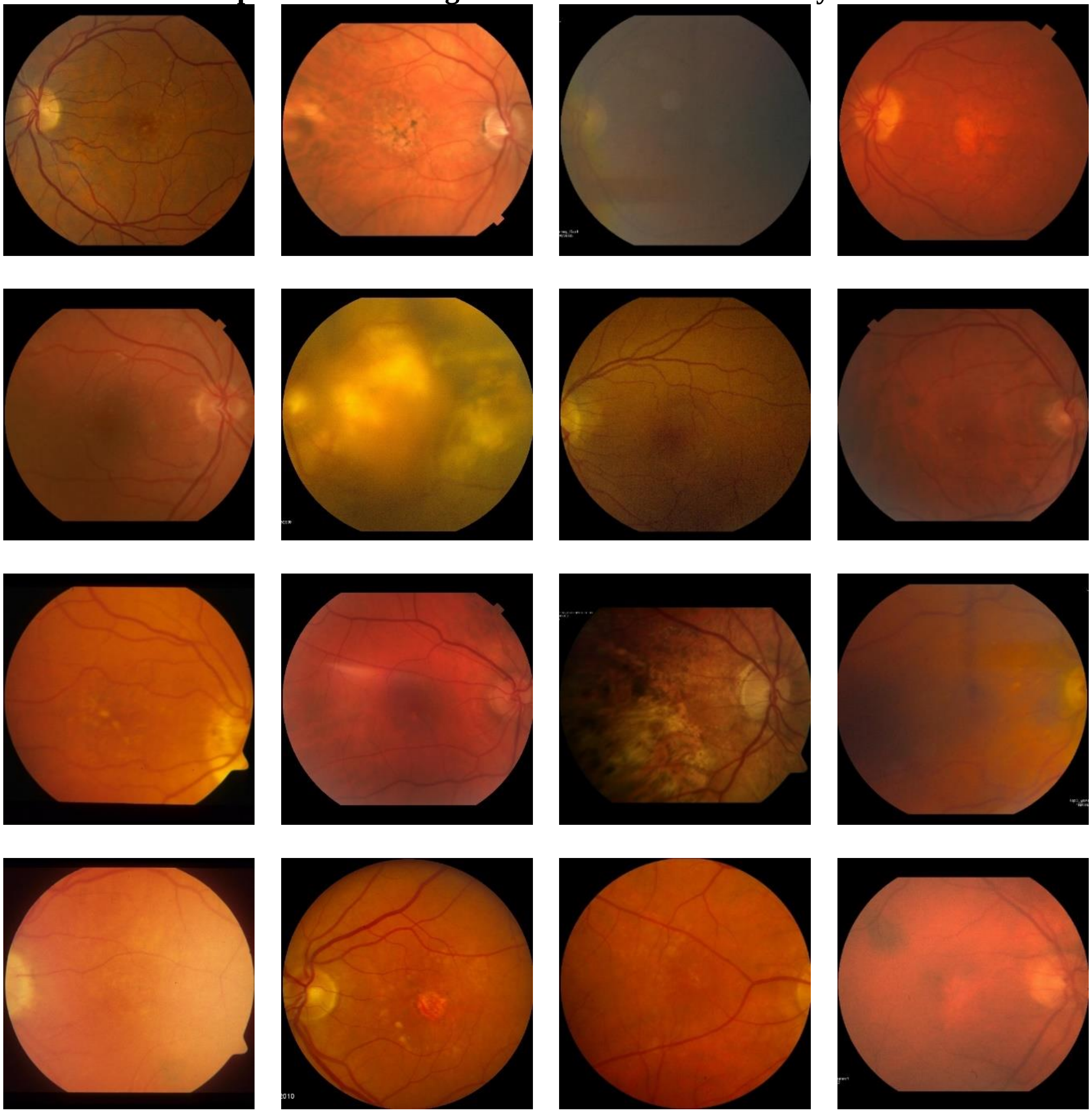

(a) (b) (c) (d)

Supplementary Figure 11: Examples of different grades of cataract in the Age-Related Eye Disease Study 2. The grades of the color fundus photos for columns (a)-(d) are (a) non-cataract, (b) mild, (c) moderate, and (d) severe.

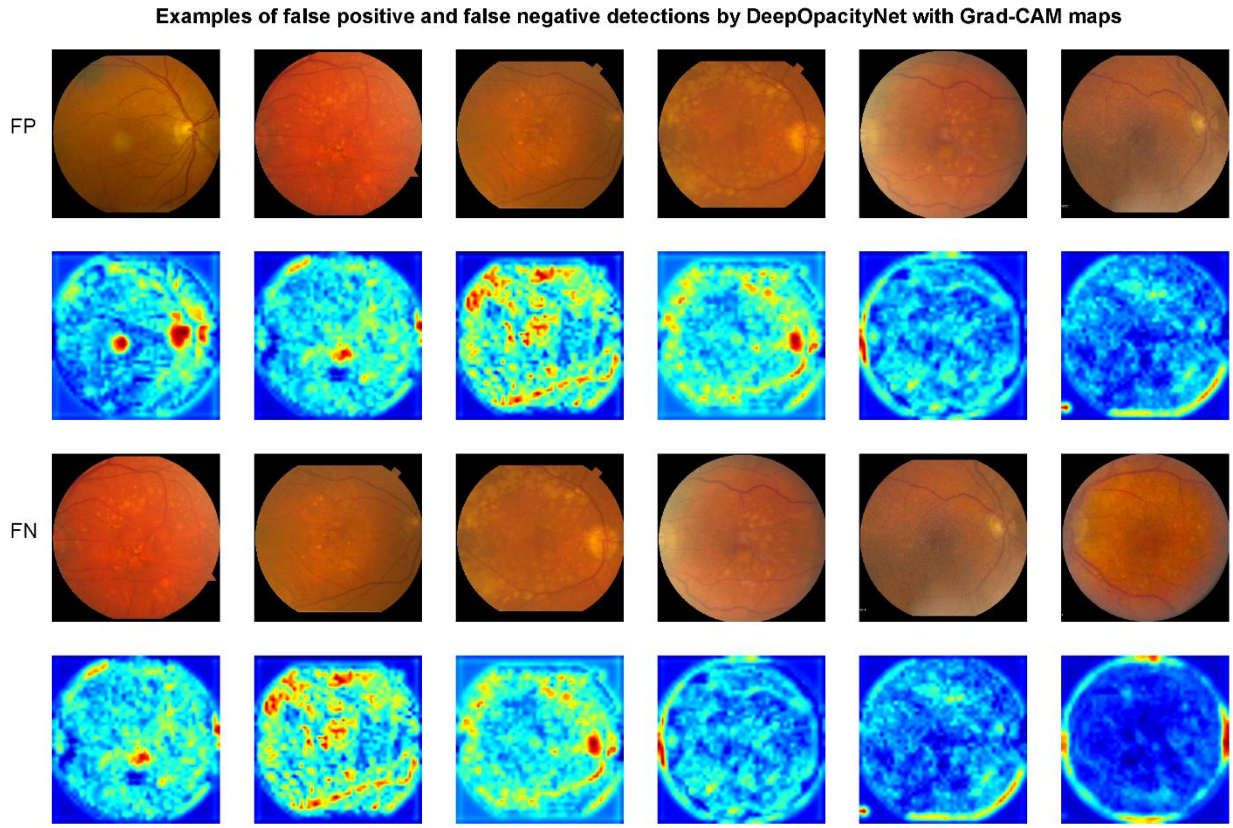

Supplementary Figure 12: Examples of false positive (FP) and false negative (FN) CFPs as detect by DeepOpacityNet. The negative examples have blurriness that resembles cataract that were highlighted by heatmaps. However, a potential reason could be that a degree of cataract was present, but just under the threshold definition of "cataract present".

Supplementary Tables

TABLE 1: The characteristics of the external datasets obtained from the Singapore Eye Research Institute.

Characteristic	SiMES	SCES	SINDI
Number of retinal images (field 2)	5,752	5,745	5,591
Number of participants	3,014	3,011	2,937
Mean (S.D.) age in years	58.4 (10.8%)	58.4 (9.2%)	56.1 (9.1%)
Number of Males (%)	1,452 (48.2%)	1,500 (49.8%)	1,493 (50.8%)
Number of eyes with clinically significant cataract (%)	1,271 (22.1%)	913 (15.9%)	821 (14.7%)

Supplementary Table 2: Architecture comparison of DeepOpacityNet and the development networks.

Network	No. residual blocks	No. conv layers	No. parameters	No. trainable parameters
XB5L2	5	12	11,831,810	11,807,810
XB5L3	5	17	17,474,306	17,442,370
XB4L2	4	10	3,122,690	3,110,978
XB4L3	4	14	4,542,210	4,526,658
XB3L2	3	8	865,282	859,714
XB3L3	3	11	1,221,890	1,214,530
XB2L2	2	6	260,866	258,370
**DeepOpacityNet**	**2**	**8**	**348,162**	**344,898**

Supplementary Table 3: Number of filters of DeepOpacityNet and the development networks.

Network	Init Conv. Block	Residual Blocks				
	0	1	2	3	4	5
XB5L2	{32, 64}	128^2	256^2	512^2	1024^2	2048^2
XB5L3	{32, 64}	128^3	256^3	512^3	1024^3	2048^3
XB4L2	{32, 64}	128^2	256^2	512^2	1024^2	-
XB4L3	{32, 64}	128^3	256^3	512^3	1024^3	-
XB3L2	{32, 64}	128^2	256^2	512^2	-	-
XB3L3	{32, 64}	128^3	256^3	512^3	-	-
XB2L2	{32, 64}	128^2	256^2	-	-	-
**DeepOpacityNet**	**{32, 64}**	$\mathbf{128^3}$	$\mathbf{256^3}$	**-**	**-**	**-**

Power represents no. of layers

Supplementary Table 4: The macro average performance metrics for DeepOpacityNet and the best-performing development networks on the testing dataset. Reducing the number of blocks did not greatly reduce the performance, but it helped to have large size of the final convolutional maps.

Metric	Accuracy	Precision	Recall	F1 score	κ	AUC	AP
XB5L3	**0.67**	**0. 67**	**0. 67**	**0. 67**	**0.33**	**0.72**	**0.71**
XB4L3	0.66	0.66	0.66	0.66	0.32	**0.72**	**0.71**
XB3L3	0.65	0.66	0.65	0.64	0.30	0.71	0.70
**DeepOpacityNet**	0.66	0.66	0.66	0.66	0.32	**0.72**	**0.71**

Supplementary Table 5: Macro average area under curve (AUC) scores of DeepOpactiyNet and the best-performing development networks for each cataract type on the test dataset

Network	Binary	Categorical		
	CAT	CLO	PSC	NS
XB5L3	**0.72**	0.67	**0.62**	**0.72**
XB4L3	**0.72**	**0.68**	0.60	**0.72**
XB3L3	0.71	0.66	0.61	0.71
**DeepOpacityNet**	**0.72**	**0.68**	0.61	**0.72**

TABLE 6: The macro average performance metrics for DeepOpacityNet and the best-performing development networks on the external datasets. The bold font highlights the highest scores.

Dataset	Network	Accuracy	Precision	Recall	F1 score	κ	AUC	AP
SiMES	XB5L3	0.76	0.71	**0.79**	0.72	0.46	0.86	0.79
	XB4L3	0.80	0.73	**0.79**	**0.74**	0.49	**0.87**	**0.81**
	XB3L3	0.82	0.74	0.73	**0.74**	0.47	0.86	0.79
	DeepOpacityNet	**0.83**	**0.75**	0.71	0.73	0.46	0.86	0.80
SCES	XB5L3	0.86	0.74	0.76	**0.75**	0.88	0.88	0.79
	XB4L3	0.79	0.69	**0.80**	0.70	0.43	0.88	**0.81**
	XB3L3	**0.88**	**0.83**	0.67	0.71	0.43	0.88	**0.81**
	DeepOpacityNet	**0.88**	0.82	0.69	0.73	0.46	**0.89**	**0.81**
SINDI	XB5L3	0.83	0.69	0.77	0.72	0.44	0.86	0.75
	XB4L3	0.76	0.67	**0.80**	0.68	0.39	**0.88**	**0.79**
	XB3L3	**0.88**	0.78	0.70	**0.73**	0.46	**0.88**	0.78
	DeepOpacityNet	**0.88**	**0.79**	0.67	0.71	0.42	**0.88**	0.78

Supplementary Table 7: Summary of the methods used in the detection and grading of cataract in color fundus images.

Study	Method	Features	Dataset	Grades	Accuracy	
					Detection	Grading
1	Decision Trees (DT) and Genetic Algorithms	Binary features using multiple thresholds	1355	5	92.8%	81.1%
2	Semi-supervised Learning with Binary Classifiers	Wavelets, sketch, and texture features	4000*	4	96.2%	88.60%
3	Support Vector Machine (SVM)	Gray Level Cooccurrence Matrix (GLCM) features	NA	4	95.33%	87.52%
4	SVM	AlexNet, ResNet, and VGGNet features	8030	4	97.40%	95.65%
5	SVM	ResNet18 features and GLCM features	1352	4	-	94.75%
				6		92.66%
6	Convolutional Neural Networks (CNN)	Convolutional features	5620	4	93.52%	86.69%
7	Multi-class discriminant analysis	Wavelet features	445	4	90.9%	77.1%
8	Random Forests with CNN	Convolutional features	5408	6	97.04%	90.69%
9	SVM with CNN	Convolutional features	7851	4	86%	84.7%
10	Back Propagation Neural Network (BPNN)	Luminance and GLCM features	504	4	-	82.9%
11	Ensemble Learning with DT and BPNN	Texture and sketch features	374	4	95.48%	-
12	SVM with CNN	Convolutional features	800	4	100%	92.91%
13	Ensemble Learning with SVM and BPNN	Wavelet, sketch, and texture features	1239	4	93.2%	84.5%
14	SVM and RF	Wavelet and sketch features	445	4	NA	84.77%
15	Discrete BPNN	Improved HAAR wavelet features	1355	4	89.23%	94%
16	Semi-supervised learning with Bayesian Network	Wavelet and texture features	476*	4	-	88%

*These numbers represented the labeled data

Supplementary Table 8: Summary of the datasets used in the detection and grading of cataract in literature.

Study	Dataset	Normal	Mild	Moderate	Severe	Unlabeled
1	1355	-	-	-	-	-
2	2000*	676	588	382	354	3851
3	-	-	-	-	-	-
4	8030	4671	2283	675	401	-
5	1352	487	441	154	270	-
6	5620	3269	1598	472	281	-
7	445	199	148	71	27	-
8	5408	1948	1764	616	1080	-
9	7851	4671	2176	622	382	-
10	504	367	79	37	21	-
11	374	131	99	87	57	-
12	800	200	200	200	200	-
13	1239	767	246	128	98	-
14	445	199	148	71	27	-
15	1355	433	415	217	290	-
16	476	161	140	91	84	4902

*Only division of training dataset was mentioned in the study

Supplementary References

1. Xiong L, Li H, Xu LJJohe. An approach to evaluate blurriness in retinal images with vitreous opacity for cataract diagnosis. **2017**, (2017).

2. Song W, Cao Y, Qiao Z, Wang Q, Yang J-J. An improved semi-supervised learning method on cataract fundus image classification. In: *2019 IEEE 43rd Annual Computer Software and Applications Conference (COMPSAC)*). IEEE (2019).

3. Qiao Z, Zhang Q, Dong Y, Yang J-J. Application of SVM based on genetic algorithm in classification of cataract fundus images. In: *2017 IEEE International Conference on Imaging Systems and Techniques (IST)*). IEEE (2017).

4. Imran A*, et al.* Automated identification of cataract severity using retinal fundus images. **8**, 691-698 (2020).

5. Zhang H*, et al.* Automatic cataract grading methods based on deep learning. **182**, 104978 (2019).

6. Zhang L, Li J, Han H, Liu B, Yang J, Wang Q. Automatic cataract detection and grading using deep convolutional neural network. In: *2017 IEEE 14th International Conference on Networking, Sensing and Control (ICNSC)*). IEEE (2017).

7. Guo L, Yang J-J, Peng L, Li J, Liang QJCiI. A computer-aided healthcare system for cataract classification and grading based on fundus image analysis. **69**, 72-80 (2015).

8. Ran J, Niu K, He Z, Zhang H, Song H. Cataract detection and grading based on combination of deep convolutional neural network and random forests. In: *2018 International Conference on Network Infrastructure and Digital Content (IC-NIDC)*). IEEE (2018).

9. Dong Y, Zhang Q, Qiao Z, Yang J-J. Classification of cataract fundus image based on deep learning. In: *2017 IEEE International Conference on Imaging Systems and Techniques (IST)*). IEEE (2017).

10. Yang M, Yang J-J, Zhang Q, Niu Y, Li J. Classification of retinal image for automatic cataract detection. In: *2013 IEEE 15th International Conference on e-Health Networking, Applications and Services (Healthcom 2013)*). IEEE (2013).

11. Hnoohom N, Jitpattanakul A. Comparison of ensemble learning algorithms for cataract detection from fundus images. In: *2017 21st International Computer Science and Engineering Conference (ICSEC)*). IEEE (2017).

12. Pratap T, Kokil PJBSP, Control. Computer-aided diagnosis of cataract using deep transfer learning. **53**, 101533 (2019).

13. Yang J-J*, et al.* Exploiting ensemble learning for automatic cataract detection and grading. **124**, 45-57 (2016).

14. Fan W, Shen R, Zhang Q, Yang J-J, Li J. Principal component analysis based cataract grading and classification. In: *2015 17th International Conference on E-health Networking, Application & Services (HealthCom)*). IEEE (2015).

15. Zhou Y, Li G, Li HJItomi. Automatic cataract classification using deep neural network with discrete state transition. **39**, 436-446 (2019).

16. Song W, Wang P, Zhang X, Wang Q. Semi-supervised learning based on cataract classification and grading. In: *2016 IEEE 40th annual computer software and applications conference (COMPSAC)*). IEEE (2016).

Supplementary Note 1

The Age-Related Eye Disease Study 2 (AREDS2) Research Group

(NEI) National Eye Institute
Emily Y. Chew, MD, Study Chair
Frederick L. Ferris III, MD, NEI Clinical Director
John Paul SanGiovanni, ScD, Project Officer
Elvira Agrón, MA, Statistician

Coordinating Center, The EMMES Corporation
Traci Clemons, PhD, Principal Investigator
Anne Lindblad, PhD, Co-Investigator
Robert Lindblad, MD, Chief Medical Officer
Nilay Shah, MD, Medical Monitor
Robert Sperduto, MD, Consultant
Wendy McBee, MA, AREDS2 Project Director
Gary Gensler, MS, Statistician
Molly Harrington, MS, Statistician
Alice Henning, MS, Genetics Project Director
Katrina Jones Data Manager
Kumar Thotapally Programmer
Diana Tull, MA, CPS, Administrative Coordinator
Valerie Watson Systems Coordinator
Kayla Williams Data Manager
Christina Gentry, Cognitive Function Specialist
Francine Kaufman Cog. Function/Data Mgr
Chris Morrison, Cognitive Function Specialist
Elizabeth Saverino Protocol Monitor
Sherrie Schenning Protocol Monitor

Cardiovascular Outcomes Adjudicators
Denise Bonds, MD, MPH, (NHLBI)
Bradford Worrell, MD, MSc, (UVA))
Judith Hsia, MD
Jennifer Robinson, MD, MPH
Chuck Eaton, MD
Alain Bertoni, MD

Fundus Photograph Reading Center
Barbara Blodi, MD, Co-PI
Ronald P. Danis, MD, Principal Investigator
Matthew Davis, MD, Co-PI
Amitha Domalpally Co-Director
Kathy Glander Research Project Mgr
Gregory Guilfoil Research Project Mgr
Larry D. Hubbard, MA, Assoc. Dir. Grading
Kristine Johnson Inventory Assistant
Ronald Klein, MD, Co-PI
Barbara Nardi Asst. to Ron Danis
Michael Neider Assoc. Dir. Photography
Nancy Robinson Assoc. Dir Operations
Eileen Rosensteel Inventory Assistant
Hugh Wabers Photographer
Grace Zhang AREDS2 Data Manager

(01) Vision Research Foundation
Alan J. Ruby, MD, (Site PI)
Antonio Capone, Jr., MD, (Ophthalmologist)
Bawa Dass, MD, (Ophthalmologist)
Kimberly Drenser, MD, PhD, (Ophthalmologist)
Bruce R. Garretson, MD, (Ophthalmologist)
Tarek S. Hassan, MD, (Ophthalmologist)
Michael Trese, MD, (Ophthalmologist)
George A. Williams, MD, (Ophthalmologist)
Jeremy Wolfe, MD, (Ophthalmologist)
Tina Bell (Clinic Coordinator)
Mary Zajechowski (Clinic Coordinator)
Dennis Bezaire (Photographer)
Fran McIver (Photographer)

(02) Charlotte Eye Ear Nose and Throat Associates
Andrew Antoszyk, MD, (Site PI)
Justin Brown, MD, (Ophthalmologist)
David J. Browning, MD, PhD, (Ophthalmologist)
Walter Holland, MD, (Ophthalmologist)
Angella Karow (Clinic Coordinator)
Kelly Stalford (Clinic Coordinator)
Angela Price, MPH, CCRC, Dir. Of Research
Sarah Ennis (Ophthalmic Technician)
Sherry Fredenberg (Ophthalmic Technician)
Jenna Herby (Ophthalmic Technician)
Uma Balasubramaniam (Photographer)
Loraine Clark (Photographer)
Donna McClain (Photographer)

Anthony Medina, CRA, (Photographer)
Jackie Pagett (Photographer)
Stephanie Hatch Smith (Photographer)
Lynn Swartz (Photographer)
Tom Treuter (Photographer)
Michael McOwen, CRA, (Photographer)
Lynn Watson (Photographer)

(03) Devers Eye Institute
Michael Klein, MD, (Site PI)
Steven T. Bailey, MD, (Ophthalmologist)
Thomas J. Hwang, MD, (Ophthalmologist)
Andreas Lauer, MD, (Ophthalmologist)
J. Timothy Stout, MD, PhD, FACS, (Ophthalmologist)
Patty McCollum (Clinic Coordinator)
Milt Johnson (Photographer) Patrick B. Rice CRA (Photographer)

(04) Massachusetts Eye and Ear Infirmary
Ivana Kim, MD, (Site PI)
John Loewenstein, MD, (Ophthalmologist)
Joan Miller, MD, (Ophthalmologist)
Lucia Sobrin, MD, (Ophthalmologist)
Lucy Young, MD,PhD, (Ophthalmologist)
Jacqueline Sullivan (Clinic Coordinator)
Patricia Houlihan (Assistant Coordinator)
Linda Merry, RN, (Assistant Coordinator)
Ann Marie Lane (Office Manager)
Ursula Lord Bator, OD, (Ophthalmic Technician)
Claudia Evans, OD, (Ophthalmic Technician)
Sarah Brett (Photographer)
Charleen Callahan (Photographer)
Marcia Grillo (Photographer)
David Walsh (Photographer)
Kamella Lau Zimmerman (Photographer)

(05) Texas Retina Associates
Gary Edd Fish, MD, JD, (Site PI)
Rajiv Anand, MD, (Ophthalmologist)
Lori E. Coors, MD, (Ophthalmologist)
Dwain G. Fuller, MD, (Ophthalmologist)
Rand Spencer, MD, (Ophthalmologist)
Robert C. Wang, MD, (Ophthalmologist)
Karen Duignan (Clinic Coordinator)
Sally Arceneaux, COA, (Assistant Coordinator)
Hank Aguado, CRA, (Photographer)
Nicholas Hesse (Photographer)
Michael Mackens (Photographer)
Brian Swan (Photographer)

(06) National Eye Institute
Wai T. Wong, MD, PhD, (Site PI)
Catherine Cukras, MD, (Ophthalmologist)
Monica Dalal, MD, (Ophthalmologist)
Naima Jacobs-El, MD, (Ophthalmologist)
Catherine Meyerle, MD, (Ophthalmologist)
Benjamin Nicholson, MD, (Ophthalmologist)
Henry Wiley, MD, (Ophthalmologist)
Katherine Hall Shimel, RN,COT,MSN, (Clinic Coordinator)
Angel Garced, RN, (Assistant Coordinator)
Janice Oparah, RN, (Assistant Coordinator)
Greg Short, COMT, (Assistant Coordinator)
Alana Temple, RN, (Assistant Coordinator)
Babilonia Ayukawa, RN, (Phlebotomist)
Guy Foster, COT, (Ophthalmic Technician)
Darryl Hayes, COA, (Ophthalmic Technician)
Dessie Koutsandreas (Ophthalmic Technician)
Roula Nashwinter (Ophthalmic Technician)
John Rowan (Ophthalmic Technician)
Emily Y. Chew, MD, Study Chair (Project Sponsor Staff (NEI))
Michael Bono (Photographer)
Denise Cunningham (Photographer)
Marilois Palmer (Photographer)
Alicia Zetina (Photographer)

(07) Ingalls Memorial Hospital
David H. Orth, MD, (Site PI)
Kourous Rezaei, MD, Co-PI (Site Co-PI)
Joseph Civantos, MD, (Ophthalmologist)
Sohail Hasan, MD, PhD, (Ophthalmologist)

(08) Bascom Palmer Eye Institute
Philip Rosenfeld, MD,PhD, (Site PI)
Royce Chen, MD, (Ophthalmologist)
Rishi Doshi, MD, (Ophthalmologist)
Sander Dubovy, MD, (Ophthalmologist)

Kirk Packo, MD, (Ophthalmologist)
Celeste Figliulo (Clinic Coordinator)
Pam Stanberry (Phlebotomist)
Tara Farmer (Photographer)
Kiersten Nelson (Photographer)
Shannya Townsend-Patrick (Photographer)

Brian T. Kim, MD, (Ophthalmologist)
Matthew Lowrance, DO, (Ophthalmologist)
Andrew Moshfeghi, MD, (Ophthalmologist)
Zayna Nahas, MD, (Ophthalmologist)
Gary Schienbaum, MD, (Ophthalmologist)
John Vishak, MD, (Ophthalmologist)
Christina Weng, MD, (Ophthalmologist)
Zohar Yehoshua, MD, (Ophthalmologist)
Belen Rodriguez (Clinic Coordinator)
Jose Rebimbas (Assistant Coordinator)
Jane Gleichauf, RN, (Phlebotomist)
Mike Kicak (Ophthalmic Technician)
Jason Mena (Ophthalmic Technician)
Tim Odem (Ophthalmic Technician)
Elizabeth Sferza-Camp (Ophthalmic Technician)
Alicia Disgdiertt (Photographer)
Jim Oramas (Photographer)
Isabel Rams (Photographer)
Stephanie Thatcher (Photographer)

(09) The Retina Division at the Wilmer Eye Institute
Susan B. Bressler, MD, (Site PI)
Neil M. Bressler, MD, (Ophthalmologist)
Daniel Finkelstein, MD, (Ophthalmologist)
Steven H Sherman, MD, (Ophthalmologist)
Sharon Solomon, MD, (Ophthalmologist)
Howard S. Ying, MD, (Ophthalmologist)
Rita Denbow (Clinic Coordinator)
Deborah Phillips (Assistant Coordinator)
Elizabeth Radcliffe (Phlebotomist)
Judy Belt (Photographer)
Dennis Cain (Photographer)
David Emmert (Photographer)
Mark Herring (Photographer)
Jacquelyn McDonald (Photographer)

(10) Emory University Eye Center
G. Baker Hubbard, MD, (Site PI)
Chris S Bergstrom, MD, (Ophthalmologist)
Blaine Cribbs, MD, (Ophthalmologist)
Andrew Hendrick, MD, (Ophthalmologist)
Brandon Johnson, MD, (Ophthalmologist)
Philip Laird, MD, (Ophthalmologist)
Sonia Mehta, MD, (Ophthalmologist)
Timothy Olsen, MD, (Ophthalmologist)
Justin Townsend, MD, (Ophthalmologist)
Jion Yan, MD, (Ophthalmologist)
Steven Yeh, MD, (Ophthalmologist)
Linda Curtis, BSM, (Clinic Coordinator)
Judy Brower (Assistant Coordinator)
Hannah Yi (Assistant Coordinator)
Jannah Rutter Dobbs (Photographer)
Debbie Jordan (Photographer)

(11) Elman Retina Group, PA
Michael J. Elman, MD, (Site PI)
Robert A Liss, MD, (Ophthalmologist)
JoAnn Starr (Clinic Coordinator)
Jennifer Belz (Assistant Coordinator)
Charlene Putzulo (Assistant Coordinator)
Teresa Coffey (Ophthalmic Technician)
Ashley Davis (Ophthalmic Technician)
Pamela Singletary (Ophthalmic Technician)
Giorya Shabi Andreani (Photographer)
Theresa Cain (Photographer)
Daniel Ketner (Photographer)
Peter Sotirakos (Photographer)

(12) University of Wisconsin
Suresh Chandra, MD, (Site PI)
Barbara A. Blodi, MD, (Site Co-PI)
Michael M. Altaweel, MD, (Ophthalmologist) Ronald P. Danis, MD, (Ophthalmologist)
Justin L. Gottlieb, MD, (Ophthalmologist)
Michael Ip, MD, (Ophthalmologist)
Ronald Klein, MD, (Ophthalmologist)
T. Michael Nork, MD, (Ophthalmologist)
Thomas S. Stevens, MD, (Ophthalmologist) Kathryn Burke (Clinic Coordinator)
Shelly Olson (Clinic Coordinator)
Kristine Dietzman (Assistant Coordinator)
Barbara Soderling (Assistant Coordinator)
Guy Somers, RN, (Assistant Coordinator)
Angie Wealti (Assistant Coordinator)
Denise Krolnik (Photographer)
John Peterson (Photographer)

Sandra Reed (Photographer)

(13) UPMC Eye Center
Thomas Friberg, MD, (Site PI)
Andrew Eller, MD, (Ophthalmologist)
Denise Gallagher, MD, (Ophthalmologist) Leanne Labriola, DO, (Ophthalmologist) Melissa Pokrifka (Clinic Coordinator)
Aron Gedansky (Assistant Coordinator)
Natalie Anthony (Photographer)
Cassandra Grzybowski (Photographer)
Dawn Matthews (Photographer)
Sharon Murajda-Jumba (Photographer)
Jessica Toro (Photographer)

(14) Texas Retina Associates
Gary Edd Fish, MD, JD, (Site PI)
David G. Callanan, MD, (Ophthalmologist)
Wayne A. Solley, MD, (Ophthalmologist)
Patrick Williams, MD, (Ophthalmologist)
Sandy Lash (Clinic Coordinator)
Bob Boleman (Photographer)
Chris Dock (Photographer)

(15) Texas Retina Associates
Gary Edd Fish, MD, JD, (Site PI)
Michel Shami, MD, (Ophthalmologist)
Brenda Arrington (Clinic Coordinator)
Ashaki Meeks (Ophthalmic Technician)

(17) Vision Research Foundation
Alan J. Ruby, MD, (Site PI)
Alan R Margherio, MD, (Ophthalmologist)
Paul Raphaelian, MD, (Ophthalmologist)
Debra Markus (Clinic Coordinator)
Justin Langdon (Ophthalmic Technician)
Elizabeth Truax (Ophthalmic Technician)
Sandy Lewis (Photographer)
Brad Terry (Photographer)

(18) Vision Research Foundation
Alan J. Ruby, MD, (Site PI)
Amy Noffke, MD, (Ophthalmologist)
Kean Oh, MD, (Ophthalmologist)
Ramin Sarrafizadeh, MD,PhD, (Ophthalmologist)
Scott Sneed, MD, (Ophthalmologist)
Julie Hammersley, RN, (Clinic Coordinator) Serena Neal (Assistant Coordinator)
Mary Doran (Ophthalmic Technician)
Nan Jones (Ophthalmic Technician)
Lisa Preston (Ophthalmic Technician)
Heather Jessick (Photographer)
Tanya Tracy Marsh (Photographer)

(20) Center for Retina and Macular Disease
Michael Tolentino, MD, (Site PI)
Adam Berger, MD, (Ophthalmologist)
Richard Hamilton, MD, (Ophthalmologist)
David Misch, MD, (Ophthalmologist)
Suk Jin Moon, MD, (Ophthalmologist)
Dawn Sutherland (Clinic Coordinator)
Vera Dilts (Assistant Coordinator)
Sara Henderson (Assistant Coordinator)
Esmeralda Medina (Assistant Coordinator)
Donald Trueman (Assistant Coordinator)
Laura Holm, LPN, (Ophthalmic Technician)
Jason Strickland (Photographer)

(21) Delaware Valley Retina Associates
Darmakusuma Ie, MD, (Site PI)
Jeffrey L. Lipkowitz MD(Ophthalmologist) Kekul B. Shah, MD, (Ophthalmologist)
Susan Geraghty (Clinic Coordinator)
Beverly Sannazzaro (Clinic Coordinator)
Morgan Harper (Ophthalmic Technician)
Krista Bayer (Photographer)

(22) Eldorado Retina Associates, PC
Mary B. Lansing, MD, (Site PI)
Lauren B. Fox (Clinic Coordinator)
Rebecca Lee (Photographer)

(23) Georgia Retina, PC
Jay B. Stallman, MD, FACS, (Site PI)
Michael Jacobson, MD, (Ophthalmologist)
Sean Koh, MD, (Ophthalmologist)
Scott Lampert, MD, (Ophthalmologist)
John Miller, MD, (Ophthalmologist)
Mark Rivellese, MD, (Ophthalmologist)
Atul Sharma, MD, (Ophthalmologist)

(24) Henry Ford Health System - Eye Care Services
Paul A. Edwards, MD, (Site PI)
Julianne Hall (Clinic Coordinator)
Mary Monk (Clinic Coordinator)
Melanie Gutkowski (Assistant Coordinator)
Melina Mazurek (Assistant Coordinator)
Janet Murphy (Assistant Coordinator)
Katherine Gusas (Office Manager)

Robert A. Stoltz, MD, (Ophthalmologist)
Stephanie Vanderveldt, MD, (Ophthalmologist)
Leslie Marcus (Clinic Coordinator)
Starr Hendricks (Assistant Coordinator)
Ryan Hollman (Assistant Coordinator)
Grethel Betanzos (Ophthalmic Technician)
Leslie Ellorin (Ophthalmic Technician)
Shelly Fulbright (Ophthalmic Technician)
Debbie McCormick (Photographer)
Crystal Moffett (Office Manager)
David Burley (Photographer)
Nicole Chesney (Photographer)
Katie Kilgo (Photographer)
Brian Rusinek (Photographer)
Bradley Stern (Photographer)
Tracy Troszak (Photographer)
Rhonda Baker-Levingston (Pharmacist)

(25) Paducah Retinal Center
Carl W. Baker, MD, (Site PI)
Tracey Caldwell (Clinic Coordinator)
Tammy Walker (Assistant Coordinator)
Lynnette F. Lambert (Ophthalmic Technician)
Tracey Martin (Ophthalmic Technician)
Mary Jill Palmer (Ophthalmic Technician)
Tana Williams (Photographer)

(26) Retina Associates of Cleveland
Michael A Novak, MD, (Site PI)
Joseph Coney, MD, (Ophthalmologist)
David G. Miller, MD, (Ophthalmologist)
Scott Pendergast, MD, (Ophthalmologist)
Lawrence Singerman, MD, (Ophthalmologist)
Nicholas Zakov, MD, (Ophthalmologist)
Hernando Zegarra, MD, (Ophthalmologist)
Kim DuBois (Clinic Coordinator)
Susan Rath (Clinic Coordinator)
Lori Revella (Clinic Coordinator)
Tammy Brink (Ophthalmic Technician)
Kim Drury (Ophthalmic Technician)
Lisa Hogue (Ophthalmic Technician)
Mary Ilc (Ophthalmic Technician)
Connie Keller (Ophthalmic Technician)
Elizabeth McNamara (Ophthalmic Technician)
Vivian Tanner (Ophthalmic Technician)
Tamara Cunningham (Photographer)
John DuBois (Photographer)
Gregg Greanoff (Photographer)
Trina Nitzsche (Photographer)
Sheila Smith-Brewer (Photographer)

(27) Retina Associates of Kentucky
Ricky D. Isernhagen, MD, (Site PI)
John W. Kitchens, MD, (Ophthalmologist)
Thomas W. Stone, MD, (Ophthalmologist)
William J. Wood, MD, (Ophthalmologist)
Diana Holcomb (Clinic Coordinator) Virginia Therrien (Office Manager)
Michelle Buck, COT, (Ophthalmic Technician)
Jeanne Van Arsdall (Ophthalmic Technician)
Edward Slade, CRA,COA, (Photographer)

(29) Retina Center Northwest
Todd E. Schneiderman, MD, (Site PI)
David J. Spinak, MD, (Ophthalmologist)
Jackie Gaedke (Clinic Coordinator)
Heather Davis Brown (Assistant Coordinator)
Dan Helgren (Assistant Coordinator)
Jenifer Garrison Pangelinan (Photographer)

(30) Retina Group of Florida
Lawrence Halperin, MD, (Site PI)
Scott Anagnoste, MD, (Ophthalmologist)
Mandeep Dhalla, MD, (Ophthalmologist)
Krista Rosenberg, MD, (Ophthalmologist)
Barry Taney, MD, (Ophthalmologist)
W. Scott Thompson, MD, (Ophthalmologist)
Jaclyn Lopez (Clinic Coordinator)
Monica Hamlin (Assistant Coordinator)
Monica Lopez (Assistant Coordinator)
Jamie Mariano, COA, (Ophthalmic Technician)
Evelyn Quinchia (Ophthalmic Technician)

(31) Retina Northwest, PC
Michael Lee, MD, (Site PI)
Richard Dreyer, MD, (Ophthalmologist)
Irvin Handelman, MD, (Ophthalmologist)
Colin Ma, MD, (Ophthalmologist)
Mark Peters, MD, (Ophthalmologist)
Stephen Hobbs III (Clinic Coordinator)
Amanda Milliron (Assistant Coordinator)
Marcia Kopfer (Ophthalmic Technician)
Michele Connaughton (Photographer)
A. Christine Hoerner (Photographer)
R. Joseph Logan (Photographer)

Patricia Aramayo (Photographer)
Rita Veksler (Photographer)
Harry J. Wohlsein (Photographer)

(32) Retina-Vitreous Associates Medical Group
David Boyer, MD, (Site PI)
Thomas G. Chu, MD, PhD, (Ophthalmologist)
Pouya Dayani, MD, (Ophthalmologist)
David Liao, MD, (Ophthalmologist)
Roger L. Novack, MD, PhD, (Ophthalmologist)
Firas M. Rahhal, MD, (Ophthalmologist)
Richard Roe, MD, (Ophthalmologist)
Homayoun Tabandeh, MD, (Ophthalmologist)
Janet Bayramyan (Clinic Coordinator)
Tammy Gasparyan (Assistant Coordinator)
Connie Hoang (Assistant Coordinator)
Janet Kurokouchi (Assistant Coordinator)
Tammy Eileen Lo (Assistant Coordinator)
Richard Ngo (Assistant Coordinator)
Mary Ann Nguyen (Assistant Coordinator)
Michael Peyton (Assistant Coordinator)
Charles Yoon (Assistant Coordinator)
Julio Sierra (Ophthalmic Technician)
Adam Zamboni (Ophthalmic Technician)
Jeff Kessinger (Photographer)
Eric Protacio (Photographer)
Adam Smucker (Photographer)

(33) Retina Vitreous Consultants
Pamela Rath, MD, (Site PI)
Robert Bergren, MD, (Ophthalmologist)
Bernard Doft, MD, (Ophthalmologist)
Judy Liu, MD, (Ophthalmologist)
Karl Olsen, MD, (Ophthalmologist)
Lori Merlotti (Clinic Coordinator)
Willia Ingram (Assistant Coordinator)
Kellianne Marfisi (Ophthalmic Technician)
Kimberly Yeckel (Ophthalmic Technician)
Heather Schultz Carmelo (Photographer)
Amanda Fec (Photographer)
Keith McBroom (Photographer)
David Steinberg (Photographer)

(34) Sarasota Retina Institute
Marc Levy, MD, (Site PI)
Jody Abrams, MD, (Ophthalmologist)
Melvin Chen, MD, (Ophthalmologist)
Waldemar Torres, MD, (Ophthalmologist)
Peggy Jelemensky (Clinic Coordinator)
Mark Prybylski (Ophthalmic Technician)
Tara Raphael (Ophthalmic Technician)
Diana Appleby (Photographer)
Charlotte Rodman (Photographer)
Mark Sneath, COA, (Photographer)

(35) Scott and White Memorial Hospital
Robert H. Rosa, Jr., MD, (Site PI)
Vanessa Hoelscher (Clinic Coordinator)
Adelia Castano (Ophthalmic Technician)
Jocelyn Parker (Photographer)

(36) Southeastern Retina Associates, PC
John Hoskins, MD, (Site PI)
Nicholas Anderson, MD, (Ophthalmologist)
Joseph Googe, Jr., MD, (Ophthalmologist)
Tod A McMillan, MD, (Ophthalmologist)
James Miller Jr., MD, (Ophthalmologist)
Stephen Perkins, MD, (Ophthalmologist)
Kristina Oliver (Clinic Coordinator)
Jennifer Beerbower (Ophthalmic Technician)
Bruce Gilliland, OD, (Ophthalmic Technician)
Cecile Hunt (Ophthalmic Technician)
Mike Jacobus (Photographer)
Raul Lince (Photographer)
Christopher Morris (Photographer)
Sarah Oelrich (Photographer)
Jerry Whetstone (Photographer)

(37) Southern California Desert Retina Consultants, MC
Clement K. Chan, MD, (Site PI)
Steven Lin, MD, (Ophthalmologist)
Kim Walther (Clinic Coordinator)
Tiana Gonzales (Assistant Coordinator)
Lenise Myers (Ophthalmic Technician)
Kenneth Huff, COA, (Photographer)

(38) Retina Consultants of Houston
David M. Brown, MD, (Site PI)
Eric Chen, MD (Ophthalmologist)
Matthew S. Benz, MD, (Ophthalmologist)
Richard H. Fish, MD, FACS, (Ophthalmologist)
Rosa Y. Kim, MD, (Ophthalmologist)
James Major Jr, MD, (Ophthalmologist)
Tien Pei Wong, MD, (Ophthalmologist)
Charles Wycoff, MD,PhD, (Ophthalmologist)
Cassandra Cone (Clinic Coordinator)
Debbie Goates Gilaspia (Assistant Coordinator)
Nubia Landaverde (Assistant Coordinator)
Robert Smith (Assistant Coordinator)
Deneva Zamora (Assistant Coordinator)
Veronica Sneed (Ophthalmic Technician)
Melina Vela (Ophthalmic Technician)
Eric Kegley (Photographer)

(39) Wake Forest University Eye Center
Craig Greven, MD, (Site PI)
Shree Kurup, MD, (Ophthalmologist)
Charles Richards, MD, (Ophthalmologist)
Madison Slusher, MD, (Ophthalmologist)
Cara Everhart (Clinic Coordinator)
Joan Fish, RN, CCRC, (Assistant Coordinator)
Mark Clark (Photographer)
David Miller (Photographer)
Marshall Tyler, CRA, FOPS, (Photographer)

(40) West Coast Retina Medical Group, Inc.
J. Michael Jumper, MD, (Site PI)
Arthur D. Fu, MD, (Ophthalmologist)
Robert N. Johnson, MD, (Ophthalmologist)
Brandon Lujan, MD, (Ophthalmologist)
H. Richard McDonald, MD, (Ophthalmologist)
Rosa Rodriguez (Clinic Coordinator)
Nina Ansari (Ophthalmic Technician)
Jeanifer Joaquin (Ophthalmic Technician)
Silvia Linares (Ophthalmic Technician)
Lizette Lopez (Ophthalmic Technician)
Jessica Sabio (Ophthalmic Technician)
Sean Grout (Photographer)
Chad Indermill (Photographer)
Yesmin Urias (Photographer)
Roberto Zimmerman (Photographer)

(41) Veterans Affairs - Northern California Health Care System
Linda Margulies, MD, (Site PI)
Sara J. Schmidt, PharmD, (Clinic Coordinator)
Joy L. Meier, PharmD, (Assistant Coordinator)
Sherry L. Hadley COT (Ophthalmic Technician)

(42) Mid-America Retina Consultants, PA
William Rosenthal, MD, (Site PI)
Barbara Johnson, RN, (Clinic Coordinator) Lois Swafford (Office Manager)
Richard Shields, RN, (Ophthalmic Technician)
R. Scott Varner (Photographer)

(43) New York Eye and Ear Infirmary
Richard Rosen, MD, (Site PI)
Ronald Gentile, MD, (Ophthalmologist)
Melissa Rivas (Clinic Coordinator)
Katy W. Tai, CRC, (Assistant Coordinator)
Wanda Carrasquillo-Boyd (Photographer)
Robert Masini (Photographer)

(44) Ophthalmic Consultants of Long Island
Glenn Stoller, MD, (Site PI)
Ken Carnevale, MD, (Ophthalmologist)
Diane M. LaRosa, CRNO, (Clinic Coordinator)
Barbara Burger, RN, CCRC, (Assistant Coordinator)
Tereza Conway (Assistant Coordinator)
Carla Del Castillo (Assistant Coordinator)
Julissa Diaz (Assistant Coordinator)
Susan Jones (Assistant Coordinator)
Nina Mondoc (Assistant Coordinator)
Charlene Balfour (Ophthalmic Technician)
CH Vitha (Ophthalmic Technician

(45) The Research Foundation of SUNY/SB
Fadi El Baba, MD, (Site PI)
Ann Marie Lavorna (Clinic Coordinator)
Renee Jones (Assistant Coordinator)
Jean Lewis (Assistant Coordinator)
Ruth Tenzler, RN, BSN, (Assistant Coordinator)
Mary Salvas-Mladek, CRA, (Ophthalmic Technician)
Diane Van Kesteren, COA, (Ophthalmic Technician)

Jennifer Lutz (Photographer)
Barbara McGinley (Photographer)

(46) Western Carolina Retinal Associates
W. Copley McLean, Jr., MD, (Site PI)
W. Zachery Bridges, Jr., MD, (Ophthalmologist)
Cameron Stone, MD, (Ophthalmologist)
Denise Ammons (Clinic Coordinator)
Mary Lamy (Assistant Coordinator)
Andrea Menzel (Assistant Coordinator)
Lea Doll Raymer (Assistant Coordinator)
Barbara Campbell (Ophthalmic Technician)
Lisa Hawkins (Ophthalmic Technician)
Leslie Rickman (Ophthalmic Technician)
Lorraine Sherlin (Ophthalmic Technician)
Paula Price (Photographer)
Albert Sinyai (Photographer)

(47) Dean McGee Eye Institute
Ronald Kingsley, MD, (Site PI)
Reagan H. Bradford, Jr., MD, (Ophthalmologist)
Robert E. Leonard II, MD, (Ophthalmologist)
Sonny Icks (Clinic Coordinator)
Vanessa Bergman (Ophthalmic Technician)
Brittany Ross (Ophthalmic Technician)
Russ Burris (Photographer)
Amanda Butt (Photographer)
Rob Richmond (Photographer)

(48) Northwestern University, Ophthalmology
Alice Lyon, MD, (Site PI)
Manjot Gill, MD, (Ophthalmologist)
Lee Jampol, MD, (Ophthalmologist)
Rukhsana Mirza, MD, (Ophthalmologist)
Zuzanna Rozenbajgier (Clinic Coordinator)
Jeremy Chapman (Assistant Coordinator)
Lori Kaminski (Assistant Coordinator)
Andrea Degillio (Photographer)
Evica Simjanoski, CRA, (Photographer)

(49) Ophthalmic Consultants of Boston
Jeffrey Heier, MD, (Site PI)
Hyung Cho, MD, (Ophthalmologist)
Tina Scheufele Cleary, MD, (Ophthalmologist)
Darin Goldman, MD, (Ophthalmologist)
Chirag Shah, MD, (Ophthalmologist)
Trexler Topping, MD, (Ophthalmologist)
Marissa Weber, MD, (Ophthalmologist)
Torsten Wiegand, MD,PhD, (Ophthalmologist)
Jeremy Schindelheim (Clinic Coordinator)
Joy Bankert (Assistant Coordinator)
Jennifer Stone (Assistant Coordinator)
Alison Nowak (Office Manager)
Sandy Chong (Ophthalmic Technician)
Lindsay Williams (Ophthalmic Technician)
Steven Bennett (Photographer)
Dennis Donovan (Photographer)
Margaret Graham (Photographer)
Cullen Jones (Photographer)

(50) Pacific Eye Associates
Anne Fung, MD, (Site PI)
Jan-Kristine Bayabo (Clinic Coordinator)
Razelda Bosch (Assistant Coordinator)
Esperanza Cruz (Assistant Coordinator)
Ashley Emerson (Assistant Coordinator) Alycia
Fleming (Ophthalmic Technician)
Denice Barsness (Photographer)
Jorge Rodriguez (Photographer)
Marina Soboleva (Photographer)

(51) Penn State M.S. Hershey Medical Center
Ingrid U. Scott, MD, MPH, (Site PI)
Esther Bowie, MD, (Ophthalmologist)
Kimberly A Neely, MD, PhD, (Ophthalmologist)
David A. Quillen, MD, (Ophthalmologist)
Laura Walter (Clinic Coordinator)
Timothy Bennett (Photographer)
James Strong (Photographer)

(52) Palmetto Retina Center
John Wells, III, MD, (Site PI)
Lloyd Clark, MD, (Ophthalmologist)
David Johnson, MD, (Ophthalmologist)
Peggy Miller (Clinic Coordinator)
Mallie Taylor (Assistant Coordinator)
Tiffany Swinford (Ophthalmic Technician)

(53) Pennsylvania Retina Specialists, PC
Michael Banach, MD, (Site PI)
Lawrence Ho, MD, (Ophthalmologist)
Richard Lanning, MD, (Ophthalmologist)
Thomas R Pheasant, MD, (Ophthalmologist)
Jay G Prensky, MD, (Ophthalmologist)
Steven Truong, MD, (Ophthalmologist)

Robbin Spivey (Photographer)

Julia Teatsworth, COT, (Clinic Coordinator)
Michelle Dietrich (Assistant Coordinator)
Ann Wasilus (Phlebotomist)
Ann Miller (Ophthalmic Technician)
Megan Rakes (Ophthalmic Technician)
Teresa Slagle (Ophthalmic Technician)
Michelle Richards (Photographer)
Patricia Schuessler (Photographer)
Lacy Stover (Photographer)

(54) Retina Consultants, PLLC
Paul Beer, MD, (Site PI)
Naomi S. Falk, MD, (Ophthalmologist)
Mary Beth Shannon (Clinic Coordinator)
Jeannie Olmeda (Ophthalmic Technician)
Don Berdeen (Photographer)
Joseph F. Fisher, Jr. (Photographer)

(55) University of Iowa
James Folk, MD, (Site PI)
Stephen Russell, MD, (Ophthalmologist)
Barbara Taylor (Clinic Coordinator)
Connie Hinz (Assistant Coordinator)
Jean Walshire (Assistant Coordinator)
Heather Stockman (Ophthalmic Technician)
Bruce Critser (Photographer)
Stefani Karakas (Photographer)
Cindy Montague (Photographer)
Randy Verdick (Photographer)

(56) Wills Eye Hospital/Mid Atlantic Retina
Omesh Gupta, MD, (Site PI) Joseph Maguire, MD, (Site PI)
Christopher Brady, MD, (Ophthalmologist)
Francis Char DeCroos, MD, (Ophthalmologist)
Michael Dollin, MD, (Ophthalmologist)
Sunir Garg, MD, (Ophthalmologist)
Adam Gerstenblith, MD, (Ophthalmologist)
Julia Haller, MD, (Ophthalmologist)
Allen C. Ho, MD, (Ophthalmologist)
Jason Hsu, MD, (Ophthalmologist)
Richard Kaiser, MD, (Ophthalmologist)
John Pitcher, MD, (Ophthalmologist)
Carl Regillo, MD, (Ophthalmologist)
Rajiv Shah, MD, (Ophthalmologist)
Marc Spirn, MD, (Ophthalmologist)
William Tasman, MD, (Ophthalmologist)
James Vander, MD, (Ophthalmologist)
Noga Senderowitsch (Clinic Coordinator)
Michele Formoso (Assistant Coordinator)
Michelle Markun (Assistant Coordinator)
Cedric George
Christina Centinaro (Ophthalmic Technician)
Lisa Grande (Ophthalmic Technician)
Stefanie Carey (Photographer)
Elaine Liebenbaum (Photographer)

(57) Doheny Eye Institute USC
SriniVas Sadda, MD, (Site PI)
Mark Humayun, MD, PhD, (Ophthalmologist)
Rachel Sierra (Clinic Coordinator)
Elizabeth Corona (Assistant Coordinator)
Margaret Padilla (Assistant Coordinator)
Moonseok Nu (Office Manager)
Sylvia Ramos (Ophthalmic Technician)
Cullen Barnett (Photographer)
Glenn Currie (Photographer)
Cornelia Gottlieb (Photographer)

(58) The Retina Group of Washington
Richard Garfinkel, MD, (Site PI)
Daniel Berinstein, MD, (Ophthalmologist)
Marcus Colyer, MD, (Ophthalmologist)
William Deegan, III, MD, (Ophthalmologist)
Michael Min-Shyue Lai, MD, (Ophthalmologist)
Robert Murphy, MD, (Ophthalmologist)
Michael Osman, MD, (Ophthalmologist)

(60) The Medical College of Wisconsin
Judy E. Kim, MD, (Site PI)
Jane Bachman, OD, (Ophthalmologist)
Thomas B. Connor, Jr., MD, (Ophthalmologist)
Dennis P. Han, MD, (Ophthalmologist)
Kimberly Stepian, MD, (Ophthalmologist)
David V. Weinberg, MD, (Ophthalmologist)
William J. Wirostko, MD, (Ophthalmologist)

Michael Rivers, MD, (Ophthalmologist)
Reginald Sanders, MD, (Ophthalmologist)
Manfred A. von Fricken, MD, (Ophthalmologist)
Debbie Oliver (Clinic Coordinator)
Jeanne Kirshon (Assistant Coordinator)
Tanya Alexander Snowden (Assistant Coordinator)
Thomas Blondo (Ophthalmic Technician)
Alysia Cronise (Ophthalmic Technician)
Vanessa Denny (Ophthalmic Technician)
Kylie Mendez (Ophthalmic Technician)
Janine Newgen (Ophthalmic Technician)
Justin Davis (Photographer)
Mike Flory (Photographer)
Robert Frantz (Photographer)
Bryan Murphy (Photographer)
Steve Rauch (Photographer)
Krissa Packard (Clinic Coordinator)
Tracy Kaczanowski (Assistant Coordinator)
Vesper Williams (Assistant Coordinator)
Vicki Barwick (Ophthalmic Technician)
Judy Flanders (Ophthalmic Technician)
Dennis Backes (Photographer)
Joe Beringer (Photographer)
Kristy Keller (Photographer)
Kathy Selchert (Photographer)

(61) John Moran Eye Center
Paul Bernstein, MD, PhD, (Site PI)
Michael Teske, MD, (Ophthalmologist)
Albert Vitale, MD, (Ophthalmologist)
Susan Allman, COA, (Clinic Coordinator)
Bonnie Carlstrom COA (Assistant Coordinator)
Kimberley Wegner (Assistant Coordinator)
Anne Haroldsen (Office Manager)
Deborah Harrison, MS, (Office Manager)
Cyrie Fry (Photographer)
James Gilman, CRA, (Photographer)
Glen Jenkins (Photographer)
Paula Morris, CRA, (Photographer)

(63) Loma Linda University
Michael Rauser, MD, (Site PI)
Joseph Fan, MD, (Ophthalmologist)
Mukesh Suthar, MD, (Ophthalmologist)
Gisela Santiago (Clinic Coordinator)
Kara Rollins Halsey (Assistant Coordinator)
Christy Quesada (Assistant Coordinator)
William Kiernan, OD, (Ophthalmic Technician)
Jesse Knabb (Photographer)

(64) Baylor College of Medicine
Richard Alan Lewis, MD, MS, (Site PI)
Cindy Dorenbach, COT, (Clinic Coordinator)
Steven Spencer, COMT, (Ophthalmic Technician)
Dana Barnett (Photographer)
Joseph Morales, CRA, (Photographer)

(66) Carolina Retina Center
Barron C. Fishburne, MD, (Site PI)
Jeffrey G. Gross, MD, (Ophthalmologist)
Michael A. Magee, MD, (Ophthalmologist)
Amy Flowers (Clinic Coordinator)
Angie McDowell (Ophthalmic Technician)
Randall Price (Photographer)

(67) Case Western Reserve University
Suber Huang, MD, MBA, (Site PI)
Johnny Tang, MD, (Ophthalmologist)
Shawn Wilker, MD, (Ophthalmologist)
Cherie Hornsby (Clinic Coordinator)
Lisa Ferguson (Assistant Coordinator)
Kirk Krogstad (Assistant Coordinator)
Riva Adamovsky (Ophthalmic Technician)
Peggy Allchin (Ophthalmic Technician)
Kathleen Carlton (Ophthalmic Technician)
Claudia Clow (Ophthalmic Technician)
Kelly Sholtis (Ophthalmic Technician)
Stephanie Burke (Photographer)
Mark Harrod (Photographer)
Stacie Hrvatin (Photographer)
Geoffrey Pankhurst (Photographer)

(69) Eye Foundation of Kansas City
Nelson R. Sabates, MD, (Site PI)
Michael Cassell, MD, (Ophthalmologist)
Komal Desai, MD, (Ophthalmologist)
Abraham Poulose, MD, (Ophthalmologist)
Felix Sabates, MD, (Ophthalmologist)
Yin Chen (Clinic Coordinator)
Gary Gallimore, COMT, (Photographer)
Yolanda Konior (Photographer)

(70) Jones Eye Institute - UAMS
Nicola Kim, MD, (Site PI)
Sami Uwaydat (Ophthalmologist)
Deborah Troillett (Clinic Coordinator)
Karen Aletter (Photographer)

(71) Kresge Eye Institute
Robert N. Frank, MD, (Site PI)
Gary Abrams, MD, (Ophthalmologist)
James Puklin, MD, (Ophthalmologist)
Asheesh Tewari, MD, (Ophthalmologist)
Cheryl Milanovic (Clinic Coordinator)
Melanie Bailey (Photographer)
David Griffith (Photographer)
Dena McDonald (Photographer)
Kit Morehead (Photographer)
Zlatan Sadikovic (Photographer)
Lisa Schillace (Photographer)
Elizabeth Silvis (Photographer)

(72) Colorado Retina Associates PC
Brian Joondeph, MD, (Site PI)
Nancy Christmas, MD, (Ophthalmologist)
David Johnson, MD, (Ophthalmologist)
Alan Kimura, MD, (Ophthalmologist)
Mimi Liu, MD, (Ophthalmologist)
Stephen Petty, MD, (Ophthalmologist)
John Zilis, MD, (Ophthalmologist)
Jenny Benitez (Clinic Coordinator)
Cassandra Berryman Catlett (Assistant Coordinator)
Eric Fluegel, RN, (Assistant Coordinator)
Shane Mowry (Photographer)
Hoang Nguyen (Photographer)
David Reflow (Photographer)

(74) UNC Department of Ophthalmology
Odette M. Houghton, MD, (Site PI)
Seema Garg, MD, PhD, (Ophthalmologist)
Maurice B. Landers, MD, (Ophthalmologist)
Travis Meredith, MD, (Ophthalmologist)
Sandy Barnhart, MPH, (Clinic Coordinator)
Megha Karmalkar (Assistant Coordinator)
Debra Cantrell (Photographer)
Rona Lyn Esquejo-Leon (Photographer)
Linda Manor (Pharmacist)
Sue Pope (Pharmacist)
David Stines (Pharmacist)
Amelia Stokely (Pharmacist)

(75) University Health Care - Mason Eye Institute
Dean Hainsworth, MD, (Site PI)
Dyann Helming (Clinic Coordinator)
Debbie Eichelberger (Office Manager)
Mary Paige Leaton (Ophthalmic Technician)
Chuck Hamm (Photographer)

(76) University of Tennessee HSC
Edward Chaum, MD, PhD, (Site PI)
Alessandro Iannaccone, MD, (Ophthalmologist)
Barbara Jennings, MA,OD, (Clinic Coordinator)
Tracy Murray (Ophthalmic Technician)
Joe Mastellone (Photographer)

(77) Fletcher Allen Health Care
Robert Millay, MD, (Site PI)
Brian Kim, MD, (Ophthalmologist)
Theresa Goddard (Clinic Coordinator)
Liza Jarrett Beaudette (Ophthalmic Technician) Nina Changelian-Aitken (Ophthalmic Technician)
Fernando Corrada (Photographer)
Jason Dubuque (Photographer)

(78) Mayo Clinic
Raymond Iezzi, MD, (Site PI)
Sophie J. Bakri, MD, (Ophthalmologist)
Jose S. Pulido, MD, (Ophthalmologist)
Diane Vogen (Clinic Coordinator)
Rebecca Nielsen, LPN, (Assistant Coordinator)
Karin Berg (Ophthalmic Technician)
Jean Burrington, COA, (Ophthalmic Technician)
Shannon Howard, COA, (Ophthalmic Technician)
Joan Overend (Ophthalmic Technician)
Zbigniew Krason (Photographer)
Denise Lewison (Photographer)
Thomas Link, CRA, (Photographer)

(79) The Retina Institute
Kevin J. Blinder, MD, (Site PI)
Nicholas E. Engelbrecht, MD, (Ophthalmologist)
M. Gilbert Grand, MD, (Ophthalmologist)

(80) Yale University Eye Center
Ron Adelman, MD, (Site PI)
John Huang, MD, (Ophthalmologist)
James Kempton, MD, (Ophthalmologist)
Aaron Parnes, MD, (Ophthalmologist)

Daniel P. Joseph, MD,PhD, (Ophthalmologist)
Gaurav K. Shah, MD, (Ophthalmologist)
Bradley Smith, MD, (Ophthalmologist)
Matthew Thomas, MD, (Ophthalmologist)
Rhonda Weeks (Clinic Coordinator)
Lynda Boyd (Ophthalmic Technician)
Dana Gabel (Photographer)
Jennifer Dupont (Clinic Coordinator)
Elizabeth Perotti (Assistant Coordinator)
Victoria Donaldson (Ophthalmic Technician)
Kenneth Fong (Photographer)
Pamela Ossorio (Photographer)

(81) Vanderbilt Eye Institute
Anita Agarwal, MD, (Site PI)
Paul Sternberg, MD, (Ophthalmologist)
Sandy Owings (Clinic Coordinator)
Tony Adkins (Photographer)
Elaine Lok (Photographer)
Garvin Munn (Photographer)
Buddy Skellie (Photographer)

(82) UMDNJ
Neelakshi Bhagat, MD,MPH, (Site PI)
Monique S. Roy, MD, (Ophthalmologist)
Marco Zarbin, MD, PhD, (Ophthalmologist)
Catherine Fay (Clinic Coordinator)
Michael Lazar (Photographer)
Beth Malpica (Photographer)
Tatiana Mikheyav (Photographer)

(84) The University of Illinois
Lawrence Ulanski II, MD, (Site PI)
Jennifer Lim, MD, (Ophthalmologist)
Marcia Niec, BS CCRP, (Clinic Coordinator)
Tametha Johnson (Ophthalmic Technician)
Yesenia Ovando (Ophthalmic Technician)
Catherine Nail Carroll (Photographer)
Mark Janowicz (Photographer)

(85) Jules Stein Eye Institute
Steven Schwartz, MD, (Site PI)
David Cupp, MD, (Ophthalmologist)
Michael Gorin, MD, PhD, (Ophthalmologist)
Gad Heilweil, MD, (Ophthalmologist)
Hamid Hosseini, MD, (Ophthalmologist)
Jean-Pierre Hubschman, MD, (Ophthalmologist)
Allan Kreiger, MD, (Ophthalmologist)
Tara Young McCannel, MD, (Ophthalmologist)
Carolyn Pan, MD, (Ophthalmologist)
David Sarraf, MD, (Ophthalmologist)
Irena Tsui, MD, (Ophthalmologist)
Joshua Udoetek MD(Ophthalmologist)
Vinad Voleti, MD, (Ophthalmologist)
Logan Hitchcock (Clinic Coordinator)
Rosaleen Ostrick (Assistant Coordinator)
Melissa Chun, OD, (Ophthalmic Technician)
Jennie Kageyama, OD, (Ophthalmic Technician)
Nilo Davila (Photographer)
Kristin Lipka (Photographer)
Christina Shin (Pharmacist)

(86) Univ. of Alabama at Birmingham
Cynthia Owsley, PhD, (Site PI)
Michael Albert, Jr., MD, (Ophthalmologist)
Richard Feist, MD, (Ophthalmologist)
John Mason, MD, (Ophthalmologist)
Martin Thomley, MD, (Ophthalmologist)
Angelia Johnson (Clinic Coordinator)
Mark Clark (Assistant Coordinator)
Tracy Emond (Assistant Coordinator)
Joanna Hamela (Assistant Coordinator)
Angela Marsh (Office Manager)
Karen Searcey (Office Manager)
Kia Rookard (Ophthalmic Technician)

(87) UT Southwestern Medical Center
Yu-Guang He, MD, (Site PI)
Rafael L. Ufret-Vincenty, MD, (Ophthalmologist)
Mike Molai (Clinic Coordinator)
William Anderson (Photographer)
John Horna (Photographer)

(88) Ohio State University
Alan Letson, MD, (Site PI)
Colleen Cebulla, MD,PhD, (Ophthalmologist)

(89) Duke University
Cynthia Toth, MD, (Site PI)
Glenn Jaffe, MD, (Ophthalmologist)

Susie Chang, MD, (Ophthalmologist)
Fred Davidorf, MD, (Ophthalmologist)
Jill Salerno (Clinic Coordinator)
Laura Sladoje (Office Manager)
Christina Stetson (Office Manager)
Jeri Perry (Ophthalmic Technician)
Scott Savage (Photographer)

Stefanie Schuman, MD, (Ophthalmologist)
Neeru Sarin, MBBS, (Clinic Coordinator)
Jim Crowell (Photographer)
Tiffanie Keaton (Photographer)
Michael Kelly (Photographer)
Brian Lutman (Photographer)
Marriner Skelly (Photographer)
Lauren Welch (Photographer)

(90) University of California, Davis Lawrence
Morse, MD, PhD, (Site PI)
Allan Hunter, MD, (Ophthalmologist)
Susanna Soon-Chun Park MD, PhD
(Ophthalmologist)
Cynthia Wallace (Clinic Coordinator)
Ember Dhillon (Assistant Coordinator)
Marisa Salvador (Assistant Coordinator)
Barbara Holderreed (Office Manager)
Karishma Chandra (Photographer)
Sashi Kaur (Photographer)
Ellen Redenbo (Photographer)
Smiley Hom (Pharmacist)

(91) Manhattan Eye, Ear and Throat Hospital
Michael Cooney, MD, (Site PI)
Irene Barbazetto, MD, (Ophthalmologist)
James M. Klancnik, Jr., MD, (Ophthalmologist)
John A. Sorenson, MD, (Ophthalmologist)
Lawrence Yannuzzi, MD, (Ophthalmologist)
Maria Scolaro (Clinic Coordinator)
Eugene Agresta (Photographer)
Nancy Gonzalez (Photographer)

(92) University of Florida
Sandeep Grover, MD, (Site PI)
K.V. Chalam, MD,PhD, (Ophthalmologist)
Shailesh Gupta, MD, (Ophthalmologist)
Christopher Lyons (Clinic Coordinator)
Wenhua Li (Assistant Coordinator)
Chirag Patel, MD, (Assistant Coordinator)
Jose Carrion (Photographer)

(93) Shiley Eye Center - UCSD
Henry Ferreyra, MD, (Site PI)
Amberly Rodriguez (Clinic Coordinator)
Iliana Molina (Assistant Coordinator)
Gabriel Balea (Photographer)
Pam Emory (Photographer)
Marlene Rico (Photographer)
Giorgio Siqueiros (Photographer)

(94) Scheie Eye Institute
Alexander J. Brucker, MD, (Site PI)
Joshua Dunaief, MD, (Ophthalmologist)
Juan Grunwald, MD, (Ophthalmologist)
Benjamin Kim, MD, (Ophthalmologist)
Albert M. Maguire, MD, (Ophthalmologist)
Brian VanderBeek, MD, (Ophthalmologist)
Sheri Drossner, MSW, (Clinic Coordinator)
Joan DuPont (Assistant Coordinator)
Rebecca Salvo (Assistant Coordinator)
Jim Berger (Photographer)
Cheryl Devine (Photographer)
Bill Nyberg (Photographer)
Laurel Weeney (Photographer)

(95) University of Rochester Eye Institute
David DiLoreto, MD, (Site PI)
Mina Chung, MD, (Ophthalmologist)
Valerie Davis (Clinic Coordinator)
Peter MacDowell (Assistant Coordinator)
George O Gara (Assistant Coordinator)
Daniel Castillo (Ophthalmic Technician)
Andrea Czubinski (Ophthalmic Technician)
Melissa Keim (Ophthalmic Technician)
Brandi Hardy (Photographer)
Rachel Grunhaus Hollar (Photographer)\
Lynn Schueckler (Photographer)

(148) NorthShore University HealthSystems
Alice T. Lyon, MD, (Site PI)
Aaron Weinberg, MD, Site Co-PI
Mira Shiloach (Clinic Coordinator)
Nicole Pelkofer (Ophthalmic Technician)
Qin Zhou (Ophthalmic Technician)
Laura McPoland (Photographer)

(179) Washington University School of Medicine
Rajendra Apte, MD, PhD, (Site PI)
P. Kumar Rao, MD, (Ophthalmologist)
Sam Pistorius (Clinic Coordinator)
Jamie Kambarian (Assistant Coordinator)
Eve Adcock (Ophthalmic Technician)
Sarah Gould (Ophthalmic Technician)
Melanie Quinn (Ophthalmic Technician)
Rhonda Curtis (Photographer)

Amy Frost (Photographer)
Charla Meyer (Photographer)
Greg Rathert (Photographer)

Data Safety & Monitoring Committee
Janet Wittes, PhD (chair), Statistics Collaborative
Alan F. Cruess MD FRCSC, Dalhousie University
Maureen G. Maguire, PhD, University of Pennsylvania
Susan T. Mayne, PhD, Yale University
Scott D. Solomon, MD, Harvard Medical School
Alison Wichman, MD, National Institutes of Health
C. Pat Wilkinson, MD, Greater Baltimore Medical Center

Centers for Disease Control (CDC)
Rosemary L Schleicher, PhD, Lead Investigator, Fat-soluble Vitamins & Fatty Acids Supervisor
Mary M Kimberly, PhD, Lipids Supervisor Kathleen L Caldwell, PhD, Zinc & Copper Supervisor
Madhulika Chaudhary-Webb, MS , Fat-soluble Vitamins Team Lead
Carissa D Powers, BS, Fatty Acids Team Lead
Pamela G Olive, MT(ASCP), Lipids Analyst Shelton Stribling, BS , Lipids Analyst
Amir Makhmudov, PhD , Zinc & Copper Analyst
Shakirova Gulchekhra, MS, Zinc & Copper Analyst
Graylin Mitchell, MS, Zinc & Copper Analyst
Ron Albalak, MS, Zinc & Copper Analyst Elizabeth C Pendergrast, MT(ASCP), Fat-soluble Vitamins & Fatty Acids Analyst
Shahzad S Momin, BS, Fatty Acids Analyst
