# Supplementary material for: A deep network DeepOpacityNet for detection of cataracts from color fundus photographs
Source: Commun Med (Lond). 2023 Dec 16;3:184. doi: 10.1038/s43856-023-00410-w (PMC10725427; doi:10.1038/s43856-023-00410-w)
Supplement: Supplementary file 2 — Description of Additional Supplementary Files [file 43856_2023_410_MOESM2_ESM.pdf]

Description of Additional Supplementary Data

**Supplementary Data 1**	Full visualization of DeepOpacityNet and transfer learning networks on the subjective test set.
**Supplementary Data 2**	The ground truth and predictions of all models on the internal test set.
**Supplementary Data 3**	The ground truth, gradings of the ophthalmologists, and predictions of DeepOpacityNet on the subjective test subset.
**Supplementary Data 4**	The ground truth and predictions of all models on the SiMES dataset.
**Supplementary Data 5**	The ground truth and predictions of all models on the SCES dataset.
**Supplementary Data 6**	The ground truth and predictions of all models on the SINDI dataset.
