# Supplementary material for: A deep network DeepOpacityNet for detection of cataracts from color fundus photographs
Source: Commun Med (Lond). 2023 Dec 16;3:184. doi: 10.1038/s43856-023-00410-w (PMC10725427; doi:10.1038/s43856-023-00410-w)
Supplement: Supplementary file 3 — Supplementary Data 1 [file 43856_2023_410_MOESM3_ESM.pdf]

# Full Visualization of the Best-Performing Development Networks

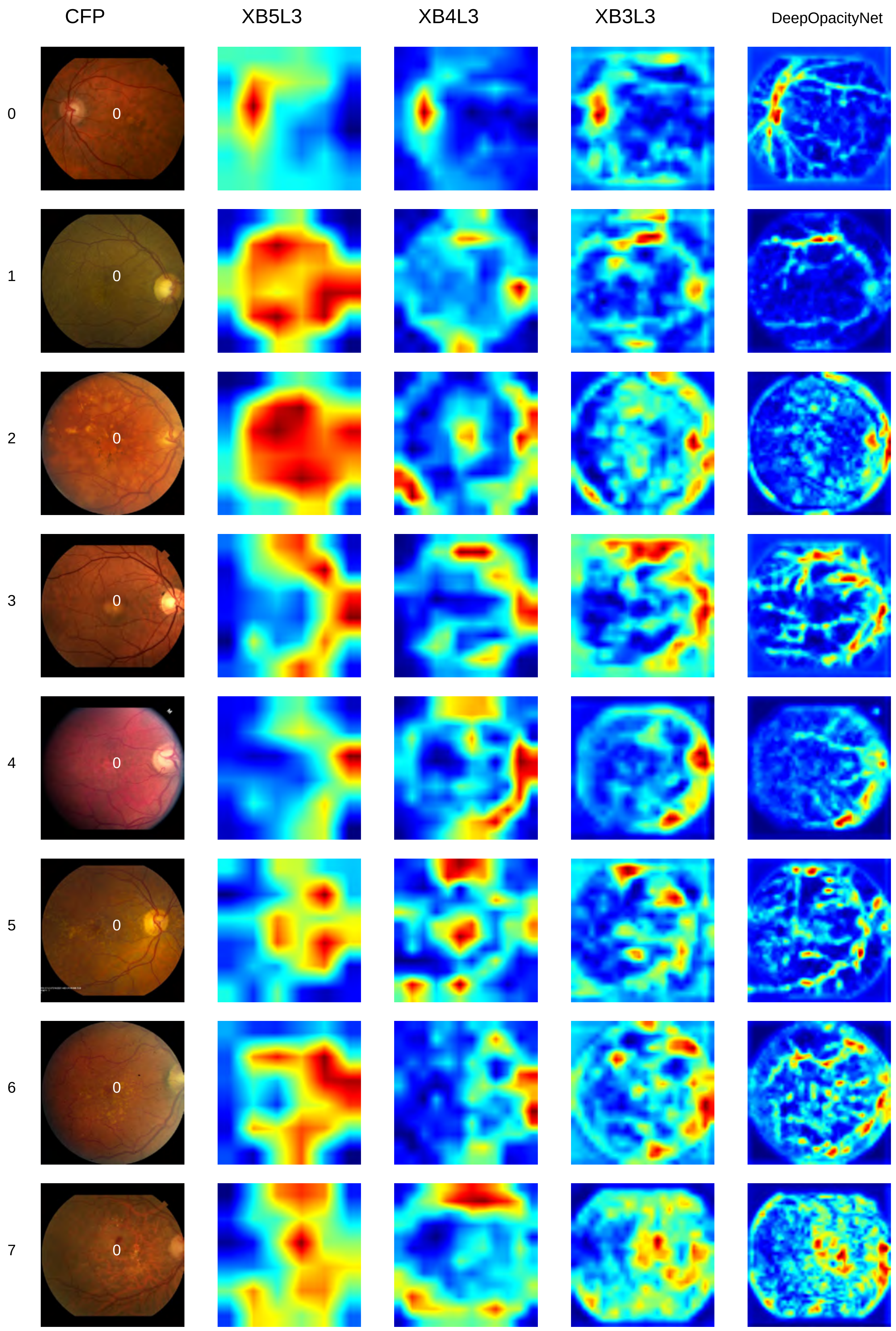
CFP
XB5L3
XB4L3
XB3L3
DeepOpacityNet
0
1
2
3
4
5
6
7
0
0
0
0
0
0
0
0

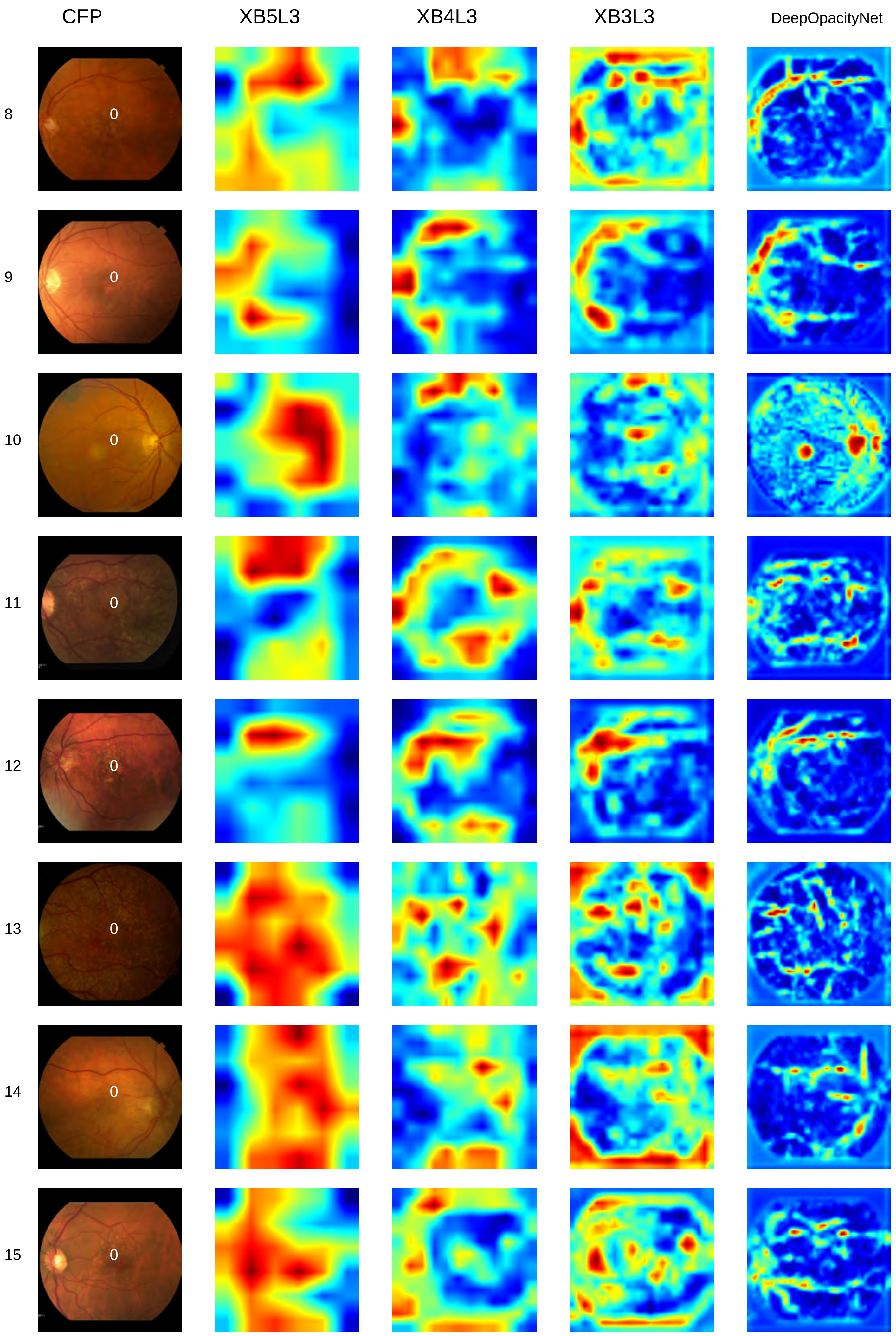
CFP
XB5L3
XB4L3
XB3L3
DeepOpacityNet
8
9
10
11
12
13
14
15
0
0
0
0
0
0
0
0

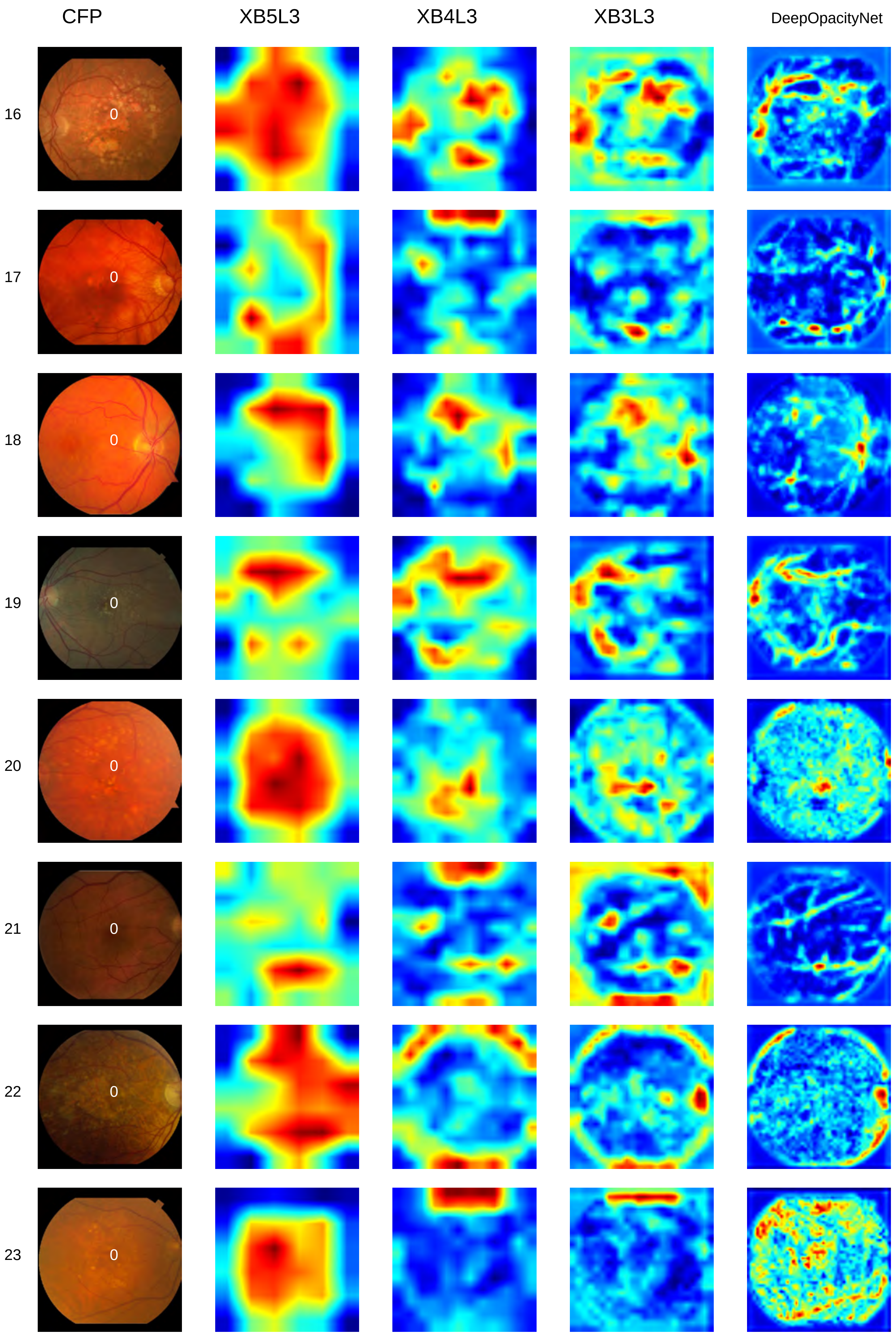

CFP
XB5L3
XB4L3
XB3L3
DeepOpacityNet
16
0
17
0
18
0
19
0
20
0
21
0
22
0
23
0

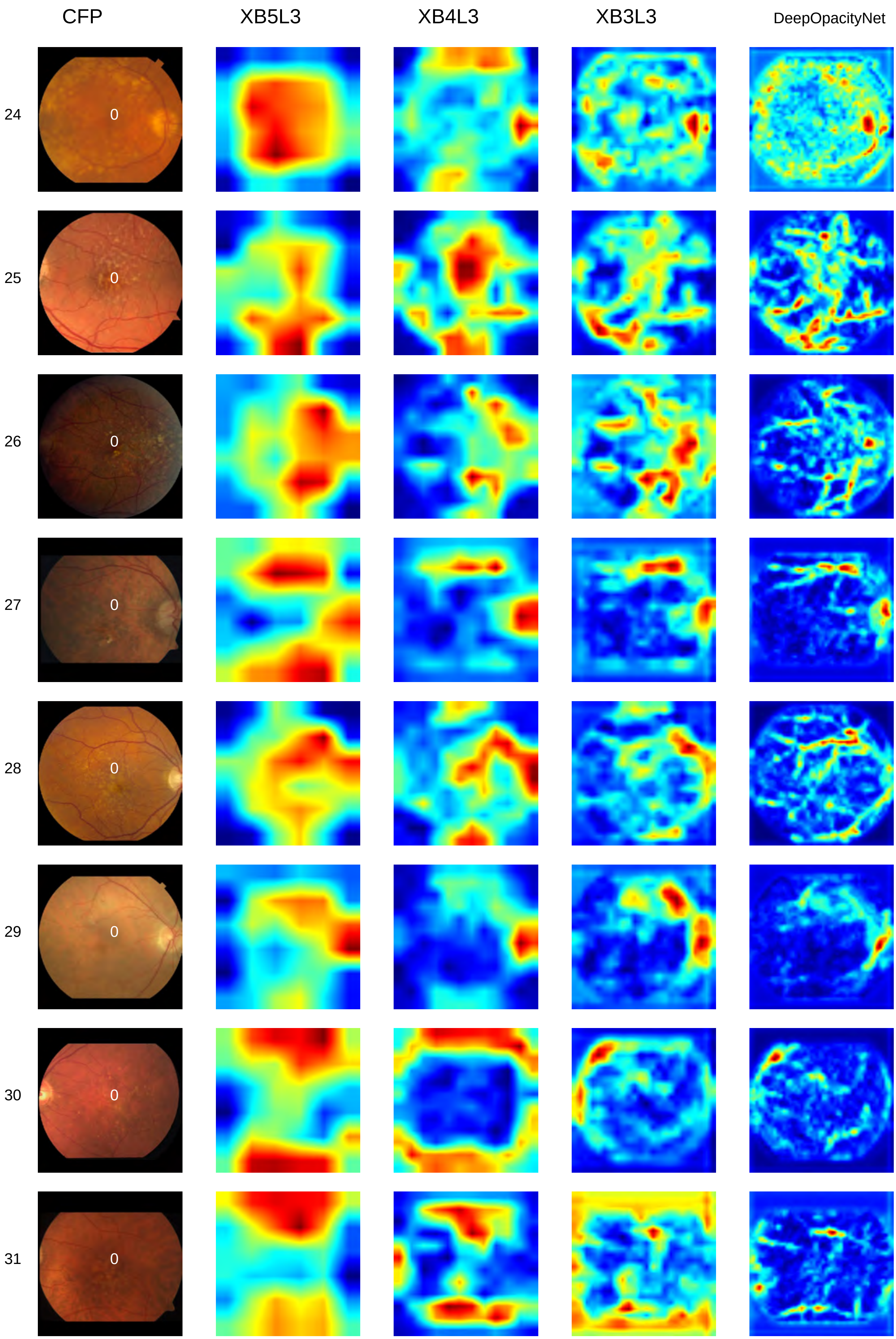

CFP
XB5L3
XB4L3
XB3L3
DeepOpacityNet
24
25
26
27
28
29
30
31
0
0
0
0
0
0
0
0

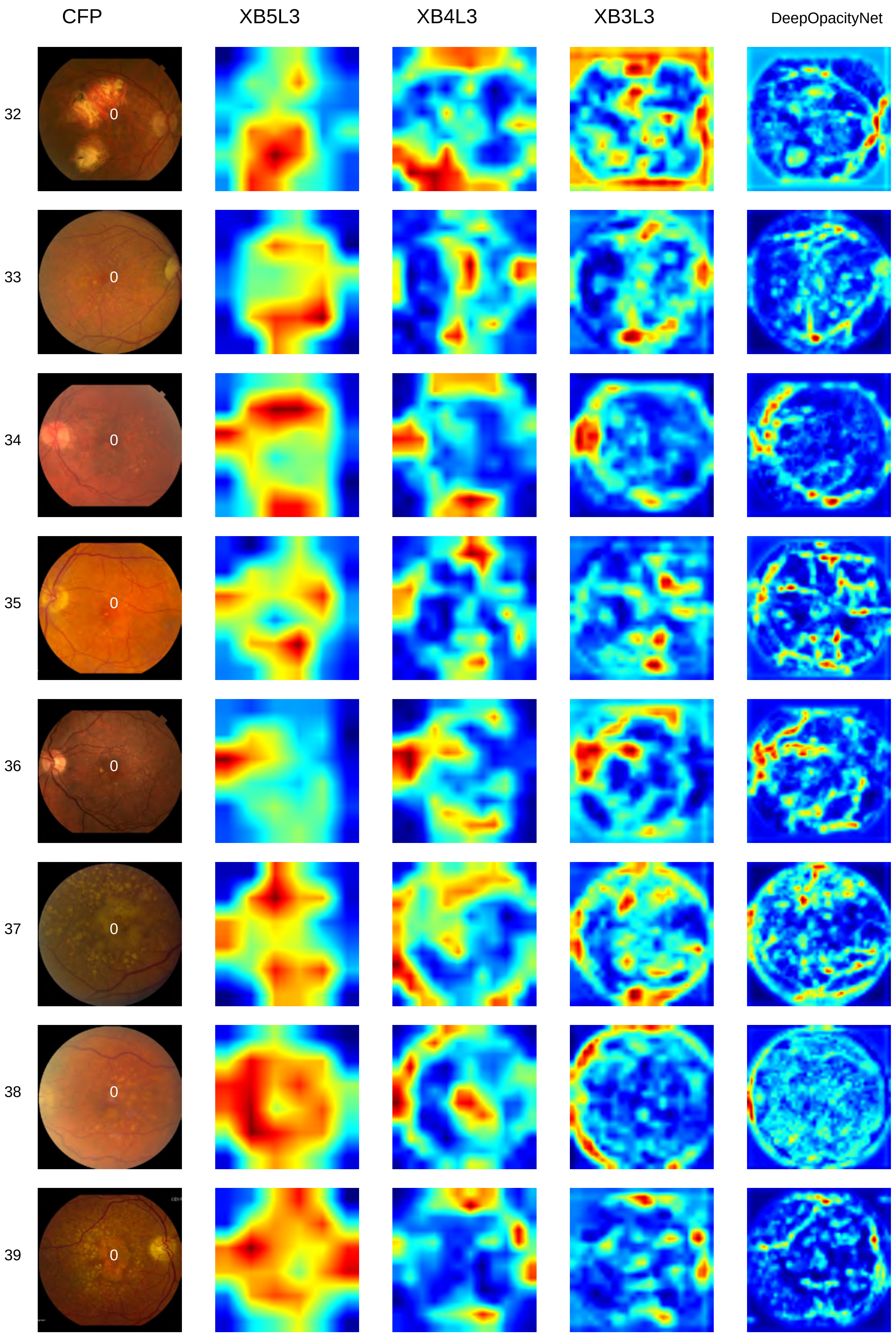

CFP
XB5L3
XB4L3
XB3L3
DeepOpacityNet
32
33
34
35
36
37
38
39
0
0
0
0
0
0
0
0

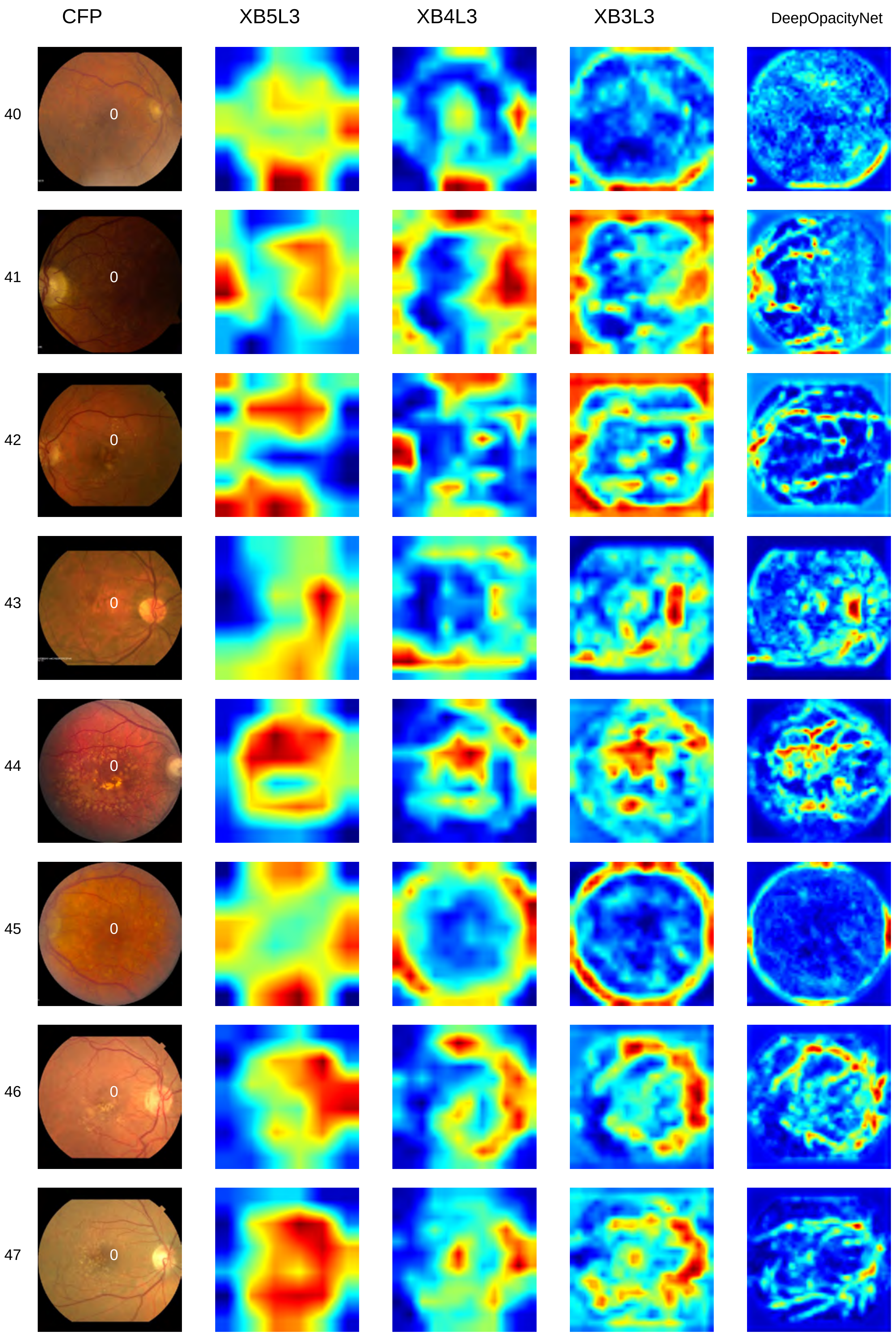
CFP
XB5L3
XB4L3
XB3L3
DeepOpacityNet
40
0
41
0
42
0
43
0
44
0
45
0
46
0
47
0

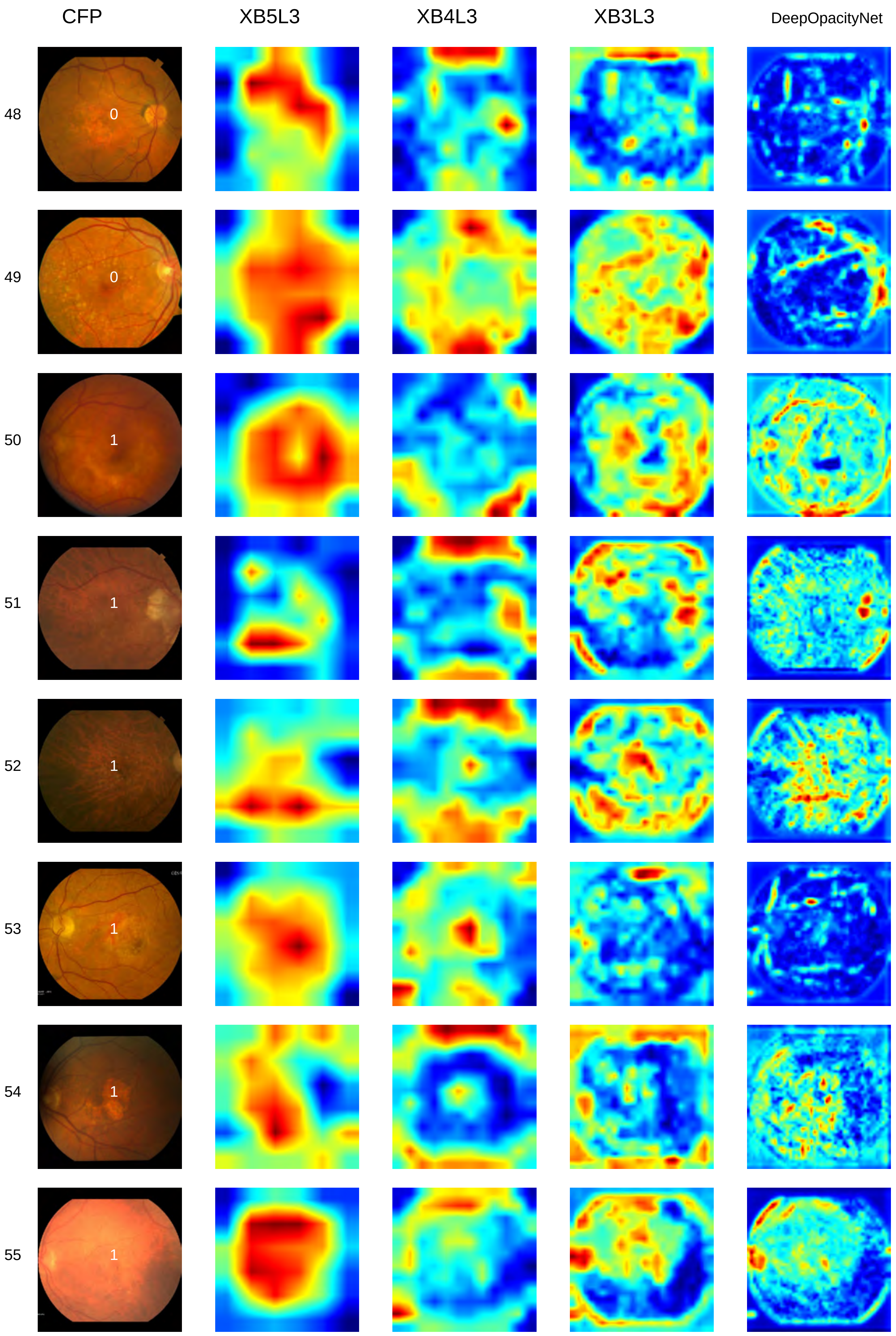

CFP
XB5L3
XB4L3
XB3L3
DeepOpacityNet
48
0
49
0
50
1
51
1
52
1
53
1
54
1
55
1

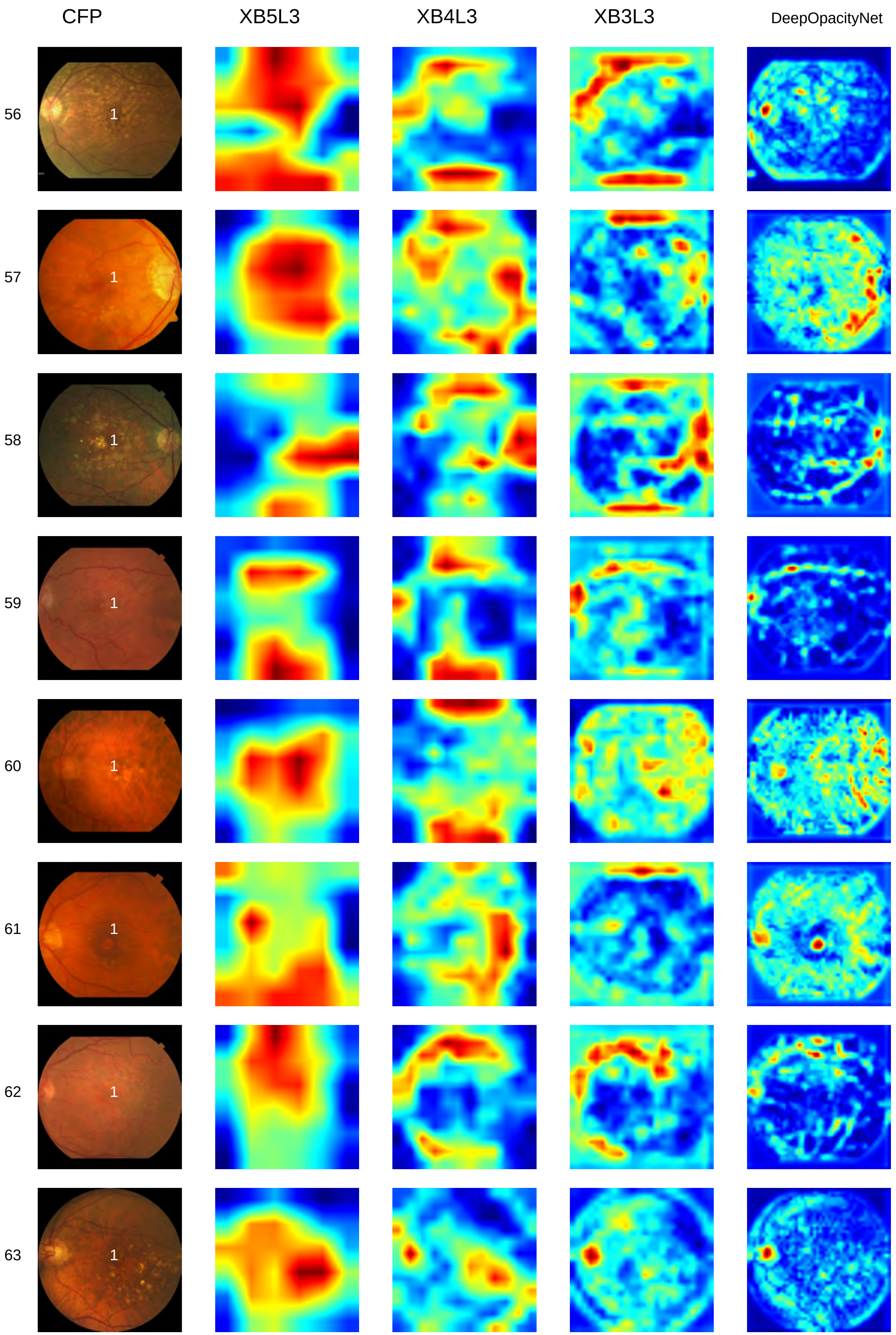
CFP
XB5L3
XB4L3
XB3L3
DeepOpacityNet
56
57
58
59
60
61
62
63
1

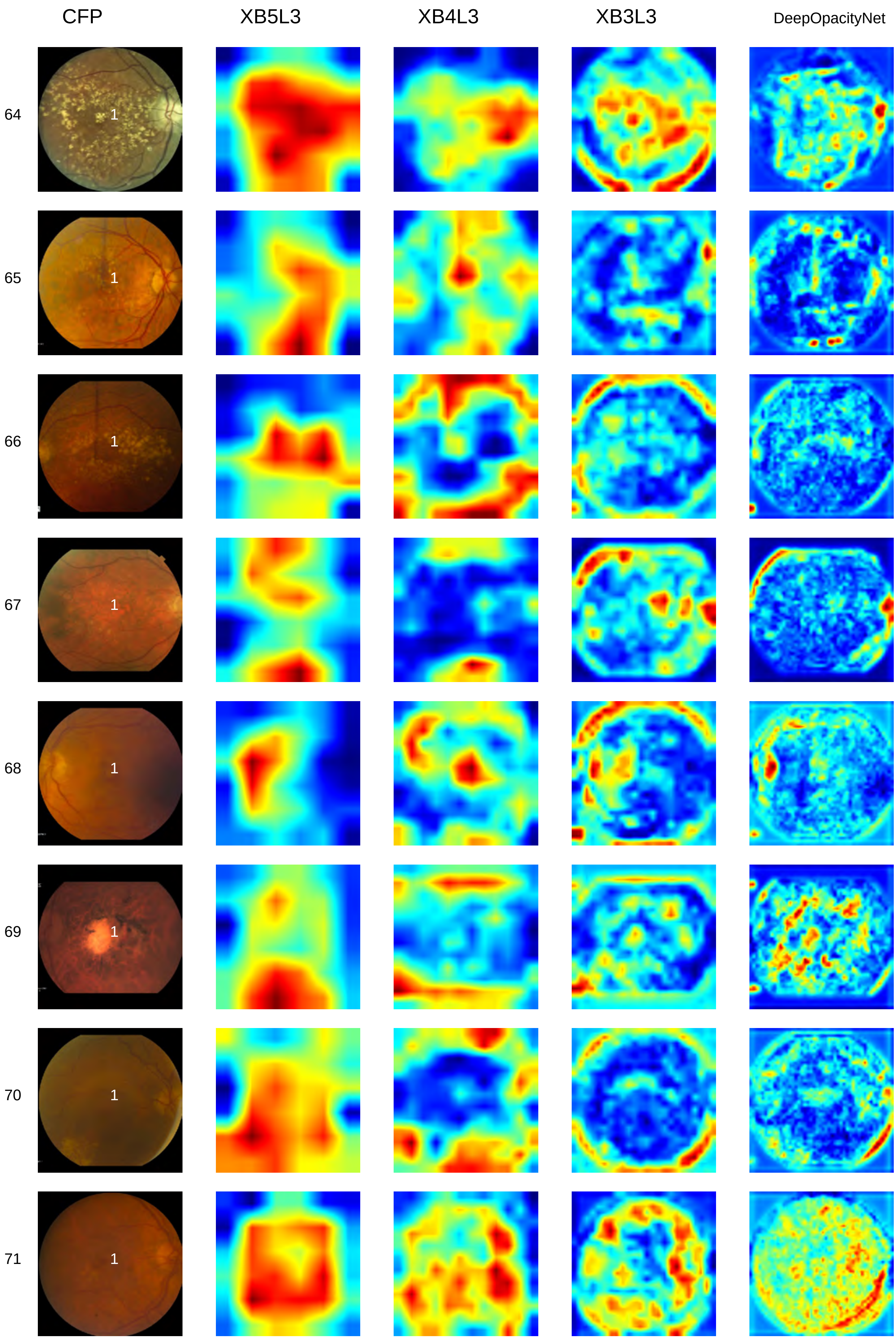
CFP
XB5L3
XB4L3
XB3L3
DeepOpacityNet
64
65
66
67
68
69
70
71
1

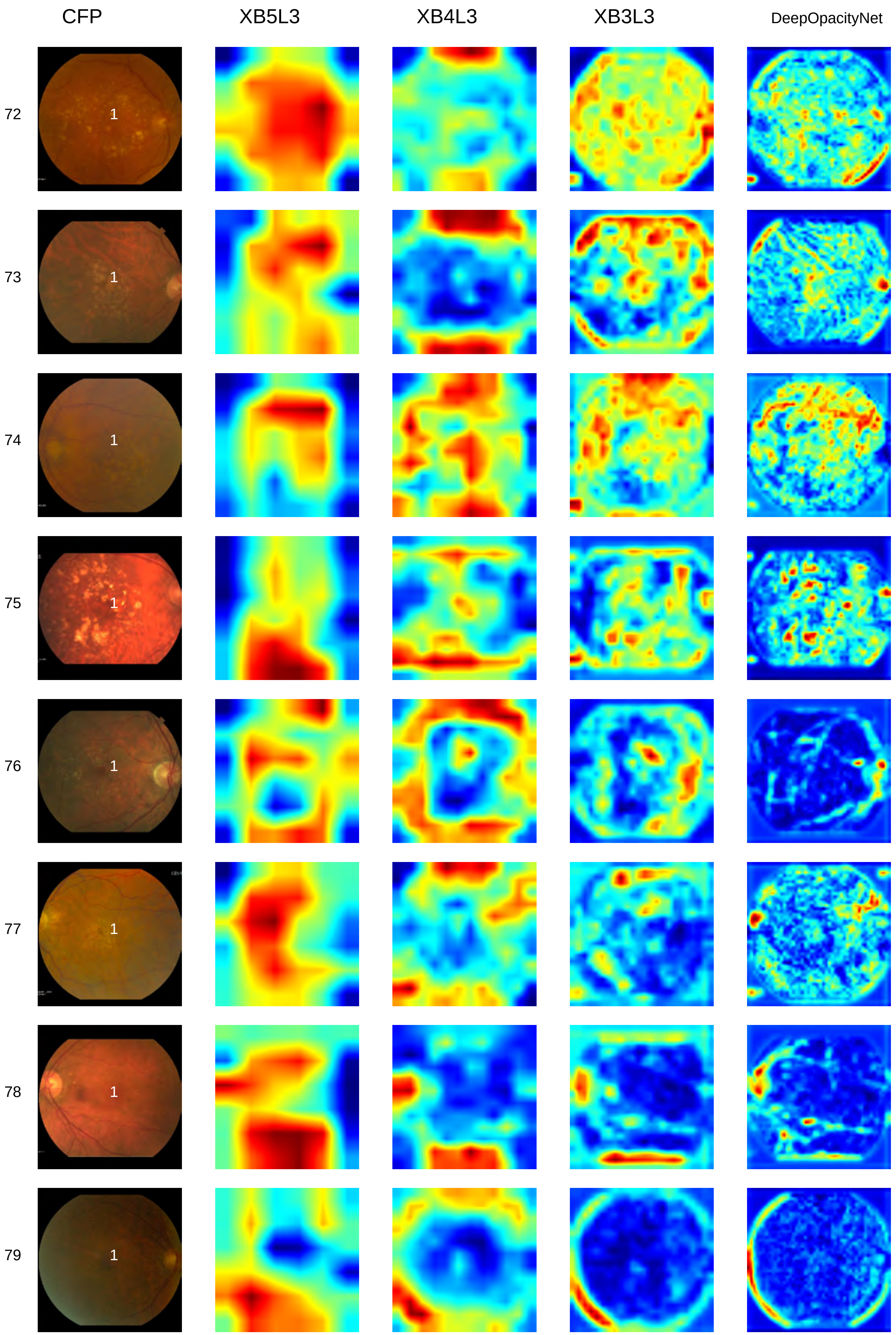
CFP
XB5L3
XB4L3
XB3L3
DeepOpacityNet
72
73
74
75
76
77
78
79
1
1
1
1
1
1
1
1

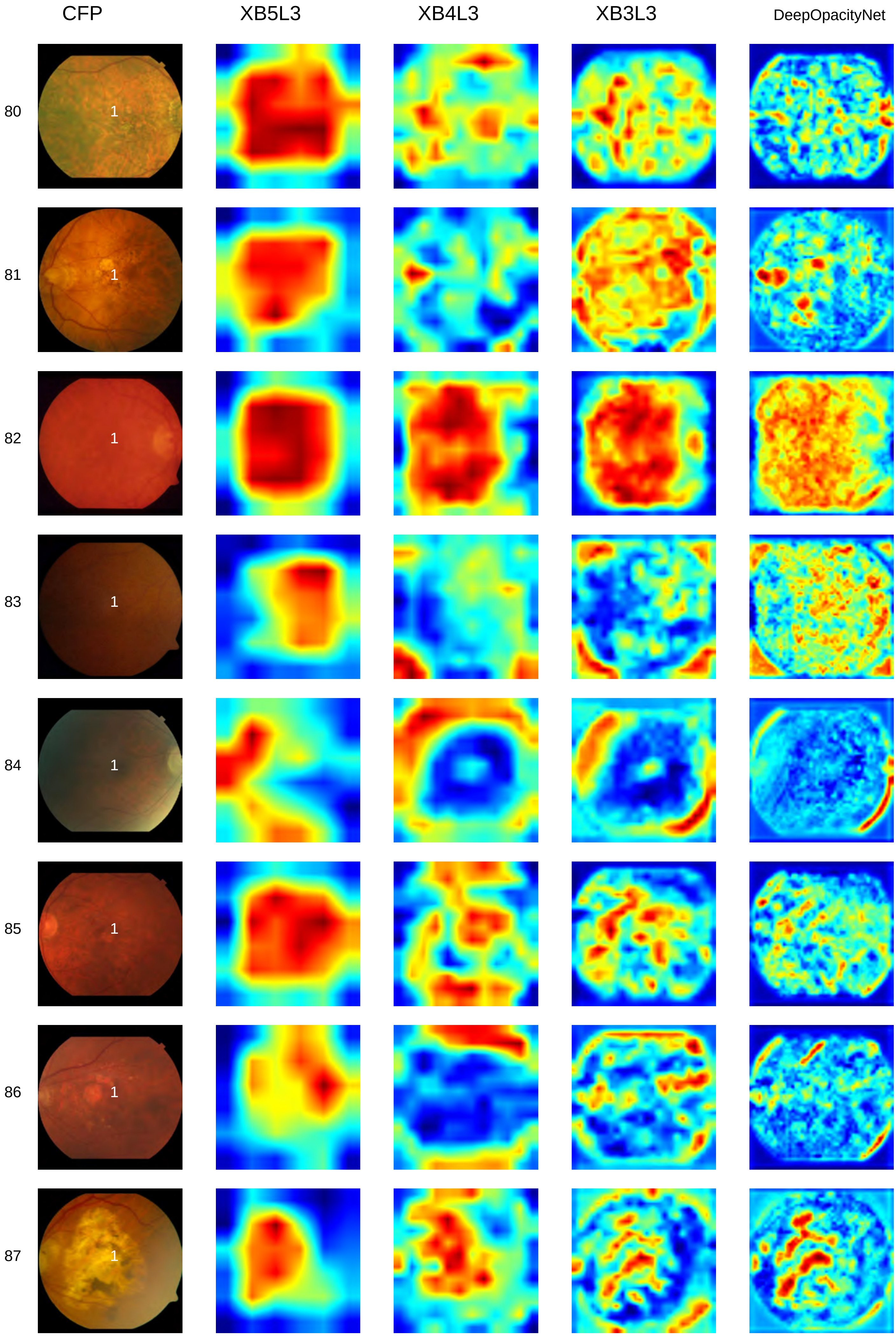

CFP
XB5L3
XB4L3
XB3L3
DeepOpacityNet
80
81
82
83
84
85
86
87
1

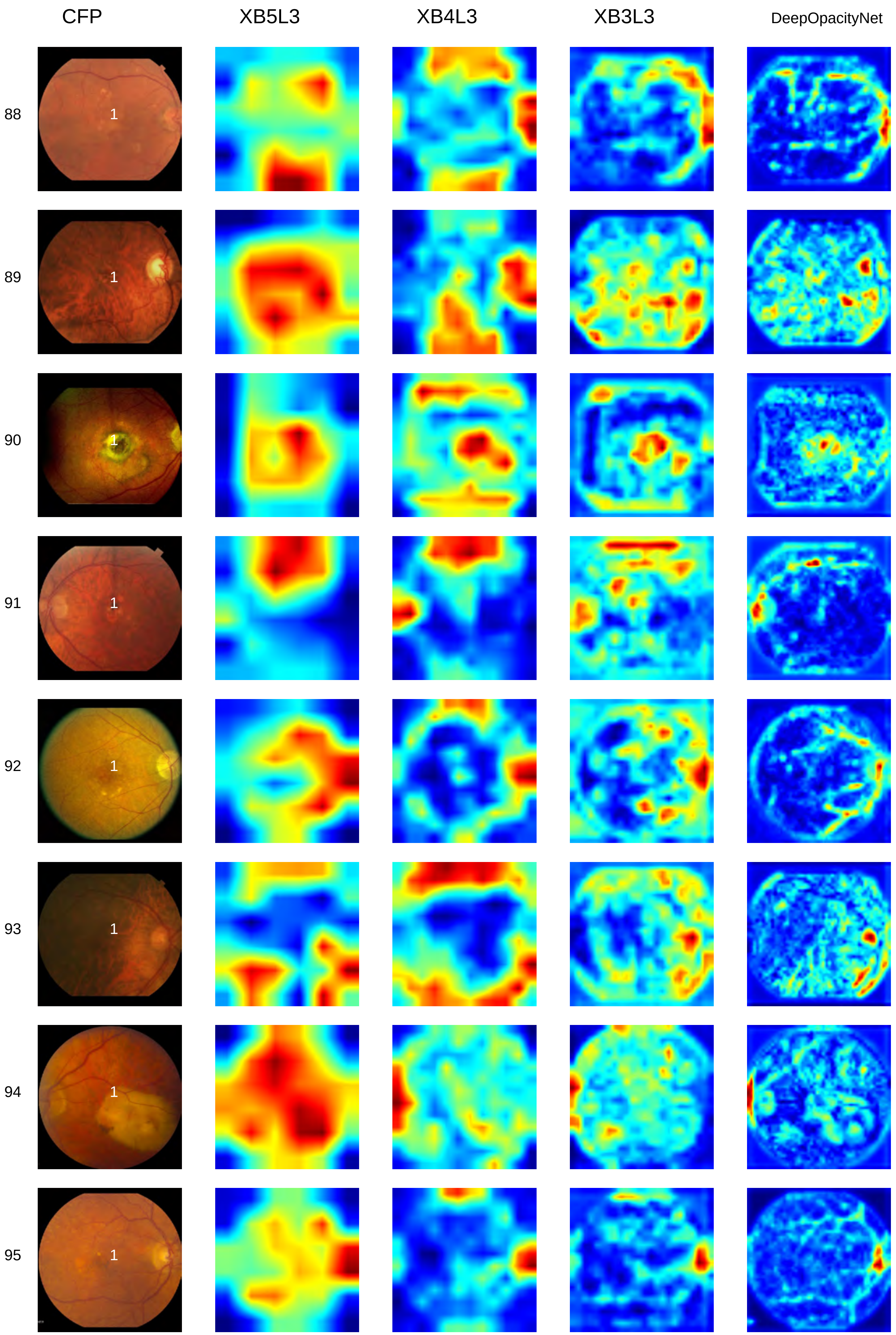

CFP
XB5L3
XB4L3
XB3L3
DeepOpacityNet
88
89
90
91
92
93
94
95
1

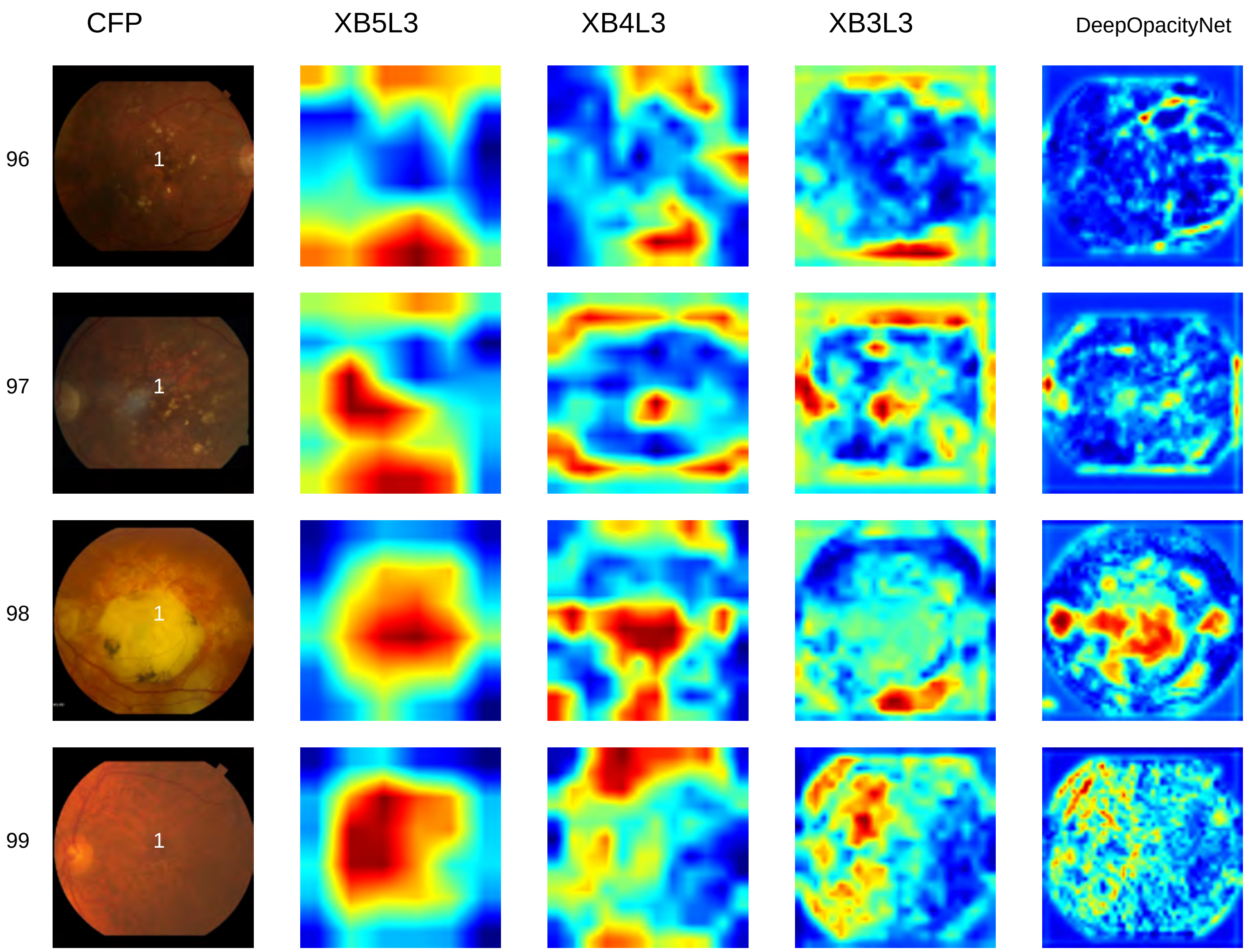
CFP
XB5L3
XB4L3
XB3L3
DeepOpacityNet
96
1
97
1
98
1
99
1

# Full Visualization of
# Transfer Learning Networks

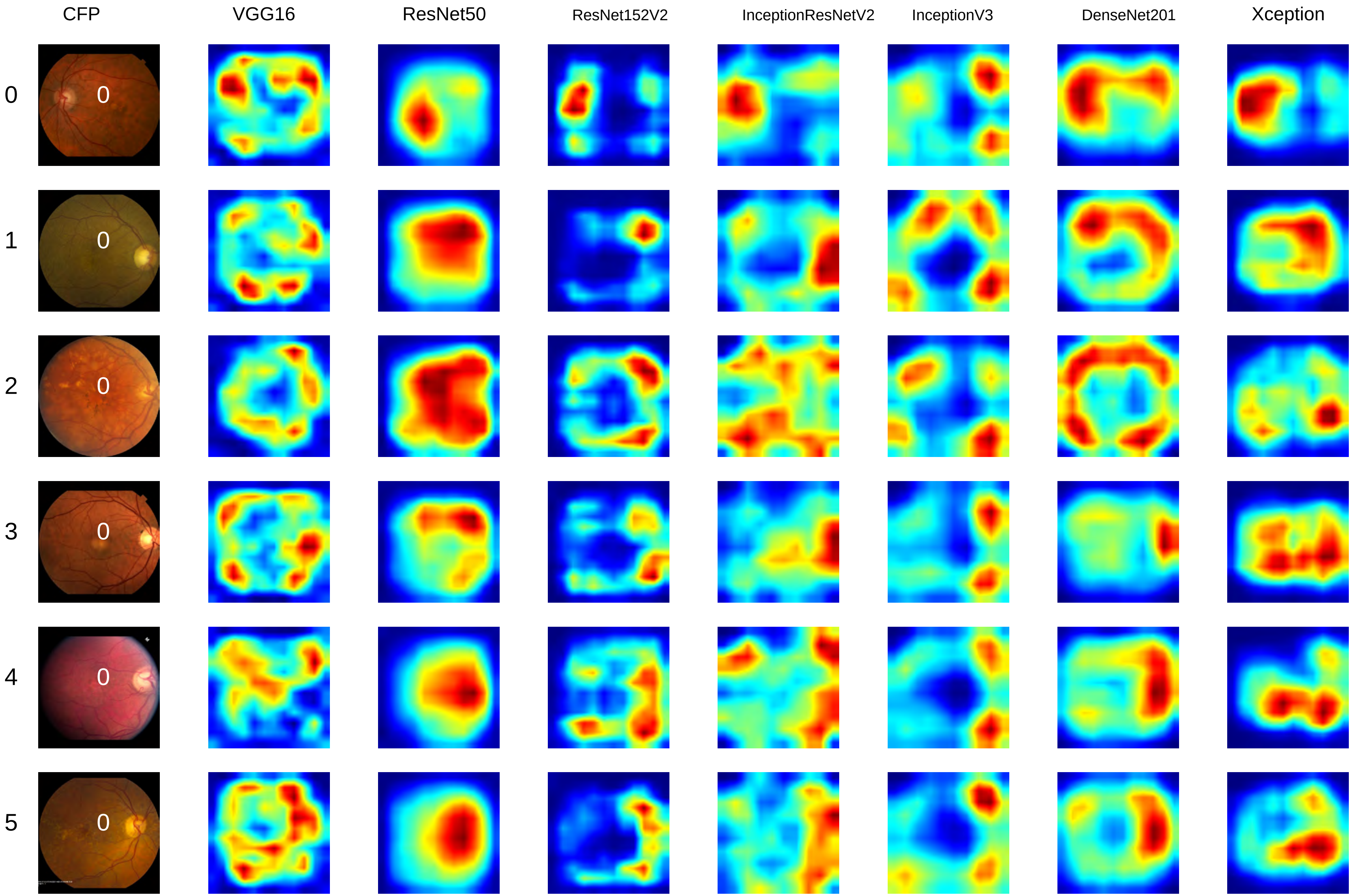
CFP
VGG16
ResNet50
ResNet152V2
InceptionResNetV2
InceptionV3
DenseNet201
Xception
0
1
2
3
4
5
0
0
0
0
0
0

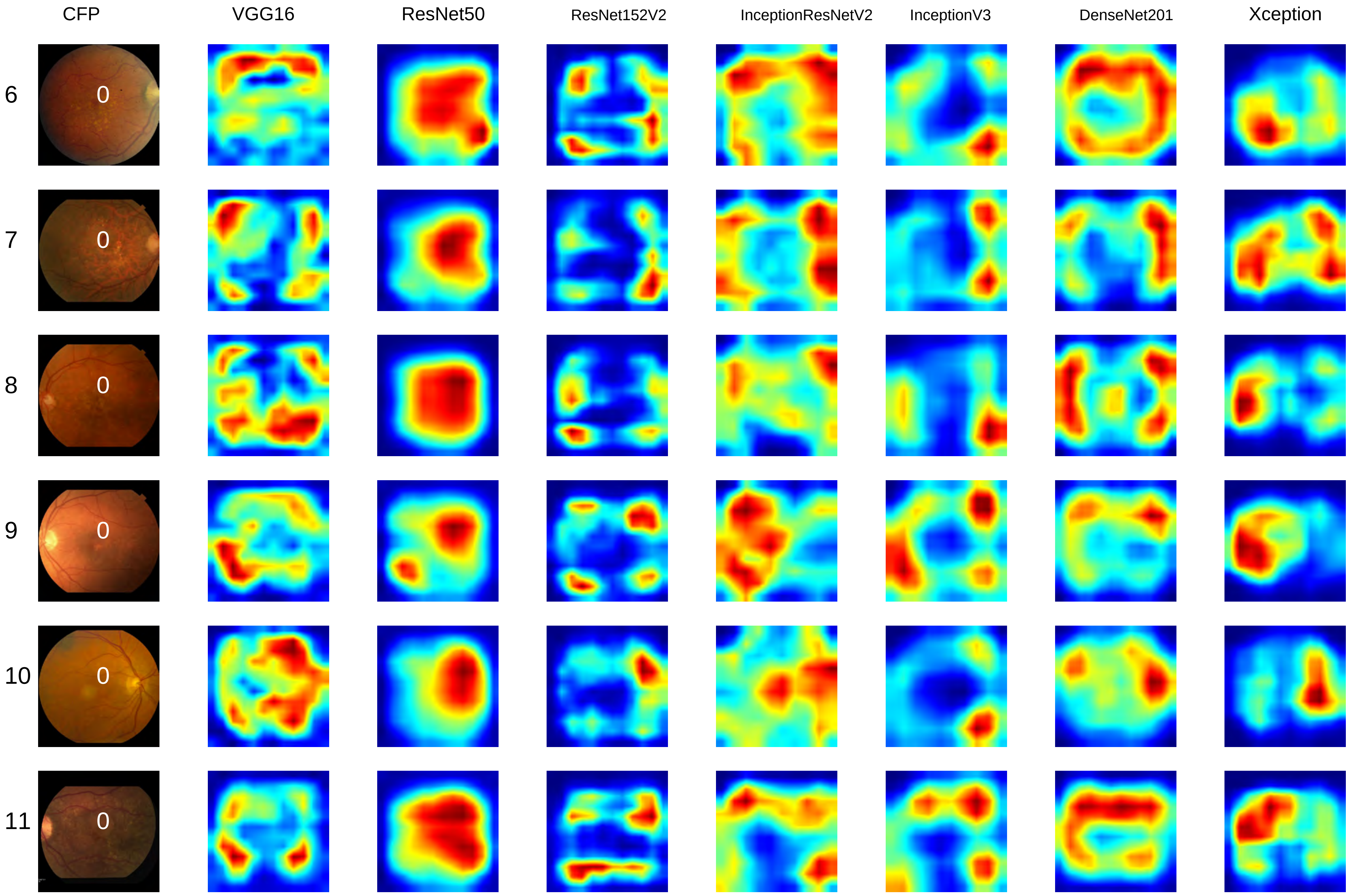

CFP
VGG16
ResNet50
ResNet152V2
InceptionResNetV2
InceptionV3
DenseNet201
Xception
6
0
7
0
8
0
9
0
10
0
11
0

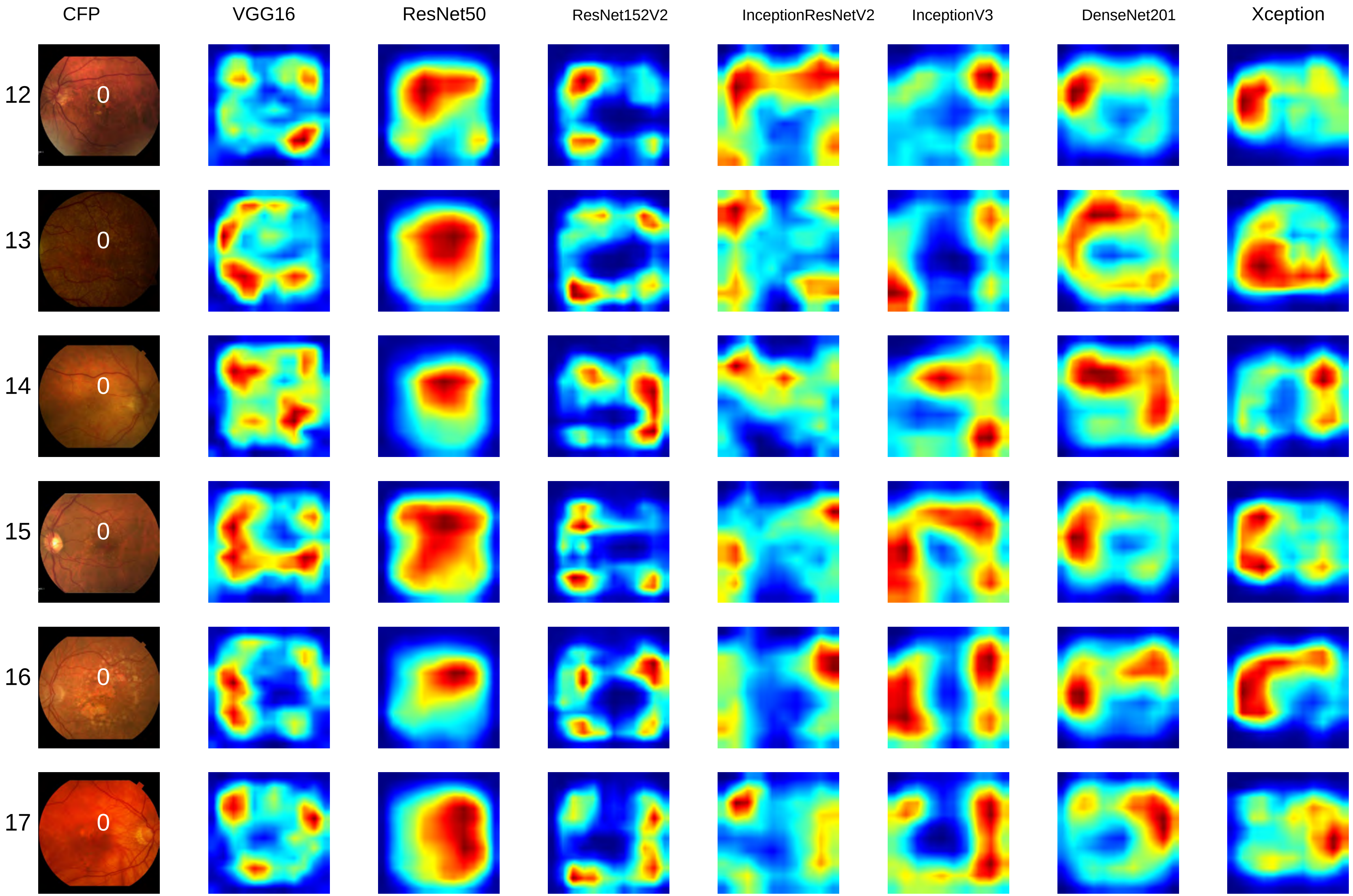
CFP
VGG16
ResNet50
ResNet152V2
InceptionResNetV2
InceptionV3
DenseNet201
Xception
12
0
13
0
14
0
15
0
16
0
17
0

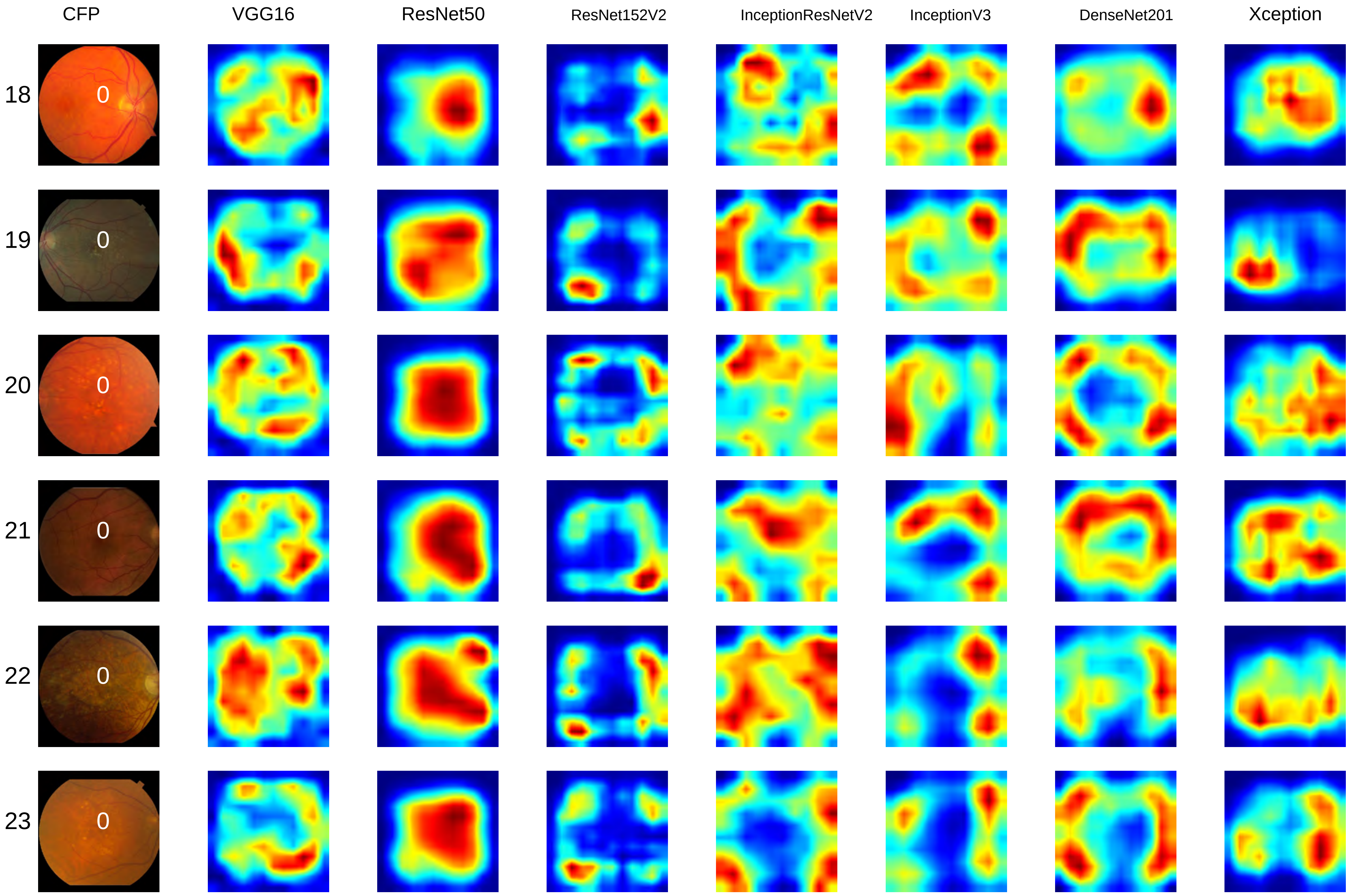

CFP
VGG16
ResNet50
ResNet152V2
InceptionResNetV2
InceptionV3
DenseNet201
Xception
18
0
19
0
20
0
21
0
22
0
23
0

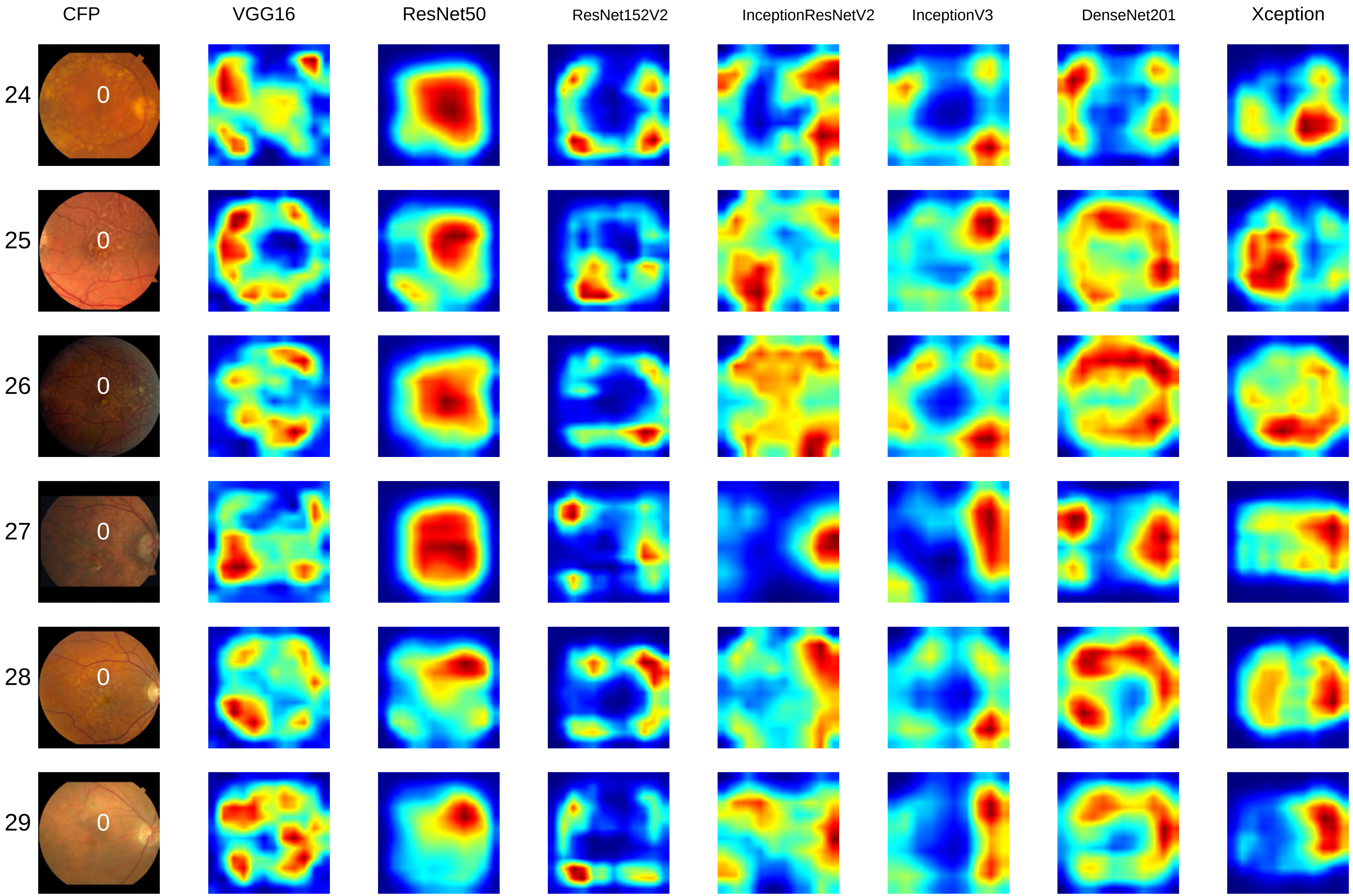
CFP
VGG16
ResNet50
ResNet152V2
InceptionResNetV2
InceptionV3
DenseNet201
Xception
24
0
25
0
26
0
27
0
28
0
29
0

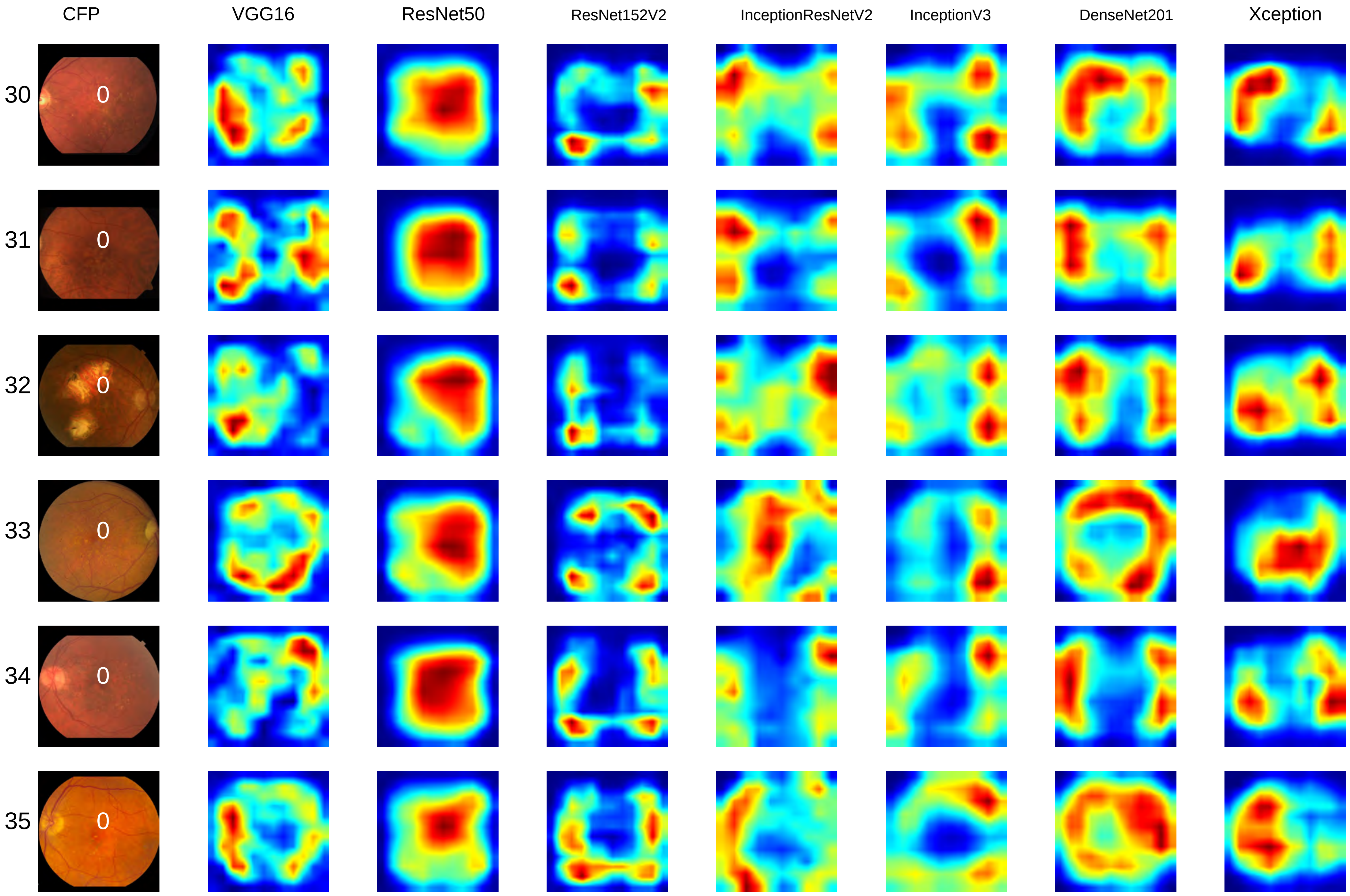
CFP
VGG16
ResNet50
ResNet152V2
InceptionResNetV2
InceptionV3
DenseNet201
Xception
30
0
31
0
32
0
33
0
34
0
35
0

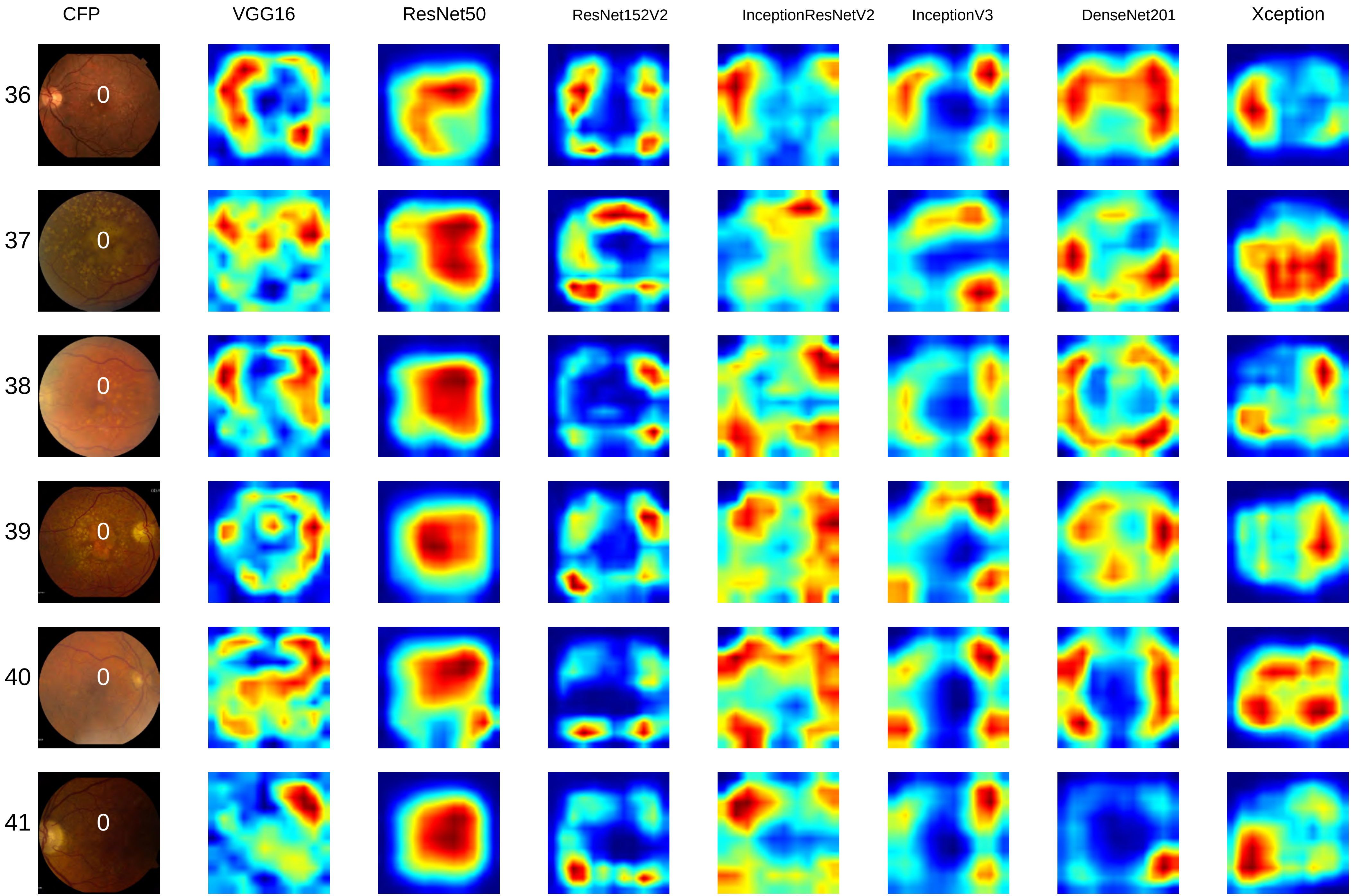
CFP
VGG16
ResNet50
ResNet152V2
InceptionResNetV2
InceptionV3
DenseNet201
Xception
36
37
38
39
40
41
0
0
0
0
0
0

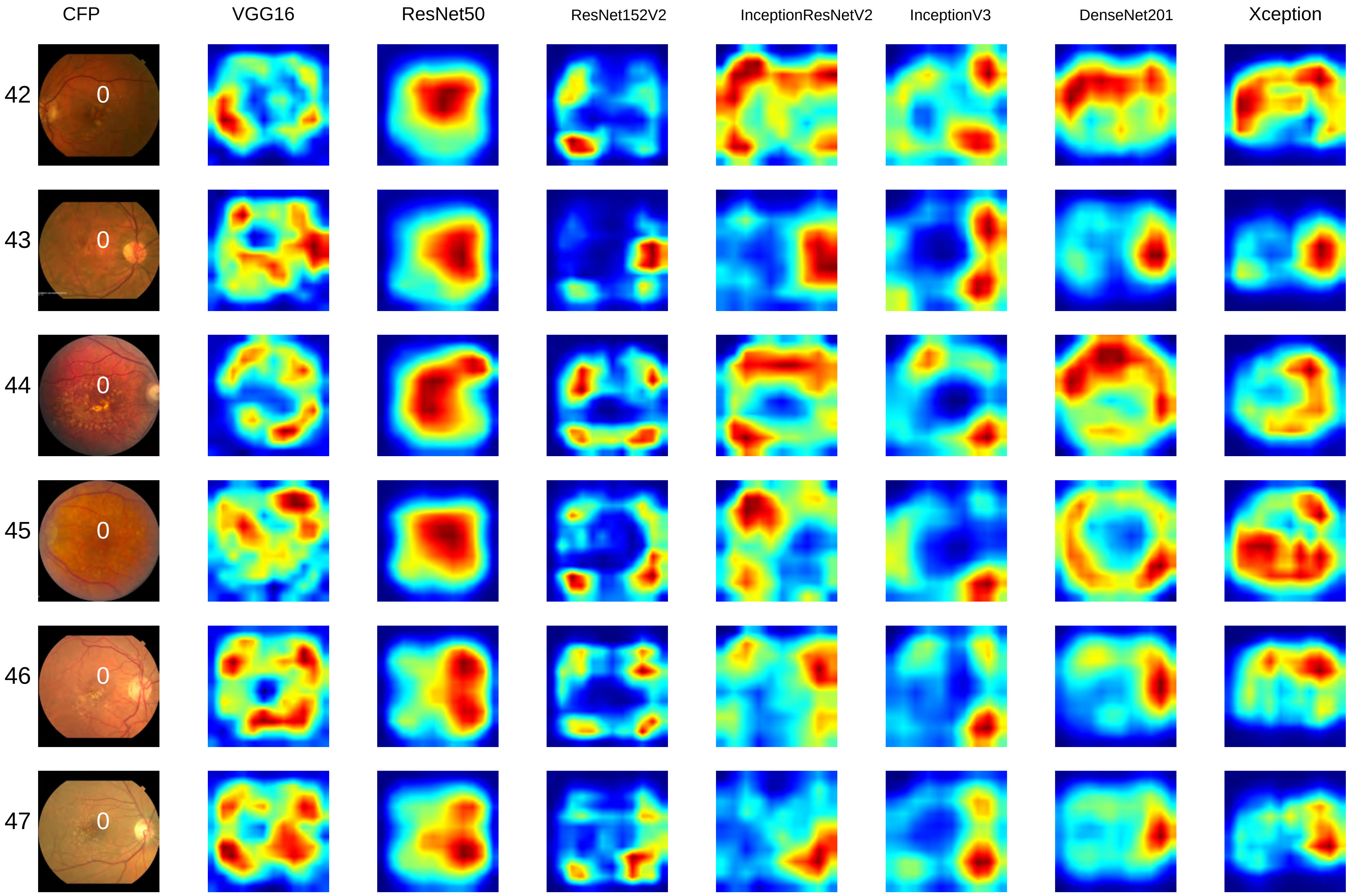
CFP
VGG16
ResNet50
ResNet152V2
InceptionResNetV2
InceptionV3
DenseNet201
Xception
42
0
43
0
44
0
45
0
46
0
47
0

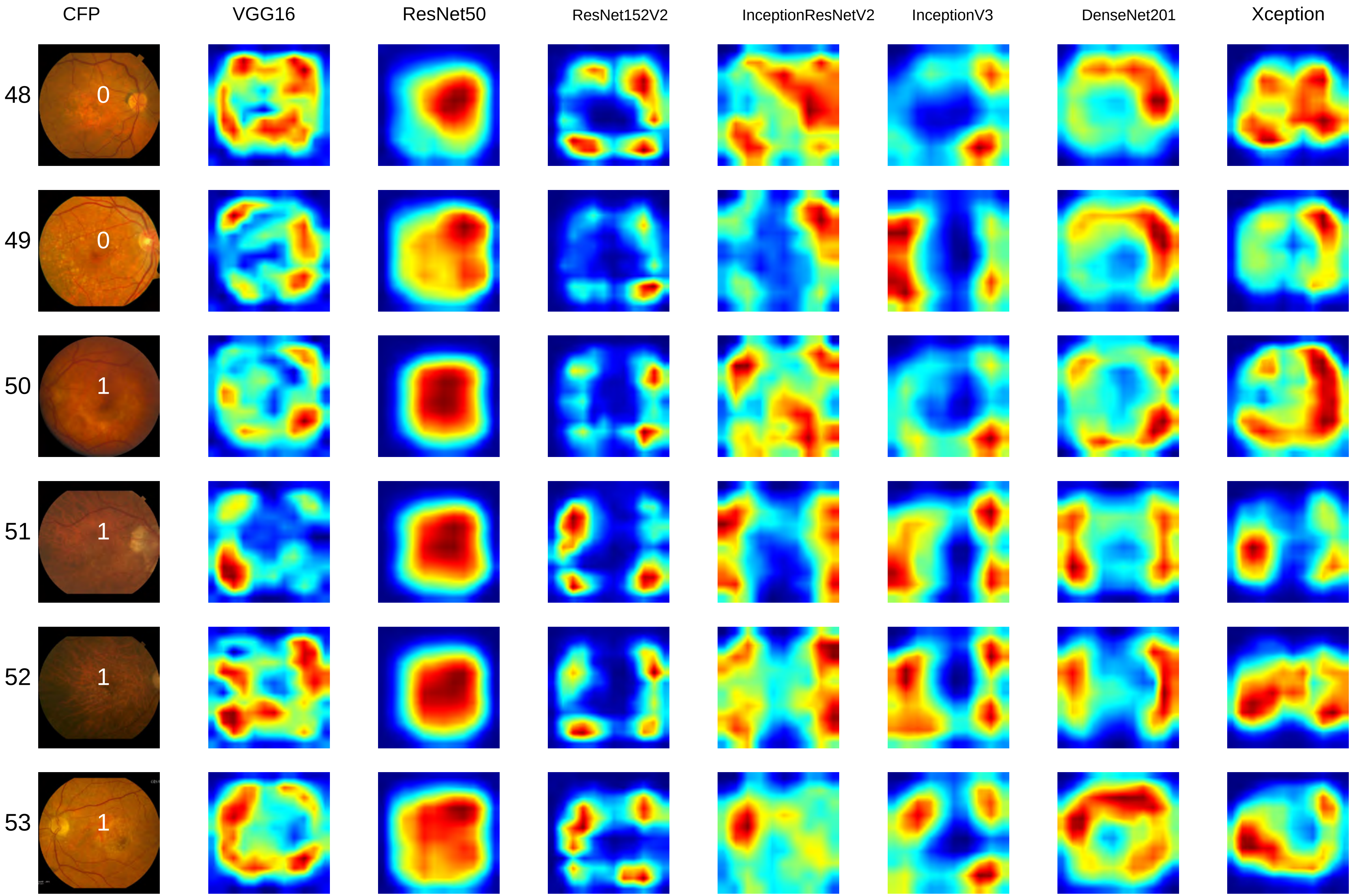
CFP
VGG16
ResNet50
ResNet152V2
InceptionResNetV2
InceptionV3
DenseNet201
Xception
48
0
49
0
50
1
51
1
52
1
53
1

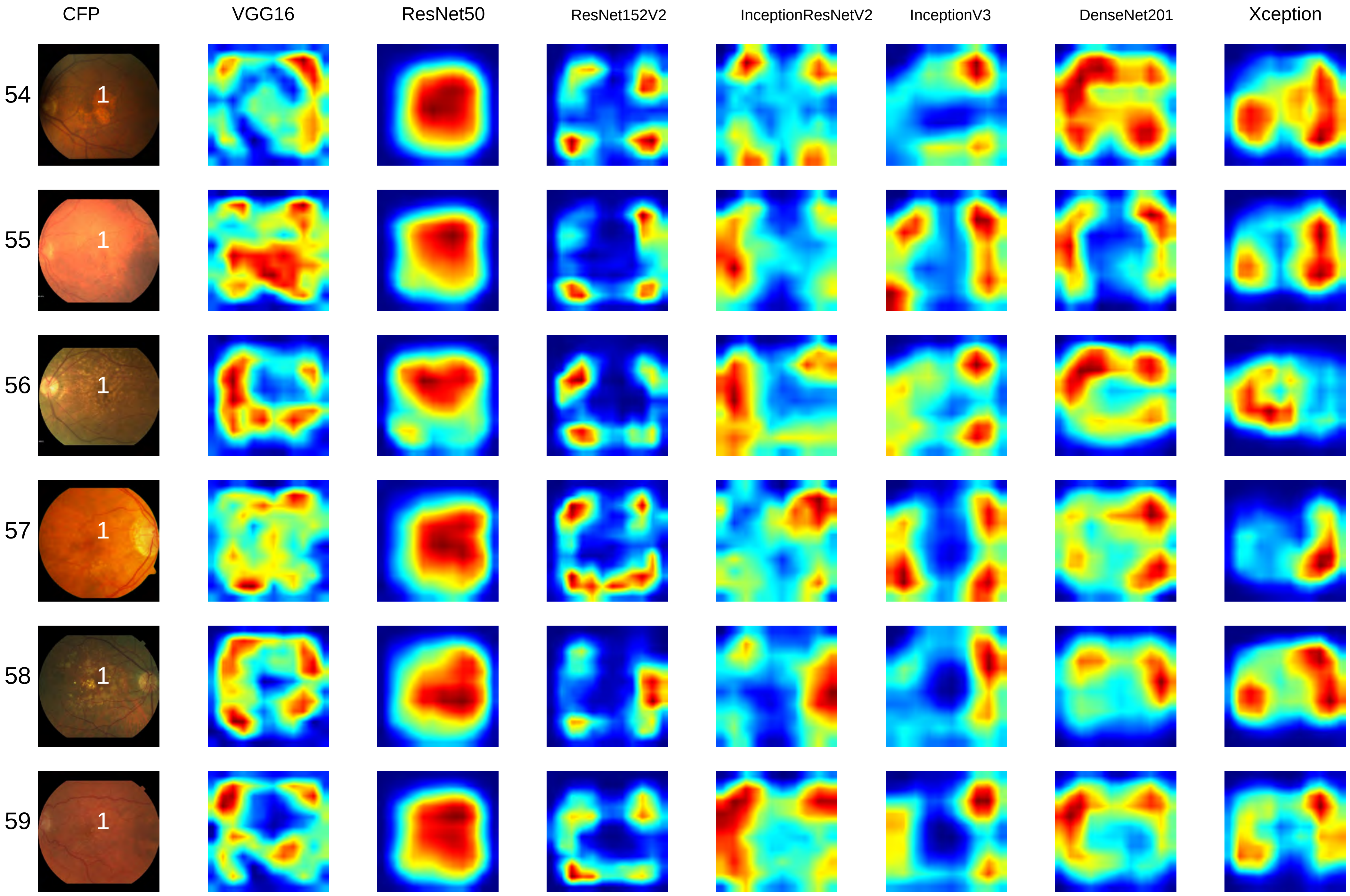
CFP
VGG16
ResNet50
ResNet152V2
InceptionResNetV2
InceptionV3
DenseNet201
Xception
54
55
56
57
58
59
1
1
1
1
1
1

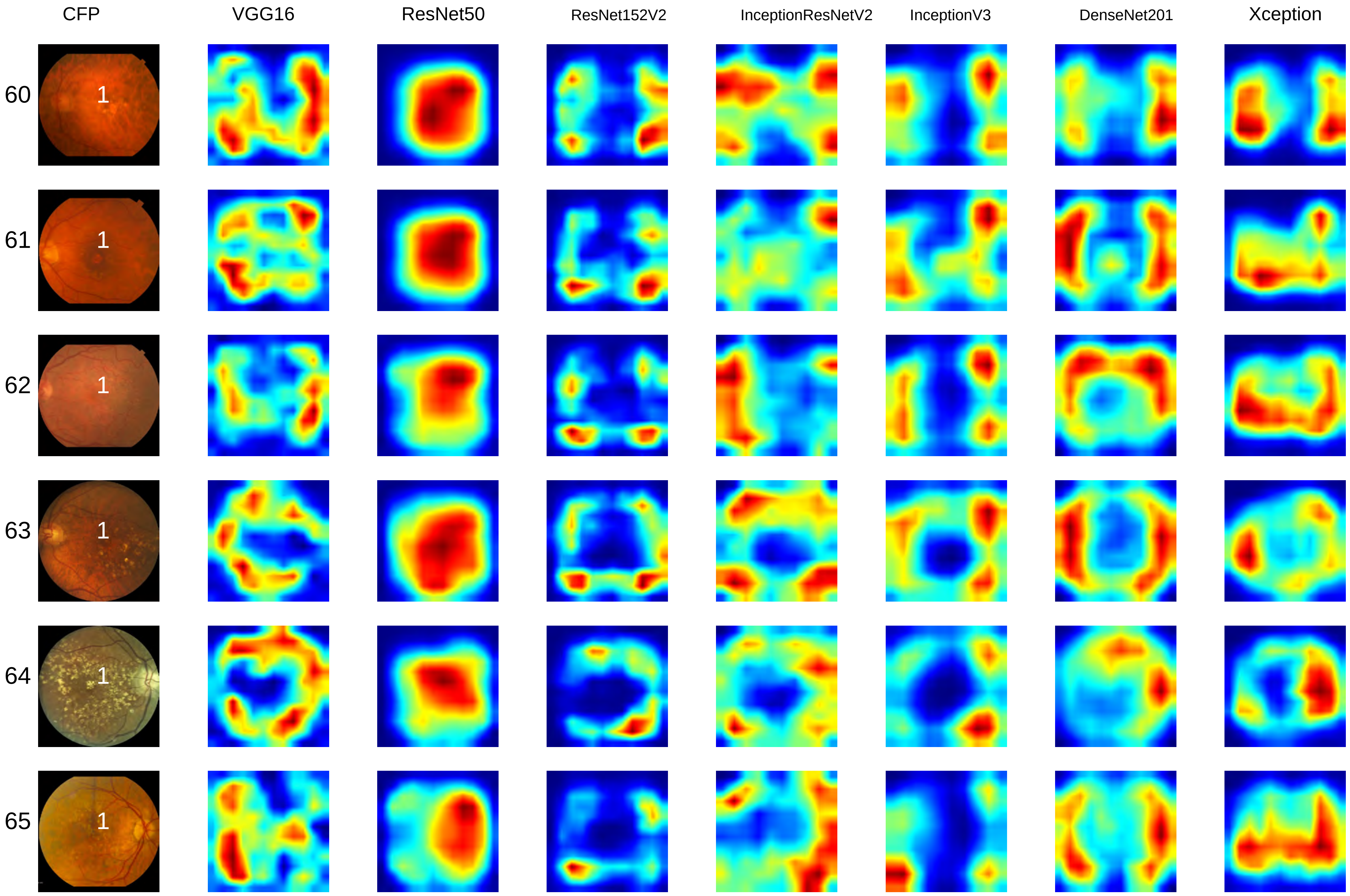
CFP
VGG16
ResNet50
ResNet152V2
InceptionResNetV2
InceptionV3
DenseNet201
Xception
60
61
62
63
64
65
1

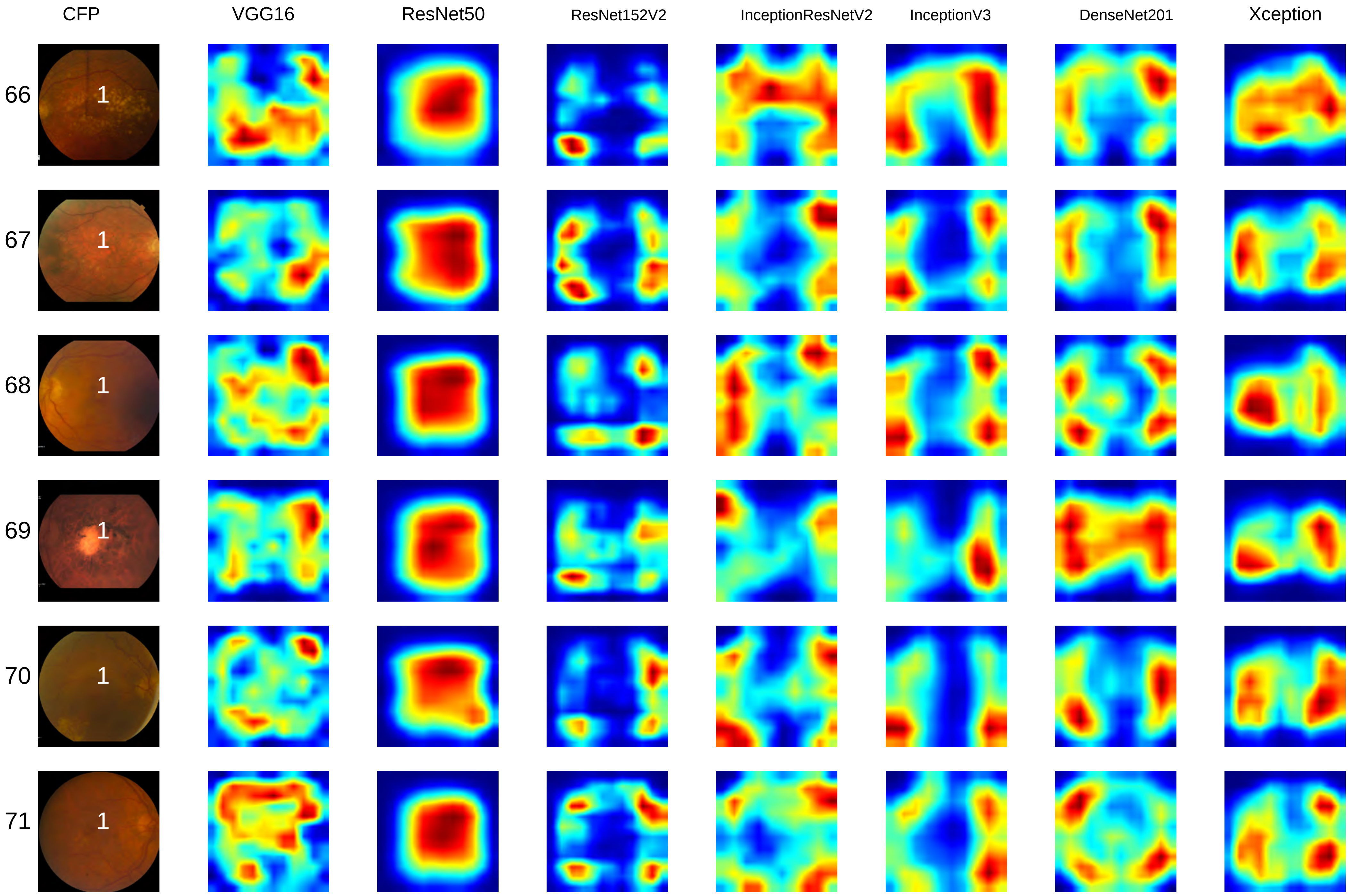
CFP
VGG16
ResNet50
ResNet152V2
InceptionResNetV2
InceptionV3
DenseNet201
Xception
66
67
68
69
70
71
1

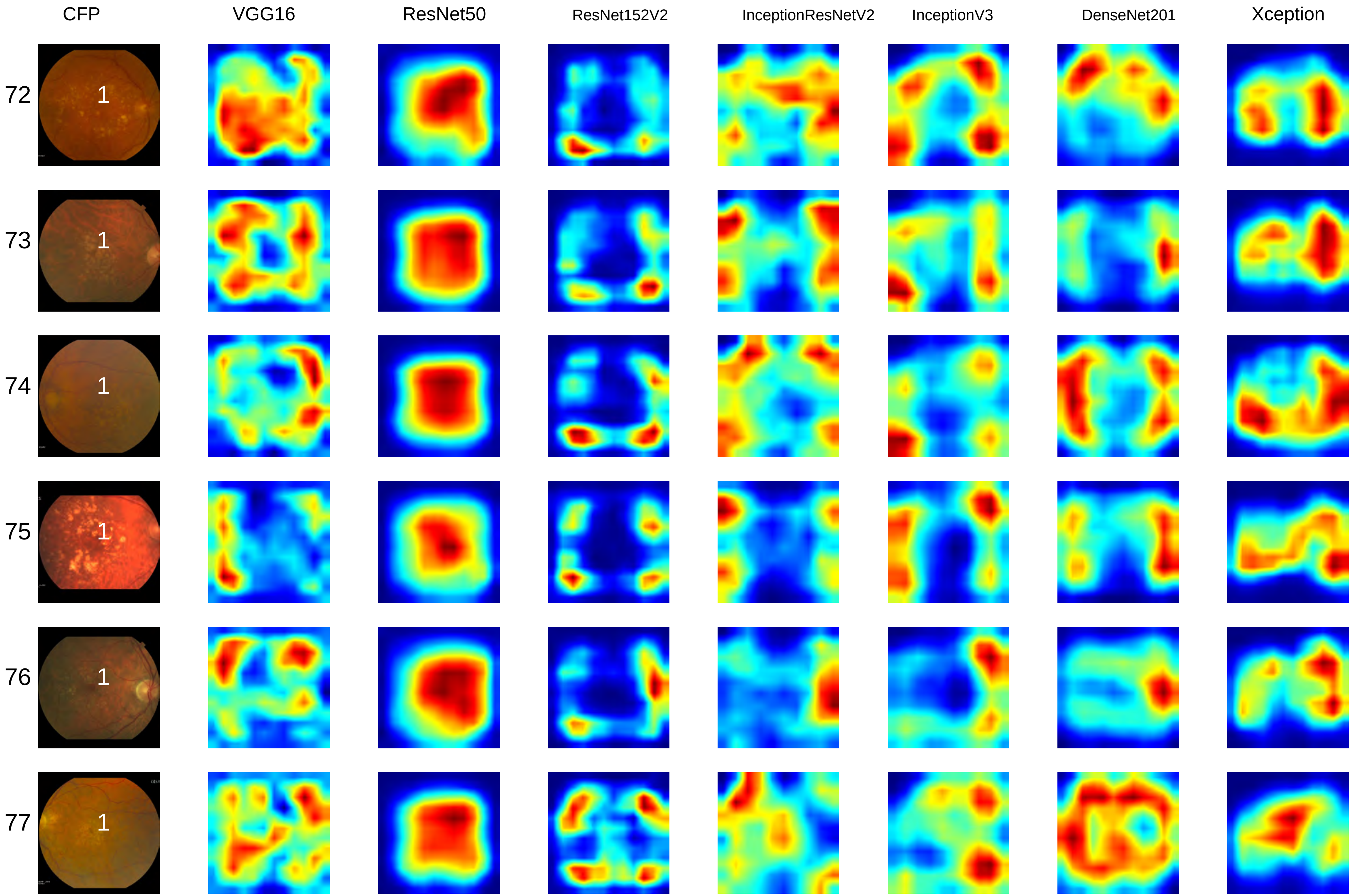
CFP
VGG16
ResNet50
ResNet152V2
InceptionResNetV2
InceptionV3
DenseNet201
Xception
72
73
74
75
76
77
1
1
1
1
1
1

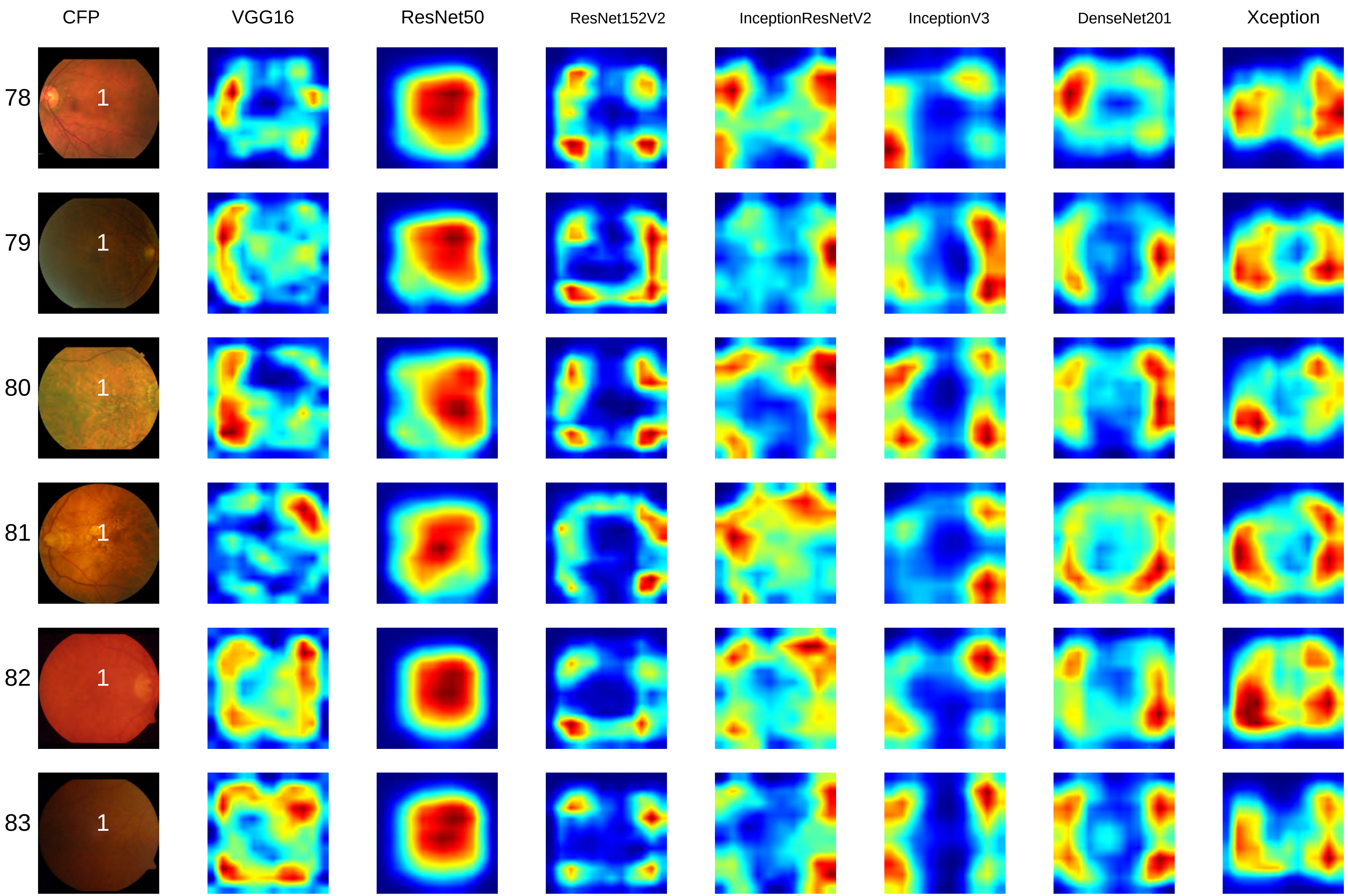
CFP
VGG16
ResNet50
ResNet152V2
InceptionResNetV2
InceptionV3
DenseNet201
Xception
78
79
80
81
82
83
1
1
1
1
1
1

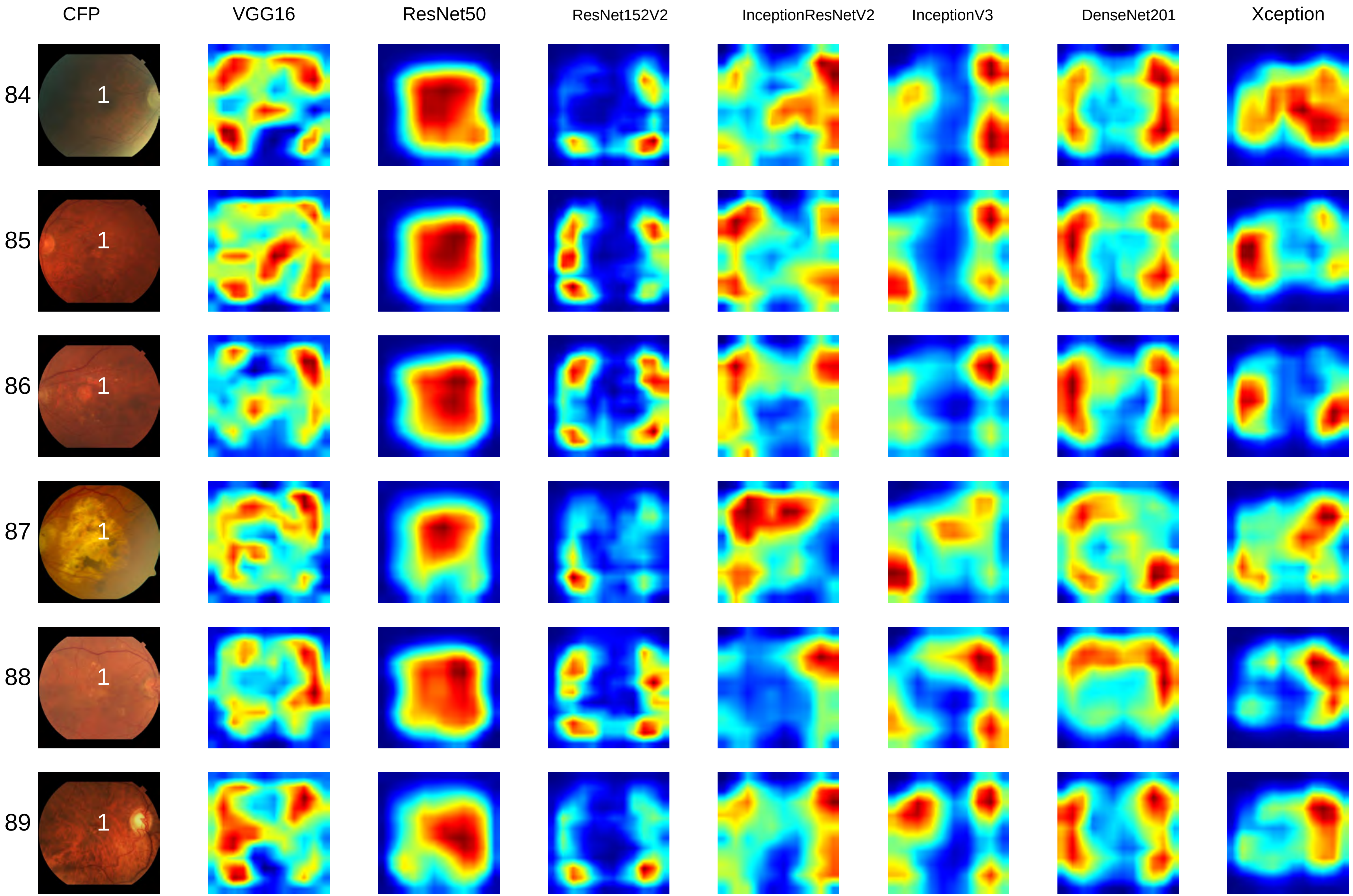

CFP
VGG16
ResNet50
ResNet152V2
InceptionResNetV2
InceptionV3
DenseNet201
Xception
84
85
86
87
88
89
1
1
1
1
1
1

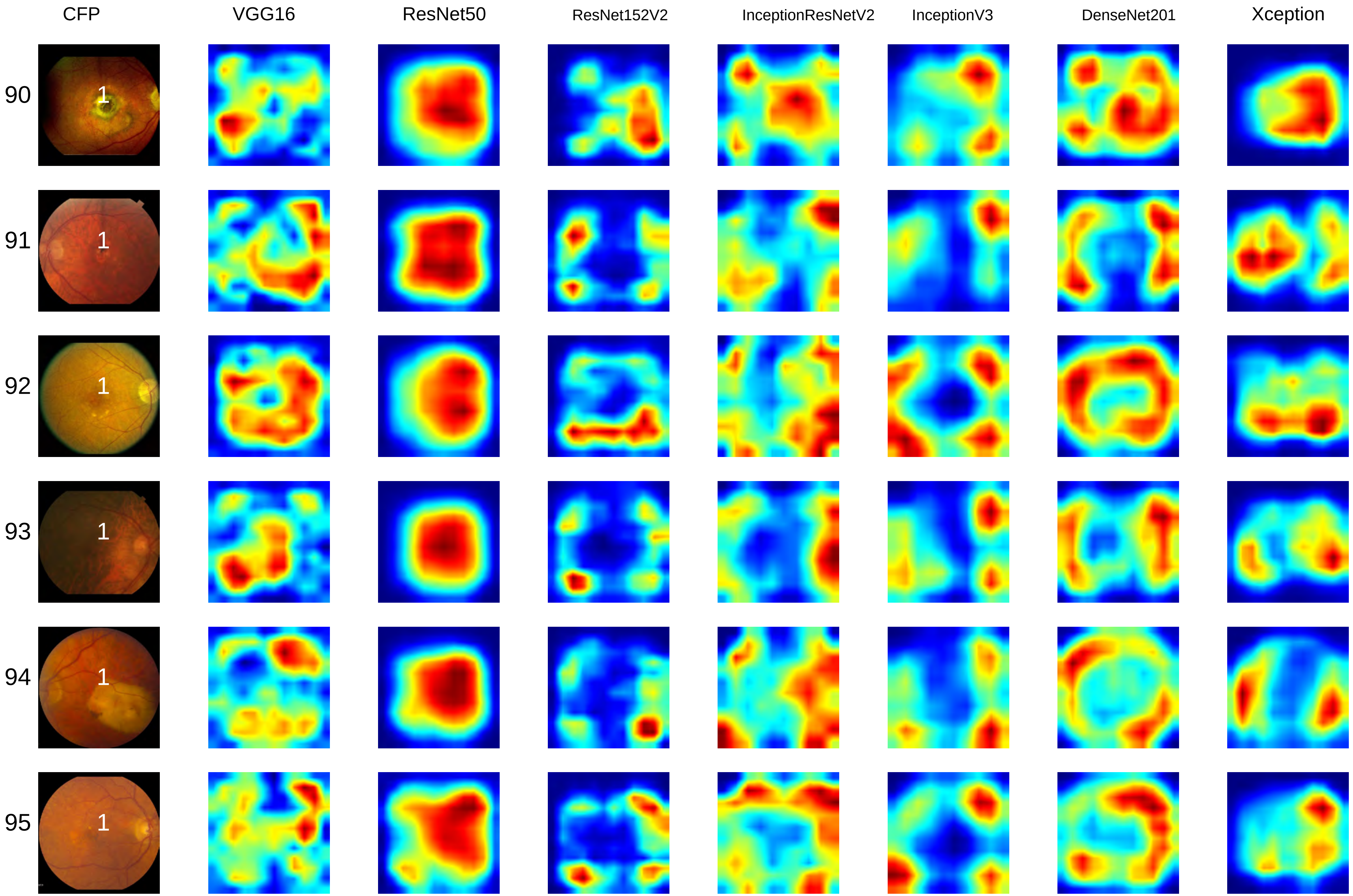
CFP
VGG16
ResNet50
ResNet152V2
InceptionResNetV2
InceptionV3
DenseNet201
Xception
90
91
92
93
94
95
1
1
1
1
1
1

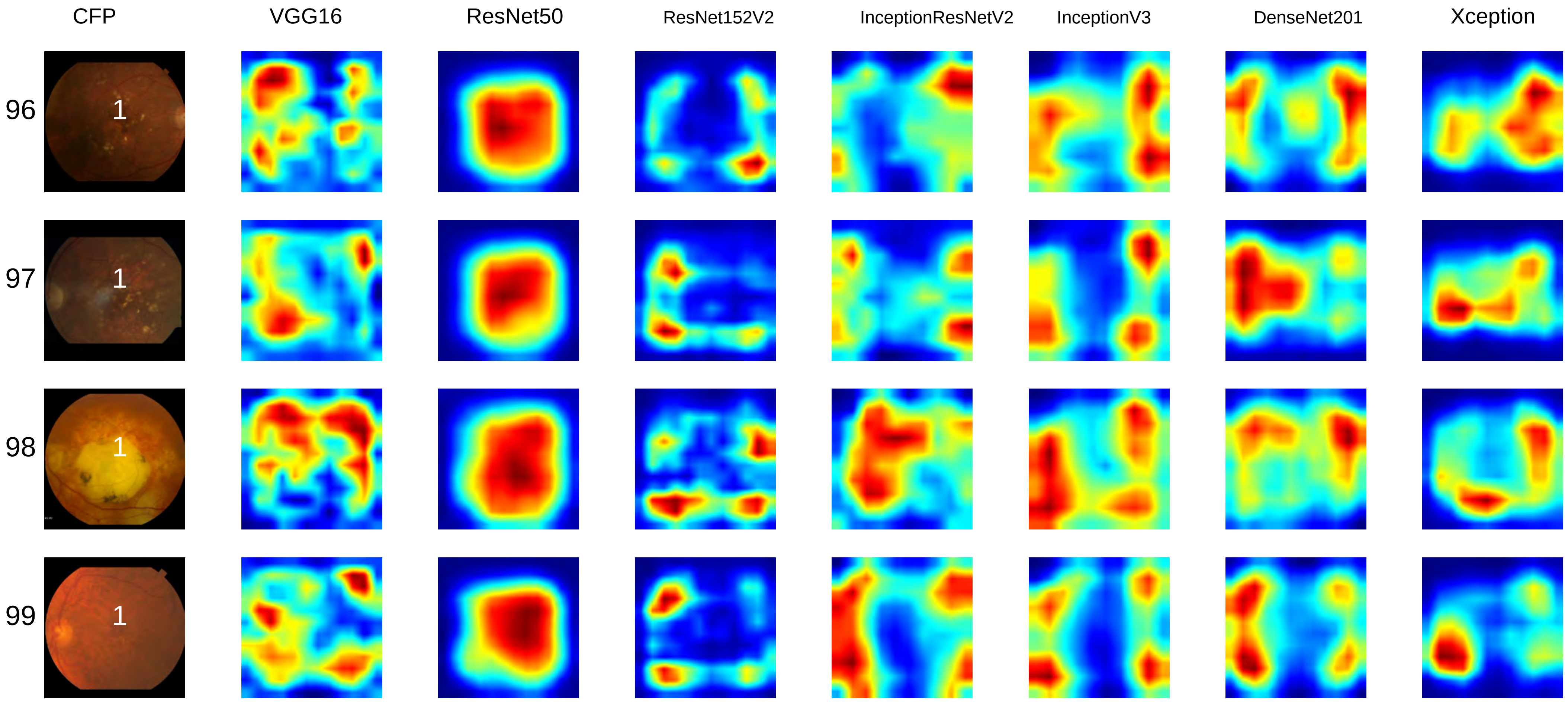

CFP
VGG16
ResNet50
ResNet152V2
InceptionResNetV2
InceptionV3
DenseNet201
Xception
96
1
97
1
98
1
99
1
